# Efficacy of homoeopathic treatment: Systematic review of meta-analyses of randomised placebo-controlled homoeopathy trials for any indication

**DOI:** 10.1186/s13643-023-02313-2

**Published:** 2023-10-07

**Authors:** H. J. Hamre, A. Glockmann, K. von Ammon, D. S. Riley, H. Kiene

**Affiliations:** 1 Institute for Applied Epistemology and Medical Methodology at Witten/Herdecke University (IFAEMM), Freiburg, Germany; 2 Faculty of Health, Department of Medicine, Chair of Medical Theory, Integrative and Anthroposophic Medicine, Witten/Herdecke University, Witten, Germany; 3 Maryland University of Integrative Health (MUIH), Laurel, MD USA; 4 Homeopathic Pharmacopoeia Convention of the United States (HPCUS), Southeastern, PA USA

## Abstract

**Background and objective:**

Since 1997, several meta-analyses (MAs) of placebo-controlled randomised efficacy trials of homoeopathy for any indication (PRETHAIs) have been published with different methods, results and conclusions. To date, a formal assessment of these MAs has not been performed. The main objective of this systematic review of MAs of PRETHAIs was to evaluate the efficacy of homoeopathic treatment.

**Methods:**

The inclusion criteria were as follows: MAs of PRETHAIs in humans; all ages, countries, settings, publication languages; and MAs published from 1 Jan. 1990 to 30 Apr. 2023. The exclusion criteria were as follows: systematic reviews without MAs; MAs restricted to age or gender groups, specific indications, or specific homoeopathic treatments; and MAs that did not assess efficacy. We searched 8 electronic databases up to 14 Dec. 2020, with an update search in 6 databases up to 30 April 2023.

The primary outcome was the effect estimate for all included trials in each MA and after restricting the sample to trials with high methodological quality, according to predefined criteria. The risk of bias for each MA was assessed by the ROBIS (Risk Of Bias In Systematic reviews) tool. The quality of evidence was assessed by the GRADE framework. Statistical analyses were performed to determine the proportion of MAs showing a significant positive effect of homoeopathy vs. no significant difference.

**Results:**

Six MAs were included, covering individualised homoeopathy (I-HOM, *n* = 2), nonindividualised homoeopathy (NI-HOM, *n* = 1) and all homoeopathy types (ALL-HOM = I-HOM + NI-HOM, *n* = 3). The MAs comprised between 16 and 110 trials, and the included trials were published from 1943–2014. The median trial sample size ranged from 45 to 97 patients. The risk of bias (low/unclear/high) was rated as low for three MAs and high for three MAs.

Effect estimates for all trials in each MA showed a significant positive effect of homoeopathy compared to placebo (5 of 5 MAs, no data in 1 MA). Sensitivity analyses with sample restriction to high-quality trials were available from 4 MAs; the effect remained significant in 3 of the MAs (2 MAs assessed ALL-HOM, 1 MA assessed I-HOM) and was no longer significant in 1 MA (which assessed NI-HOM).

**Discussion:**

The quality of evidence for positive effects of homoeopathy beyond placebo (high/moderate/low/very low) was high for I-HOM and moderate for ALL-HOM and NI-HOM. There was no support for the alternative hypothesis of no outcome difference between homoeopathy and placebo.

The available MAs of PRETHAIs reveal significant positive effects of homoeopathy beyond placebo. This is in accordance with laboratory experiments showing partially replicable effects of homoeopathically potentised preparations in physico-chemical, in vitro, plant-based and animal-based test systems.

**Systematic review registration:**

PROSPERO CRD42020209661. The protocol for this SR was finalised and submitted on 25 Nov. 2020 and registered on 26 Dec. 2020.

**Supplementary Information:**

The online version contains supplementary material available at 10.1186/s13643-023-02313-2.

## Background and rationale

 Homoeopathy is a therapy system widely used in Europe, India and other countries [1]. Core features of homoeopathy include drug provings (observation of symptoms occurring in healthy persons exposed to substances of mineral, botanical or zoological origin), simile principle (similarity between symptom patterns in drug provings and the symptoms to be treated with the same substance) and potentization (successive dilution of the homoeopathic substance, with each dilution step involving repeated shaking of liquids or grinding of solids into lactose) [2].

The clinical effects of homoeopathic treatment have been investigated in several hundred randomised controlled trials [3] and in systematic reviews (SRs). Among the SRs, two contrasting approaches can be discerned.

One approach is to focus on a specific indication (e.g., depression [4], acute respiratory tract infections in children [5]) while often including open-label trials and observational studies. In this approach, data synthesis is grouped by design, thus yielding information about homoeopathy in patient care.

The opposite approach is to include all indications while restricting study designs to placebo-controlled trials and aggregating results in an MAs, thus yielding information about the specific effects of homoeopathy beyond those of placebo. A major reason for using this approach has been the claim that ‘homoeopathy violates natural laws and thus any effect must be a placebo effect’ [6].

Since 1997, at least six MAs of placebo-controlled homoeopathy trials for any condition have been published [6–11]. These MAs have differed in their methods for trial inclusion, data synthesis and assessment of risk of bias; furthermore, their results and conclusions have been inconsistent. During this period, there have been substantial advancements in methodology and quality standards for MAs and other SRs [12–15], including SRs of SRs (also called overviews or umbrella reviews) [16–18]. To our knowledge, a formal SR of MAs of randomised placebo-controlled homoeopathy trials for any condition has not been performed. Herein, we report such an SR.

## Objectives

### Research questions

1. Does homoeopathic treatment have positive effects beyond placebo in MAs of randomised placebo-controlled trials for any condition?
2. Do the findings from these MAs support the notion of a common effect—or absence thereof—across different types of homoeopathic treatment (e.g. individualised, clinical or complex homoeopathy) and across different types of indications (e.g. acute, chronic)?

## Methods

### Eligibility criteria for meta-analyses (MAs)

The eligibility criteria are presented in Table 1.

**Table 1:** Eligibility criteria for meta-analyses

Feature	Include	Exclude
Design	MA of randomised controlled trials, including secondary analyses thereof	Narrative reviews; systematic reviews without quantitative synthesis of treatment effect estimates, MA not based on randomised controlled trials
Patients and settings	Humans, no age restriction, any country, any setting	Veterinary trials, MA restricted to specific age or gender groups
Indications	MA covering any indication, disease or symptom	MA restricted to specific indications, indication groups or clinical domains
Interventions	Homoeopathy, defined as: a) Prevention or treatment with homoeopathic medicinal products^a^, i.e. products manufactured by a method described in a homoeopathic pharmacopoeia (mandatory) b) Homoeopathic case-taking (optional) [19]	Any other new intervention (but continuation of ongoing therapy does not lead to exclusion) Homoeopathic case taking without use of homoeopathic medicinal products MA restricted to specific homoeopathic products or product groups
Comparators	Placebo	MA not including placebo-controlled trials
Outcomes	MA of therapeutic benefit, measured by any clinically relevant outcome	MA not including therapeutic benefit (e.g. use or safety only)
Report time frame	MA publications from 1 Jan. 1990 up to 30 Apr. 2023	MA publications after 30 Apr. 2023
Report language	Any language	
Publication, general aspects	All three criteria (a–c) must be fulfilled: a) written and dated reports with identifiable authors b) which are or have been in the public domain OR have been submitted to a third party c) with presentation of methods and results in sufficient detail, allowing for assessment of the research questions in a meaningful way	
Publication subject	1. Protocol for a MA 2. Primary publication of a MA 3. Additional analyses: all criteria (a–c) must be fulfilled a) pertaining to a MA included in this systematic review b) presenting results not included in the primary MA publication c) contributing to the assessment of the research questions in a meaningful way	

*MA* meta-analysis

^a^Medicinal products: sometimes the terms ‘remedies’ or ‘medicines’ are used

### Information sources and search strategy

#### Databases

We searched eight online databases, including four databases largely or totally restricted to SRs (A–D), two generic databases (E–F) and two databases focused on complementary or alternative therapies (G–H) (Table 2). In addition, one private database (author HJH) was searched.

**Table 2:** Online databases and search strategies

Database, URL	Search strategy
A. Cochrane Database of Systematic Reviews https://www.cochranelibrary.com/cdsr/about-cdsr	MeSH DESCRIPTOR Homoeopathy EXPLODE ALL TREES
B. Database of Abstracts of Reviews of Effects (DARE) https://www.crd.york.ac.uk/CRDWeb/	MeSH DESCRIPTOR Homoeopathy EXPLODE ALL TREES
C. International Prospective Register of Systematic Reviews (PROSPERO) https://www.crd.york.ac.uk/prospero/	MeSH DESCRIPTOR Homoeopathy EXPLODE ALL TREES
D. Joanna Briggs Institute (JBI) Systematic Review Register https://joannabriggs.org/systematic-review-register	homoeopathy OR homoeopathy OR Homöopathie OR homoeopathic OR homoeopathic OR homöopathisch
E. PubMed https://www.ncbi.nlm.nih.gov/pubmed/	("meta-analysis"[Publication Type] OR "systematic review"[Publication Type]) AND "homoeopathy"[MeSH Terms]) AND "humans"[MeSH Terms])
F. Latin American and Caribbean Health Sciences Literature (LILACS) https://lilacs.bvsalud.org/en/	Filters applied (Main subject: Homoeopathy; Type of study: Systematic reviews)
G. Allied and Complementary Medicine Database (AMED) https://health.ebsco.com/products/amed-the-allied-and-complementary-medicine-database/complementary-alternative-medicine	(homoeopathy OR homoeopathy) AND TI (meta-analysis OR review OR placebo-controlled) NOT (veterinary OR animal) AND Filter: "Academic Journals"
H. CAMbase http://cambase.dmz.uni-wh.de/CiXbase/camdb/	Keyword (Homoeopathy OR homoeopathic OR homoeopathy OR homoeopathic) AND (systematic review OR meta-analysis)

#### Other sources

A list of included MAs was sent to experts in the field to identify any missing eligible MAs or additional analyses of the included MAs.

### Selection process

#### Screening

Two reviewers (HJH, AG) independently searched the online literature databases and screened the titles and abstracts to identify potentially eligible MAs. The reviewers compared their screening results, and discrepancies were resolved by discussion (HJH, AG).

#### Eligibility

For the potentially eligible MA records, full-text reports were obtained. Two reviewers (HJH, AG) independently read the full texts and assessed their eligibility in accordance with the eligibility criteria (Table 1). The reviewers compared their eligibility assessments, and discrepancies were resolved by discussion (HJH, AG).

### Data collection process

Two reviewers independently extracted data from the full-text reports into Excel files (HJH + [GSK, HK or AG]) using a piloted data extraction form. Reviewer AG compared the two sets of extracted data. Discrepancies were resolved by discussion (HJH + [GSK, HK or AG]).

We extracted and summarised trial-level data from tables of the MAs but did not inspect original trial publications (with one exception, cf. Additional file 2, Section 2.3.1). Indications/diagnoses in individual trials were coded according to the International Classification of Diseases, 10th Edition (ICD-10). If more than one diagnosis was listed, the first listed diagnosis was coded. If two trials or trial comparisons were analysed separately in one MA and analysed together in another MA, they were counted as 3 trials or trial comparisons, respectively. If more than one trial report for the same trial was listed, only one trial report was extracted.

### Data items

All outcomes in the following subsections refer to the combined effect estimate with a measure of precision for the primary clinical outcome reported in each MA (henceforth ‘effect estimate’).

#### Primary outcome

Effect estimates for.

1. All included trials in each MA.
2. One analysis with the trial sample restricted to ‘high-quality trials’ according to the following criteria, all of which must be fulfilled:
  trials of higher methodological quality (or lower risk of bias), as stated and defined by the authors of the MA
  based on an assessment of at least three specified components of methodological quality (e.g. concealment of allocation sequence, blinding of outcome assessors)
  maximum one single high-quality category defined for the respective MA

#### Sensitivity analyses

Effect estimates in sensitivity analyses, calculated after restricting the sample based on the methodological quality (risk of bias) of individual trials, as assessed by:

- individual quality (risk of bias) components such as concealment of allocation sequence, double blinding [blinding of participants, study personnel and outcome assessors], risk of outcome reporting bias, peer-reviewed trial publication
- the criterion ‘high-quality trials’ (as in Item 2 above) + one or several additional quality components
- other combination of quality components, grouped by total number of components in the respective analysis: 2–4 or ≥ 5
- cumulative MAs with stepwise removal of trials by risk-of-bias ratings, conceptualised in a hierarchical order by the authors of the respective MA (e.g. ascending numbers in a numeric scale or ‘poor’, ‘fair’, ‘good’)

#### Supplementary analyses addressing meta-bias

Effect estimates in supplementary analyses based on assumed risk of bias across trials (meta-bias):

- Statistical adjustment for possible publication bias/small study bias
- Sensitivity analyses, with restrictions of included trials, based on trial sample size
- Analyses addressing possible outcome reporting bias

#### Combined analyses

Effect estimates in analyses combining features of Sections 'Sensitivity analyses' and 'Supplementary analyses addressing meta-bias' above.

#### Subgroup analyses

With regard to research question 2, five types of trial subgroups in the respective MAs (A.1–5) were examined. The subgroup analyses had four types of results (B.1–4), and they were grouped by the timing of the analysis (C.1–2):

A. Subgroup types
  1. Homoeopathy type (*descriptions from Linde 1997* [6]; further descriptions in Suppl. Table 10)
    a individualised or classical homoeopathy (I-HOM) (*single* *homoeopathic* *remedy selected, based on the total symptom picture of a patient*)
    b clinical homoeopathy (*one or several single remedies administered for standard clinical situations or conventional diagnoses*)
    c complex homoeopathy (*multiple remedies mixed into a standard formula to cover a person’s symptoms and diagnoses*)
    d isopathy (*serial agitated dilutions made from the causative agent in an infectious or toxicological condition*)
    e nonindividualised homoeopathy (NI-HOM) = b + c + d
  2. Homoeopathic potency range: low (< C1 or < D24)/high (≥ C12 or ≥ D24)
  3. Age groups: children, adults, elderly (according to definitions in MA)
  4. Indication: acute or chronic (according to definitions in MA)
  5. Type of outcome extracted from trial
    a binary
    b continuous or rank-ordered
B. Analysis results
  1. Tests for interactions between subgroups
  2. Effect estimates in subgroups
  3. Statistical homogeneity/heterogeneity
  4. Funnel plot symmetry/asymmetry and related statistical tests
C. Timing of subgroup analysis
  1. Prespecified (specified in prepublished protocol OR explicitly stated to be prespecified)
  2. Post hoc OR no information

#### Other variables

Other variables collected from the MAs are listed in Suppl. Table 1.

### Assessment of risk of bias in the included MAs

Risk of bias/methodological quality of the MA was assessed using the ROBIS tool (Risk of Bias in Systematic Reviews) [13], supplemented with items 7, 10 and 16 from the AMSTAR-2 tool (A MeaSurement Tool to Assess systematic Reviews) [14], which are not addressed in ROBIS. Assessments were performed independently by two reviewers (HJH, GSK); discrepancies were resolved by discussion between the reviewers.

The outcome of these assessments was the composite body of reports, comprising.

1. protocol for the MA, if available
2. primary publication of the respective MA
3. additional analyses of the MA, if the authors include first author or last author or corresponding author for item 2.

### Effect measures

Effect estimates of each MA (cf. Section 'Outcomes', above) were reported using the metric reported in the MA (e.g., odds ratio [OR], standardised mean difference [SMD]). Standardised mean differences for homoeopathy vs. placebo were reported with point estimates > 0 indicating a benefit of homoeopathy.

### Synthesis methods

Effect estimates were summarised in table format and classified as follows:

1. ‘Significant, positive effect of homoeopathy beyond placebo’: Effect estimate favouring the homoeopathy group with the 95% confidence interval not crossing the boundary between ‘favouring homoeopathy’ and ‘favouring placebo’, as defined in the respective meta-analysis OR (if 95% confidence interval not reported) *p* value < 0.05
2. ‘No significant difference between homoeopathy and placebo’: The 95% confidence interval for the effect estimate crosses the boundary between ‘favouring homoeopathy’ and ‘favouring placebo’, as defined in the respective meta-analysis OR (if 95% confidence interval not reported) p value ≥ 0.05
3. ‘Significant, negative effect of homoeopathy beyond placebo’: same as 1, except the effect estimate favours the placebo group

If both fixed effects and random effects models had been used for the same analysis, the results from random effects models were used for the data synthesis herein.

### Meta-bias assessment

See Sections 'Supplementary analyses addressing meta-bias' and 'Combined analyses', above.

### Confidence in cumulative evidence/certainty assessment

Confidence in cumulative evidence for the two research questions (Sect. Research questions) was assessed.

- For question 1, the conceptual framework of the Grading of Recommendations Assessment, Development and Evaluation (GRADE) group [20] was used, with a focus on six issues: risk of bias of individual trials [21], inconsistency/heterogeneity [22], risk of publication bias/small study bias [23], imprecision [24], indirectness [25] and occasions for rating up the quality of evidence [26].
- For question 2, results of subgroup and heterogeneity [22] analyses were used.

## Results

### Identification, screening and inclusion of meta-analyses

**Fig. 1:**
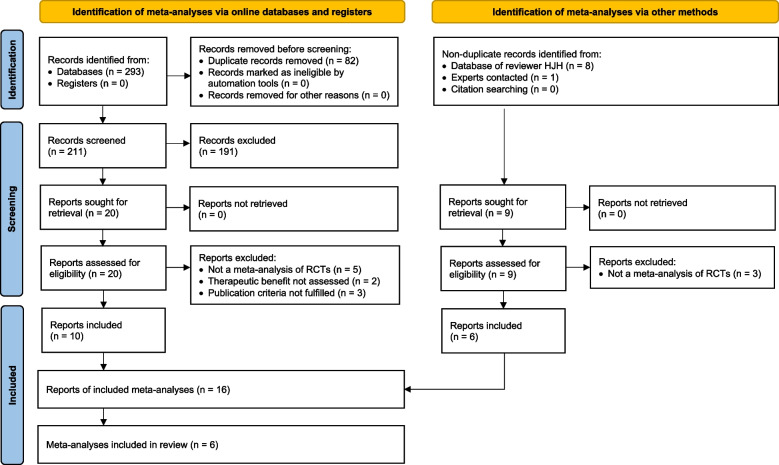
PRISMA 2020 flow diagram for new systematic review which included searches of databases, registers and other sources

From the eight online databases, we identified 293 literature records of potentially eligible meta-analyses (search completed on 14 Dec. 2020). After the removal of 82 duplicates, 211 records were screened, of which 191 were excluded and 20 were further assessed for eligibility. In addition, searches in the database of reviewer HH (20 Jan. 2021 + addition of Gartlehner 2022 on 04 July 2022, cf. Section 'Additional data: Gartlehner 2022') and letters to experts (sent 10 Feb. 2021) yielded a total of 9 nonduplicate records that were also assessed for eligibility. Thus, 29 full-text reports were assessed for eligibility, of which 13 were excluded. Thus, 16 reports of 6 different MAs were included (PRISMA 2020 [27] flow diagram, cf. Fig. 1).

By 30 April 2023, a period of 30 months had passed after the end of the report time frame according to the original eligibility criteria (reports published up to 31 Oct. 2020). We therefore conducted an updated search of reports published in the period from 01 Nov. 2020 to 30 April 2023. We searched databases A–C, E, G–H (Table 2; D was no longer available, and F was omitted for budget reasons, having yielded no nonduplicate records in the primary search) and the database of reviewer HJH. The updated search yielded 13 records, of which 11 were excluded and 2 were assessed for eligibility. Of these, 1 report had already been included on 04 July 2022 (Gartlehner 2022 cf. Section 'Additional data: Gartlehner 2022'), and 1 was excluded (PRISMA 2020 flow diagram for the update in Additional file 4).

A list of the 14 excluded publications (original search: *n* = 13, update *n* = 1) with reasons for exclusions is presented in Suppl. Table 2.

The 16 reports consisted of 6 primary publications of one [6–8, 10, 11] or two [9] MAs, 2 published MA protocols [28, 29], 7 publications of additional analyses [3, 30–34] and 1 error correction [35] (Table 3).

**Table 3:** Overview of included meta-analyses and publications

Primary publication	Protocol	Additional analyses	Error correction
Linde (1997) [6]		Linde (1999) [30]; Sterne (2001) [36]	Linde (1997)-Correction [35]
Linde (1998) [7]			
Cucherat (2000) [8]		Boissel (1996) [31]	
Shang (2005) [9]		Lüdtke (2008) [32]; Rutten (2008) [33]	
Mathie (2014) [10]	Mathie (2014)-Protocol [28]	Mathie (2013) [3]	
Mathie (2017) [11]	Mathie (2017)-Protocol [29]	Mathie (2013) [3]; Gartlehner (2022) [34]	

### Description of meta-analyses

#### Chronological overview

The six MAs were published in the period 1997–2017. The two first (Linde 1997 [6] and 1998 [7]) and the two most recent (Mathie 2014 [10] and 2017 [11]) MAs were MA ‘pairs’, i.e. they were conducted and published by the same first author with overlapping co-authorships. The other two MAs (Cucherat 2000 [8], Shang 2005 [9]) were published by different author groups.

The MA conducted by Linde (1997) [6] was the first MA of placebo-controlled homoeopathy trials for any condition worldwide. The primary publication was followed by a detailed assessment of the relation between study quality (risk of bias) and effect estimates (Linde 1999) [30]. The MA conducted by Linde (1998) [7] was an updated subgroup analysis of Linde (1997) [6], restricted to I-HOM.

The MA conducted by Cucherat (2000) [8] originated from a homoeopathy report prepared for the European Parliament by the Homoeopathic Medicine Research Group (Boissel 1996) [31]. Compared to the Boissel report, the MA conducted by Cucherat [8] had modifications in some analyses. We considered this MA the definitive work, but we also consulted the Boissel report as an additional source of details on the methods and conduct of the MA.

The MA conducted by Shang  [9] was designed as a prospective comparison of two MAs of placebo-controlled trials: one MA of any type of homoeopathic treatment for any disorder and one MA with matched trials on conventional treatment. According to the protocol for the present SR [37], the results of the latter MA were beyond the scope of this SR. However, the authors of the MA conducted by Shang [9] used the results of the MA on conventional treatment to draw inferences about the homoeopathy MA results. We therefore included comparative data on the two MAs (presented in Additional file 2).

The MAs conducted by Mathie (2014, 2017) [10, 11] were part of a comprehensive MA program (Mathie 2013) [3], covering placebo-controlled trials of individualised [10] and nonindividualised  [11] homoeopathy, respectively.

### Methods of the meta-analyses

#### Research objective or hypothesis

The main research objective concerned the efficacy of homoeopathic products vs. placebo in all six MAs: generally stated [7, 8] or in terms of outcome difference between homoeopathy and placebo [6, 10, 11] (full text excerpts in Suppl. Table 3). In the MA conducted by Shang [9], the research hypothesis was further specified: ‘We assumed that the effects observed in placebo-controlled trials of homoeopathy could be explained by a combination of methodological deficiencies and biased reporting’ (Discussion, p.730).

#### Eligibility criteria

##### Design, publication types

In all six MAs, parallel group randomised trials were included, while crossover trials were excluded from four MAs [6, 9–11], included in the MA conducted by Linde (1998) [7] and not mentioned in the MA conducted by Cucherat [8]. Four MAs had no restrictions regarding publication format, while two (Mathie 2014 and 2017) [10, 11] were restricted to peer-reviewed journal articles of at least 500 words (Suppl. Table 4).

##### Patients and indications

Restriction to disease groups as such was not applied in any MA (Suppl. Table 5). Notably, in the MA conducted by Shang [9], the homoeopathy trials were compared to placebo-controlled trials of interventions used in conventional medicine, matched for indication. For 94.0% (*n* = 110/117) of otherwise eligible homoeopathy trials, a trial of conventional medicine for the respective indication could be found, while 7 unmatchable homoeopathy trials were excluded.

##### Interventions, comparators

In the MAs conducted by Mathie (2014 and 2017) [10, 11], the homoeopathic intervention types were restricted as follows: radionically prepared medicines, anthroposophic medicine, homotoxicology, and homoeopathy combined with other (complementary or conventional) treatments were excluded (Suppl. Table 6).

##### Other

In the meta-analysis conducted by Cucherat [8], ‘only trials with a clearly defined primary outcome’ were included (Suppl. Table 7).

#### Literature search and inclusion, data extraction and analysis

For all six MAs, previously published MAs or SRs [38] were consulted. Between 4 [6] and 19 [9] online databases were researched. For all MAs, experts in the field were contacted for information on additional trials; manual searches of reference lists were used in five MAs but not in the MA conducted by Linde (1998) [7], which was largely an update on their previous MA from 1997 (Suppl. Table 8). Screening of titles and abstracts was performed independently by two reviewers in the MA conducted by Linde (1997) [6] and by one reviewer in the MA conducted by Cucherat [8]. The screening approach was not reported in the four other MAs. Full-text assessments were performed independently by two persons in the MA conducted by Linde (1997) [6]; by one person and checked in part by another person in the MA conducted by Cucherat [8]; and by one person in the MA conducted by Linde (1998) [7]. The full text assessment approach was not reported in three MAs.

Data extraction was performed independently by two persons in five MAs and by one person in the MA conducted by Linde (1998 [7]). Risk of bias assessments were performed independently by two persons in three MAs [6, 10, 11] and by one person in the MA conducted by Linde (1998 [7]). The number of persons performing risk of bias assessment was not reported in two MAs. Lists of excluded trials were available in three MAs [9–11]. The reasons for exclusion of trials were provided in all MAs except the one conducted by Linde (1998) [7] (Table 4).

**Table 4:** Quality of trial data handling

Item	Response categories	Linde 1997 [6]	Linde 1998 [7]	Cucherat 2000 [8]	Shang 2005 [9]	Mathie 2014 [10]	Mathie 2017 [11]
Screening of titles and abstracts	1: one person 2-CH and 2-IN: see below^a^ 3: Unclear	2-IN	3	1	3	3	3
Assessment of full text for inclusion		2-IN	1	2-CH	3	3	3
Data extraction		2-IN	1	2-IN	2-IN	2-IN	2-IN
Assessment of study quality/risk of bias		2-IN	1	3	3	2-IN	2-IN
List of excluded trials?	0: No, 1: Yes	0	0	0	1^b^	1	1
Reasons for exclusions of trials provided?	0: No, 1: Yes	1	0	1	1^b^	1	1

^a^2-CH: A performing + B checking parts of A’s results. 2-IN: two persons independently

^b^List published on website of institution of senior author 4 months after publication

All six MAs used one main clinical outcome for each trial or trial comparison. For the MA conducted by Cucherat [8], this was the primary outcome as reported in the trials (cf. Section 'Eligibility criteria', above); for the other MAs, a predefined hierarchical list of criteria for extraction of the main outcome was used (Suppl. Table 9).

#### Protocol

For two MAs (Mathie 2014 and 2017) [10, 11], a prepublished protocol was available; for two MAs (Linde 1997. Cucherat [6, 8]), a protocol was referred to in the publication; and for two MAs (Linde 1998, Shang 2005 [7, 9]), a protocol was not mentioned in the publication, while one single design criterion (outcome extraction in both cases) was explicitly stated as predefined.

#### Risk of bias assessment, heterogeneity, meta-bias

##### High-quality trials

High-quality trials according to our criteria (cf. Section 'Data items' / 'Primary outcome', above) were performed in four MAs [6, 9–11]. The criteria for high-quality trials were described as predefined (Linde 1997) [6] or fully (Mathie 2017) [11] or partially (Mathie 2014) [10] defined in a prepublished protocol. One MA did not mention this aspect (Shang [9]). The criteria for high-quality trials were as follows:

The MA conducted by Linde (1997) [6] used a combination of two score-based instruments:

- Jadad score [39] (range 0–5 points, thereof 0, 1 or 2 points each for items no. 1 and 3 and 0–1 point for item 11 in Table 5): ≥ 3 points
  AND
- Internal validity scale [30] (range 0–7 points, thereof 0, 0.5 or 1 point each for items 1–2, 4–7 and 11 in Table 5): ≥ 5 points

**Table 5:** Criteria for high-quality trials

	Linde (1997) [6]		Shang (2005) [9]	Mathie 2014 [10] and 2017 [11]
Name of quality instruments	Jadad score	Internal Validity scale	[Not stated]	Cochrane risk-of-bias appraisal tool (RoB 1)
Quality components* (1: used; 0: not used)				
1. Generation of allocation sequence adequate	1	1	1	1
2. Allocation concealment adequate	0	1	1	1
3. Double-blinding adequate	1	0	1	0
4. …Blinding of patients^a^	0	1	0	1^a^
5. …Blinding of evaluators	0	1	0	1
6. Baseline comparability adequate	0	1	0	0
7. No selection bias after randomisation	0	1	0	0
8. Completeness of outcome data	0	0	0	1
9. Dropout/withdrawals described	1	0	0	0
10. Intention-to-treat analysis	0	0	1 or 0	0
11. Statistical analysis adequate	0	1	0	0
12. No selective outcome reporting	0	0	0	1
13. No other sources of bias	0	0	0	1
*N different quality components*		8^b^	3 or 4	7

^a^Mathie (2014 and 2017): Blinding of participants and study personnel

^b^*N* quality components in Jadad score + Internal Validity scale, excluding component no. 3 in Jadad, which is redundant with no. 4 and 5 in Internal validity scale

The instruments used in the following MAs consisted of sets of mandatory criteria, all of which were to be fulfilled.

The MAs conducted by Mathie (2014 and 2017) [10, 11] used the Cochrane risk-of-bias tool (RoB, version 2011) [40]: low risk of bias for items 1–2 and 4–5 in Table 5, low risk for two of the three items 8 and 12–13 and low or uncertain risk for one of the latter four items.

In the MA conducted by Shang [9], the number of quality components used was variously described as 3 or 4, corresponding to fulfilment of items (1–3) or (1–3 + 10) in Table 5. Lüdtke [32] interpreted Shang [9] as having used 3 components (Suppl. Table 29). Details in support of either 3 or 4 components are presented in Suppl. Table 11.

The high-quality criteria were based on 8 [6], 7 [10, 11] and either 3 or 4 quality components [9] (Table 5).

#### Risk of bias (methodological quality) otherwise

The total number of methodological quality components assessed in each MA (including components of high-quality criteria as well as other components) ranged from 3 [8] to 10 [6, 7], details in Suppl. Table 12.

Associations between quality components and outcome were analysed with hypothesis testing in four MAs (not in the MA conducted by Linde (1998) [7] and Cucherat [8]).

Cumulative MA with stepwise removal of trials according to increasing quality categories was performed in four MAs using interval-scaled [7, 10, 11] or rank-ordered [8] categories. Of the two other MAs, one [7] had outcome analysis in 4 ranked quality subgroups instead of cumulative MA.

Statistical heterogeneity testing was performed in four MAs (not in the MAs conducted by Linde (1998) [7] and Cucherat [8]); all but one MA [7] included an assessment of publication bias/small study bias (Suppl. Table 14).

Potential conflicts of interest were stated and explained for at least one author in two MAs (Mathie 2014 and 2017) [10, 11]; a statement of no conflicts of interest for any author was included in one MA (Shang) [9], while this issue was not addressed in the three other MAs.

### Trial characteristics

#### Number of trials, trial comparisons and trial reports

For each MA, between 150 and 359 full-text records were assessed for eligibility (data available for four MAs) and between 16 and 119 trials were eligible for SR, including 16–110 trials with extractable data for MA. Altogether, 182 different trials (or in some cases, trial comparisons) reported in 165 different publications or other trial reports were included in the 6 MAs. Of these, *n* = 88 trials were included in 1 MA, 65 trials in 2 MA, 24 trials in 3 MA and 5 trials in 4 MA, with a total of 310 trials or trial comparisons (Suppl. Table 15). All following descriptions refer to these 310 trials.

#### Availability of descriptive data

Summary descriptive data on 12 different trial properties (excluding design, trial quality and results) were presented, ranging from 3 [8] to 9 [7] items per MA (Suppl. Table 16).

All six MAs had at least one table with characteristics of individual trials. A total of 38 different items were presented (or summarily stated as present/absent in all trials), ranging from 8 (Shang [9]) to 33 items (Mathie 2017 [11]) per MA (Suppl. Table 17). The most frequently reported items were as follows:

- first author, number of patients, indication (brief), intervention in homoeopathy group, outcome, summarised rating of methodological quality (presented in *n* = 6 MA)
- indication group, graphical display of effect size with 95% confidence interval (*n* = 5 MA)

#### Descriptive data

The trials were published in the period 1943–2014 (Table 6). The median trial sample size per trial was in the range of 45–97 patients with a minimum sample size of 5–28 and a maximum size of 175–1573 patients. The trials of each MA had been performed in 11–15 countries (data available for four MAs). The countries where each trial was performed was reported in three MAs [7, 10, 11]; the most common countries were the UK (*n* = 18 trials among the three MAs, multiple responses possible), Germany (*n* = 17), USA (*n* = 9) and France and India (both with *n* = 6 trials) (Suppl. Table 18). The most common languages of trial publications were English (range 39–95% of trials), German (5–29%) and French (0–28%) (Table 6).

**Table 6:** Literature searches, characteristics of trials with extractable data for meta-analysis

	Linde (1997) [6]		Linde (1998) [7]		Cucherat (2000) [8]		Shang (2005) [9]		Mathie (2014) [10]		Mathie (2017) [11]	
*N full-text records assessed for eligibility*	No data		No data		150		156		348		359	
*N trials*												
Assessed for eligibility	186		No data		118		No data		No data		No data	
Eligible for systematic review	119		31		17		110		32		75	
Extractable data for meta-analysis	N = 89		N = 18		N = 17		N = 110		N = 22		N = 54	
*Year range for trial publication*	1943–1995		1980–1997		1980–1998		1960–2003		1994–2011		1976–2014	
*Sample size of trials*												
Total	9283		1019		2480		12,879		1335		5739	
Mean	149		38		133		117		33		101	
Median	60		45		97		65		57		64	
Interquartile range	39–120		30–74		53–237		37–104		40–71		39–131	
Total range	5–1270		10–175		28–478		10–1573		23–175		18–478	
*N countries*	13		12		No data		No data		11		15	
*Publication language*	*N*	%	*N*	%	*N*	%	*N*	%	*N*	%	*N*	%
English	35	39.3%	16	88.9%	9	52.9%	57	51.8%	21	95.5%	37	68.5%
French	25	28.1%	0	0.0%	4	23.5%	22	20.0%	0	0.0%	2	3.7%
German	26	29.2%	1	5.6%	4	23.5%	28	25.5%	1	4.5%	13	24.1%
Other	3	3.4%	1	5.6%	0	0.0%	3	2.7%	0	0.0%	2	3.7%
Total	89	100.0%	18	100.0%	17	100.0%	110	100.0%	22	100.0%	54	100.0%

Data on age groups and gender were available in three MAs [7, 10, 11] with a total of 94 trials (multiple responses possible). A total of 14.9% (*n* = 14/94) of all trials included children only, 55.3% (*n* = 52) included adults only and 29.8% (*n* = 28) included both adults and children or unknown. A total of 14.9% (*n* = 14/94) of trials included only females; 2.1% (*n* = 2) of trials included only males; and 83.0% (*n* = 78) of trials included both genders or did not report these data (data on individual MAs in Suppl. Table 19).

Indications for all 310 trials (multiple responses possible) were coded according to ICD-10:

- The most frequent ICD-10 Diagnosis chapters were J00-J99 Diseases of the respiratory system (24.5%, *n* = 76/310), S00-T98 Injury, poisoning and certain other consequences of external causes (11.9%, *n* = 37), K00-K93 Diseases of the digestive system (11.0%, *n* = 34) and M00-M99 Diseases of the musculoskeletal system and connective tissue (8.7%, n = 27) (Suppl. Table 20).
- The most frequent ICD-10 three-digit diagnoses were J30 Vasomotor and allergic rhinitis (7.1%, *n* = 22/310), J11 Influenza, virus not identified (4.8%, *n* = 15), J06 Acute upper respiratory infections of multiple and unspecified sites (4.2%, *n* = 13) and K91 postprocedural disorders of digestive system, not elsewhere classified [postoperative ileus] (4.2%, *n* = 13) (Suppl. Table 21).

#### Interventions, results

The intervention was I-HOM in all trials for 2 MAs [7, 10] and in 0–18% of trials of the four other MAs. In these four MAs, the NI-HOM intervention was clinical homoeopathy in 44–71% of trials, complex homoeopathy in 6–44% (Mathie 2017 [11]: including ‘combination products’) and isopathy in 6–13% (Table 7). The homoeopathic products used were high potencies only (≥ C12 or ≥ D24) in 29–39% of trials.

**Table 7:** Interventions, metric of main outcome, trial results^a^

Meta-analysis	Linde (1997) [6]		Linde (1998) [7]		Cucherat (2000) [8]		Shang (2005) [9]		Mathie (2014) [10]		Mathie (2017) [11]	
*N* trials	85		22		17		110		22		54	
*Homoeopathic intervention*	*N*	%	*N*	%	*N*	%	*N*	%	*N*	%	*N*	%
Individualised	13	14.6%	18	100.0%	3	17.6%	18	16.4%	22	100.0%	0	0.0%
Non-individualised												
Clinical homoeopathy	49	55.1%	0	0.0%	12	70.6%	48	43.6%	0	0.0%	23	42.6%
Complex homoeopathy	20	22.5%	0	0.0%	1	5.9%	35	31.8%	0	0.0%	24	44.4%
Isopathy	7	7.9%	0	0.0%	1	5.9%	8	7.3%	0	0.0%	7	13.0%
Unclear	0	0.0%	0	0.0%	0	0.0%	1	0.9%	0	0.0%	0	0.0%
*High potencies only (≥ C12)?*												
Yes	31	34.8%	7	38.9%	5	29.4%	No data		8	36.4%	21	38.9%
No	58	65.2%	3	16.7%	8	47.1%	No data		9	40.9%	33	61.1%
Unclear	0	0.0%	8	44.4%	4	23.5%	No data		5	22.7%	0	0.0%
*Metric of main outcome*												
Binary	74	83.1%	16	88.9%	No data		No data		16	72.7%	23	42.6%
Continuous or rank-ordered	15	16.9%	2	11.1%	No data		No data		6	27.3%	31	57.4%
*Trial results*												
HOM > PLAC significant	38	42.7%	6	33.3%	11	64.7%	40	36.4%	3	13.6%	15	27.8%
HOM > PLAC not significant	37	41.6%	8	44.4%	3	17.6%	51	46.4%	12	54.5%	26	48.1%
PLAC > HOM not significant	14	15.7%	4	22.2%	3	17.6%	18	16.4%	7	31.8%	13	24.1%
PLAC > HOM significant	0	0.0%	0	0.0%	0	0.0%	1	0.9%	0	0.0%	0	0.0%

^a^Data extracted from tables of individual trials (Shang 2005: in part from summarised data) in publication

The main outcome was binary in 43–89% of trials. The main outcome analysis showed a significant positive effect of homoeopathy compared to placebo in 14–65% (weighted mean 36.5% (*n* = 113 of 310 trials), a nonsignificant superiority of homoeopathy in 18–55% (weighted mean 44.2%), a nonsignificant superiority of placebo in 16–32% (mean 19.0%) and a significant positive effect of placebo compared to homoeopathy in 0–1% (0.3%, *n* = 1 trial) (Table 7).

#### Assessments of bias and heterogeneity

Risk of bias (methodological quality) of trials

##### Overview of methodological quality components

For 10 different methodological quality components, the number of trials fulfilling the respective criterion was assessed in at least two MAs, with a total of 43 analyses (Table 8, components 1–10). Fulfilment rates ranged from 17% (allocation concealment adequate in the MAs conducted by Mathie (2017) [11]) to 100% (8 cases); 44% (*n* = 19/43) of analyses showed a fulfilment rate of ≥ 50%. Weighted mean fulfilment rates for each of the 10 components (multiple responses possible, as trials could be included in more than one MA) ranged from 20% (no funding-related vested interests in the MAs conducted by Mathie (2014) [10] and (2017) [11]) to 89% (publication format = journal article in all six MAs). Three components (journal article, double blinding adequate, no selective outcome reporting) had weighted average fulfilment rates above 75%.

**Table 8:** Risk of bias (methodological quality) of trials: criteria used in ≥ 2 meta-analyses

Quality component	Linde (1997) [6]		Linde (1998) [7]		Cucherat (2000) [8]		Shang (2005) [9]		Mathie (2014) [10]		Mathie (2017) [11]		All MA	
	*N*	*%*	*N*	*%*	*N*	*%*	*N*	*%*	*N*	*%*	*N*	*%*	*N*	*%*
1. Generation of allocation sequence adequate^a^	64	72	15	83	17	100	27	25	11	50	19	35	153	49
2. Allocation concealment adequate	34	38	9	50	17	100	49	45	6	27	9	17	124	40
3. Double-blinding adequate^b^	81	91	18	100	16	94	101	92	15	68	25	46	256	83
4. Dropout handling adequate / ITT analysis / complete outcome data	28	31	6	33	ND	ND	33	30	9	41	20	37	96	33
5. No selective outcome reporting	ND	ND	ND	ND	ND	ND	ND	ND	19	86	40	74	59	78
6. Primary outcome measure stated	21	24	7	39	17	100	ND	ND	ND	ND	ND	ND	45	36
7. Journal article	76	85	16	89	15	88	94	85	22	100	54	100	277	89
8. Peer-reviewed^c^ journal article	23	26	10	56	ND	ND	45	41	22	100	54	100	154	53
9. No funding-related vested interest	ND	ND	ND	ND	ND	ND	ND	ND	4	18	11	20	15	20
10. No other risk of bias	ND	ND	ND	ND	ND	ND	ND	ND	13	59	26	48	39	51
High-quality trials	26	29	ND	ND	ND	ND	21	19	3	14	3	6	53	19
Total trials	89	100	18	100	17	100	110	100	22	100	54	100	310	100

*MA* meta-analyses*, ITT* intention to treat, *ND* no data

^a^Linde (1999) [30]: explicitly randomised

^b^Mathie (2014 and 2017) [10, 11]: ‘Blinding of participants and study personnel’ and ‘Blinding of evaluators’ were assessed separately

^c^Linde (1997) [6] and Shang [9] : Medline-indexed

##### Outcome reporting bias

In the MA conducted by Linde (1997) [6], 23.6% (*n* = 21/89) of trials had a predefined primary outcome (effect estimate after sample restriction to these trials reported in Suppl. Table 28). In the MA conducted by Cucherat [8], only trials with one single ‘clearly defined’ primary outcome were eligible.

In the MAs conducted by Mathie (2014 and 2017) [10, 11], the risk of outcome reporting bias was assessed in Domain V of the Cochrane RoB tool by comparison of the results section with the protocol or, if no protocol was available, with the methods section of publications. In the MA conducted by Mathie (2014) [10], freedom from risk of outcome reporting bias was rated as ‘yes’ in 86.4% (*n* = 19/22) of trials in the MA, ‘uncertain’ in 4.5% (*n* = 1) and ‘no’ in 9.1% (*n* = 2). In the MA conducted by Mathie (2017) [11], the corresponding ratings were ‘yes’ in 74.1% (*n* = 40/54) of the trials in the MA, ‘uncertain’ in 9.3% (*n* = 5) and ‘no’ in 16.7% (n = 9) (Table 8, component no. 5). Effect estimates for the 19 and 40 ‘yes’-rated trials, respectively, were not published.

##### High-quality trials

The proportion of high-quality trials ranged from 6% (*n* = 3/54) of trials analysed by Mathie (2017) [11] to 29% (*n* = 26/89) of trials analysed by Linde (1997) [6] (Table 8). Notably, the criteria for ‘high quality’ differed widely among the MAs:

- High quality (named ‘reliable evidence’) in the MAs conducted by Mathie (2014 and 2017) [10, 11] approximately corresponds to an internal validity scale of 6.5 points or higher in the MA conducted by Linde (1997) [6], which was fulfilled by 8% (*n* = 7/89) trials in the MA conducted by Linde (1997) [6], while 29% fulfilled the high-quality criteria of the authors for Linde (1997) [6].
- If the high-quality criteria in the MAs conducted by Mathie (2014 and 2017) [10, 11] had been restricted to the quality components 1–3 in Table 8 (corresponding to the 3-component model in Shang), the proportion of high-quality trials had been 23% instead of 14% of trials in the MA conducted by Mathie (2014) [10] and 11% instead of 6% in the MA conducted by Mathie (2017) [11]. When applying the same criteria to the MA conducted by Cucherat [8] (which did not have a ‘high-quality trial’ assessment as defined in this SR), they would be fulfilled for 94% of trials.

For the three MAs using a set of mandatory criteria for ‘high-quality’ (Shang with 3 or 4 criteria; Mathie (2014) [10] and (2017) [11] with 7 criteria each), methodological quality was compared with the quality of other trials, assessed according to identical criteria:

- Shang [9] included such a comparison: Among 110 HOM and 110 CON trials, matched for diagnosis and outcome type, the proportion of high-quality trials was significantly higher among HOM trials (19.1%, *n* = 21/110) than for CON trials (8.2%, *n* = 9/110), (*p* = 0.0294) (Additional file 2).
- Mathie [10, 11] used the Cochrane RoB tool (2011 version) with 6 standardised criteria and 1 nonstandardised item ‘other sources of bias’, which was omitted from the subsequent RoB version 2 [41]. In an evaluation of this instrument, the methodological quality of randomised trials in 100 Cochrane SRs and 18 non-Cochrane SRs published at the end of 2014 was summarised using the 6 standardised criteria. The two SRs conducted by Mathie ([10, 11], including trials eligible for SR but not for MA) and the Cochrane SRs had similar proportions of randomised trials rated as having low (A: 3–6%), uncertain (B: 33–38%) and high (C: 59–61%) risk of bias, respectively, while the non-Cochrane SRs had comparatively more trials with uncertain risk (53%) and fewer trials with high risk (41%) [42] (Table 9).

**Table 9:** Risk of bias of trials of systematic reviews, evaluated with the Cochrane RoB tool (2011), domains I, II, IIIa, IIIb, IV and V

	Mathie (2014) [10]		Mathie (2017) [11]		Cochrane reviews		Non-Cochrane reviews	
*N* systematic reviews	1		1		100		18	
*N* trials	32		75		1242		424	
*Risk of bias of trials*	*N*	Percent	*N*	Percent	*N*	Percent	*N*	Percent
A. Low risk	1	3.1%	3	4.0%	74	6.0%	25	5.9%
B. Uncertain risk	12	37.5%	27	36.0%	407	32.8%	226	53.3%
C. High risk	19	59.4%	45	60.0%	761	61.3%	173	40.8%
Total	32	100.0%	75	100.0%	1242	100.0%	424	100.0%

#### Heterogeneity

##### Heterogeneity in the full sample

Significant statistical heterogeneity across trials was found in 3 MAs [6, 9, 11, 30] and was not found in 1 MA (Mathie 2014) [10], while heterogeneity was not assessed in 2 MAs [7, 8] (Suppl. Table 23). Notably, in the MA conducted by Cucherat [8], the likelihood of statistical heterogeneity because of clinical heterogeneity was stated as a major reason for choosing *p* value combination instead of meta-analytic effect estimation.

##### Heterogeneity after sample restriction or ‘trim-and-fill’

In the MA conducted by Linde (1997/1999) [6, 30], heterogeneity was *τ*-squared 0.43 in the full sample (*n* = 89 trials). After sample restriction to trials with higher methodological quality, heterogeneity was reduced in 6 of 7 univariate analyses, with *τ*-squared ranging from 0.31 for double-blind trials (*n* = 81) to 0.41 for explicitly randomised trials (*n* = 64). In one multivariate analysis, heterogeneity was reduced to *τ*-squared = 0.28 for explicitly randomised trials (Suppl. Table 23).

In the MA conducted by Mathie (2017) [11], heterogeneity (I-squared 65%) was not reduced after the ‘trim-and-fill’ procedure for funnel plot asymmetry (FPA, I-squared 79%).

#### Nonreporting bias, small study bias

##### Unavailable trials

Extensive searches for potentially eligible trials were performed for five MAs (not Linde 1998) [7], and unpublished trials were eligible for three MAs [6, 8, 9] but not for the two MAs conducted by Mathie [10, 11].

Data on unavailable trials were reported for three MAs:

- Linde (1997) [6]: The authors assumed that 15–30 unpublished trials that they could not obtain might exist, but did not present any quantitative findings supporting this assumption.
- Cucherat [8]: The authors identified 1 unpublished trial, for which data were protected by industrial property protection laws and hence unavailable.
- Shang [9]: The authors reported 9 unavailable trial reports, thereof 5 journal articles in English (*n* = 2) and Spanish (*n* = 3) language, respectively, and 4 conference proceedings in English language. Of these nine reports, one journal article had been misclassified, as it was actually a case of multiple publication (Straumsheim 1997, included in the MA conducted by Shang [9] as homoeopathy trial No. 87), three journal articles were listed in Mathie (2013) [3] as placebo-controlled trials but not eligible for the MAs conducted by Mathie (2014) [10] (*n* = 2) and Mathie (2017) [11] (*n* = 1), respectively, because they had not been published in a peer-review journal. One conference proceeding (Lara-Marquez 1997) was included in the SR performed by Linde (1998) [7] but not in the respective MA, as it was only available as an abstract (Suppl. Table 24).

##### Unidentified trials

Mathie (2013) [3] identified the following:

- 25 trial reports (2 peer-reviewed, 23 not peer-reviewed) potentially eligible for inclusion in the MA conducted by Linde (1997) [6] but not listed therein,
- 41 trial reports (14 peer-reviewed, 27 not peer-reviewed) potentially eligible for the MA conducted by Shang [9] but not listed therein.

##### Funnel plot, full sample

Funnel plot inspection was performed in four MAs. Funnel plots were constructed by plotting the effect estimate for each trial—expressed as the log odds ratio [6, 9, 10] or standardised mean difference (Mathie 2017 [11])—against the standard error. In three MAs [6, 9, 11], FPA was found, with trials with higher standard error having larger effects. In one MA (Mathie 2014 [10]), the funnel plot was symmetric. Egger’s test was significant in the first three MAs but not in the MA conducted by Mathie (2014) [10] (Suppl. Table 25).

Trim-and-fill tests were performed in three MAs [6, 8, 11]. Random effects and nonparametric selection models to assess possible missing trials were used in the MA conducted by Linde (1997) [6]. Under different conditions, the number of fictive additional trials with zero effect required to change results from a significant to a nonsignificant superiority of homoeopathy ranged from 11 (Mathie (2017) [11]) to 4511 (Linde (1997) [6], fixed effects model) (Suppl. Table 26).

##### Funnel plot, trials with higher quality

Sterne (2001) [36] constructed a funnel plot of *n* = 34 trials with ‘adequate concealment’ + ‘double-blinding’ from the MA conducted by Linde (1997) [6] (not the *n* = 26 high-quality trials according to Linde (1997) [6]). On inspection, FPA was found, and the corresponding tests were significant (rank correlation: *p* = 0.014; regression: *p* < 0.001).

Lüdtke (2008) [32] constructed a funnel plot of the 21 high-quality trials analysed by Shang [9] by plotting the log odds ratio against the standard error. The plot showed a cluster of 18 largely symmetric trials and 3 extreme outliers, with 2 strongly favouring homoeopathy and 1 strongly favouring placebo. Egger’s test showed a large but not significant FPA (asymmetry coefficient 0.40, *p* = 0.17); this was also the case for the 8 largest high-quality trials (1.15, *p* = 0.94, funnel plot not shown) [32] (Suppl. Table 25).

#### Associations between methodological quality and effect estimates

Associations between methodological quality or other subgroups and effect estimates were analysed in 4 MAs (Linde 1997 [6], Shang [9], Mathie 2014 [10] and 2017 [11], Suppl. Table 27).

Linde (1997 [6] and 1999 [30]): The authors analysed uni- and multivariate associations between four single quality components and the effect estimate and found significant associations for ‘double blinding’ (uni- and multivariate) and ‘explicitly randomised’ (multivariate) but not for ‘adequate concealment of random allocation’ nor ‘complete follow-up’ (neither uni- nor multivariate). Univariate analyses showed significant associations between three composite quality measures (A: Jadad scale > 2; B: Internal validity score > 4.5; C: A and B) and effect estimate. On the other hand, scatter plots of the Jadad scale and internal validity score against odds ratios showed no clear linear relationships (Suppl. Table 27).

Linde (1997) [6] / Sterne [36]: The authors analysed uni- and multivariate associations between ‘English language publication’ and ‘Medline-indexed publication’, respectively, and effect estimates: two of four analyses showed significant associations (‘English language’, univariate + ‘Medline-indexed’, multivariate Suppl. Table 27).

Shang [9] analysed univariate associations between six single quality components and effect estimates, and significant associations were found for three (‘Medline-indexed’, ‘double-blinding’, ‘adequate generation of allocation sequence’). Likewise, a significant association was found for high-quality trials (Suppl. Table 27). In multivariate analyses, as summarised by the authors ‘the standard error of the log odds ratio (asymmetry coefficient) was the dominant variable. Coefficients of other variables, including study quality, were attenuated and became non-significant’ (Shang [9], pp.929-930).

The MAs conducted by Mathie (2014 [10] and 2017 [11]) revealed no significant associations between ‘publication free of vested interest’ and effect estimates (both MAs, Suppl. Table 27).

### Risk of bias of meta-analyses

#### ROBIS

According to our ROBIS [13] assessments, the risk of bias was low in three MAs (Linde 1997, Mathie 2014 & 2017 [6, 10, 11]) and high in three MAs (Linde 1998, Cucherat, Shang [7–9]) (Table 10). ROBIS assessments of each MA with our comments on individual items are presented in Additional file 1.

**Table 10:** Risk of bias of meta-analyses: ROBIS assessments of individual items, domains and overall risk

Domains, signalling questions	Linde (1997) [6]	Linde (1998) [7]	Cucherat (2000) [8]	Shang (2005) [9]	Mathie (2014) [10]	Mathie (2017) [11]
*1. Study eligibility criteria*						
1.1 Did the review adhere to predefined objectives and eligibility criteria? (protocol)	Probably Yes	Probably No	Probably Yes	Probably No	Yes	Yes
1.2 Were the eligibility criteria appropriate for the review question?	Probably Yes	Probably No	Probably No	Probably Yes	Yes	Yes
1.3 Were eligibility criteria unambiguous?	Probably Yes	Probably Yes	Yes	No	Yes	Yes
1.4 Were all restrictions in eligibility criteria based on study characteristics appropriate?	Yes	Yes	Probably No	No	Yes	Yes
1.5 Were any restrictions in eligibility criteria based on sources of information appropriate?	Yes	Yes	Yes	Yes	Probably Yes	Probably Yes
1.6 Concerns? (low / high / unclear)	Low	High	High	High	Low	Low
*2. Identification and selection of studies*						
2.1 Did the search include an appropriate range of databases/electronic sources for published and unpublished reports?	Yes	Probably Yes	Yes	Yes	Yes	Yes
2.2 Were methods additional to database searching used to identify relevant reports?	Yes	Yes	Yes	Yes	Yes	Yes
2.3 Were the terms and structure of the search strategy likely to retrieve as many eligible studies as possible?	Probably No	Probably Yes	No Information	Probably No	Yes	Probably Yes
2.4 Were restrictions based on date, publication format, or language appropriate?	Yes	Yes	Yes	Yes	Yes	Yes
2.5 Were efforts made to minimise error in selection of studies?	Yes	No	Probably No	Probably No	Probably No	Probably No
2.6 Concerns? (low / high / unclear)	Unclear	High	Unclear	High	Low	Low
*3. Data collection and study appraisal*						
3.1 Were efforts made to minimise error in data collection?	Yes	No	Yes	Yes	Yes	Yes
3.2 Were sufficient study characteristics available for both review authors and readers to be able to interpret the results?	Yes	Yes	Probably Yes	No	Yes	Yes
3.3 Were all relevant study results collected for use in the synthesis?	Yes	Yes	Yes	Yes	Yes	Yes
3.4 Was risk of bias (or methodological quality) formally assessed using appropriate criteria?	Probably Yes	Probably Yes	Probably Yes	Probably Yes	Yes	Yes
3.5 Were efforts made to minimise error in risk of bias assessment?	Yes	No	Probably No	Probably No	Probably Yes	Probably Yes
3.6 Concerns? (low / high / unclear)	Low	Unclear	Unclear	High	Low	Low
*4. Synthesis and findings*						
4.1 Did the synthesis include all studies that it should?	Yes	Probably Yes	Probably Yes	No	Yes	Probably Yes
4.2 Were all predefined analyses reported or departures explained?	Probably Yes	Probably No	Probably Yes	No	Probably Yes	Yes
4.3 Was the synthesis appropriate, given the nature and similarity in the research questions, study designs, and outcomes across included studies?	Yes	Probably No	Probably Yes	No	Yes	Yes
4.4 Was between-study variation (heterogeneity) minimal or addressed in the synthesis?	Yes	No	Yes	Probably Yes	Yes	Yes
4.5 Were the findings robust, for example, as demonstrated through funnel plot or sensitivity analyses?	Yes	No	Probably Yes	No Information	Yes	No
4.6 Were biases in primary studies minimal or addressed in the synthesis?	Yes	Yes	Probably No	No	Yes	Yes
Concerns? (low / high / unclear)	Low	High	Unclear	High	Low	Low
*Risk of bias in the review*						
A. Did the interpretation of findings address all of the concerns identified in Domains 1 to 4?	Yes	Probably No	Probably Yes	No	Yes	Yes
B. Was the relevance of identified studies to the review's research question appropriately considered?	Yes	Yes	Probably No	Probably No	Yes	Probably Yes
C. Did the reviewers avoid emphasising results on the basis of their statistical significance?	Yes	Yes	Yes	No	Yes	Yes
Risk of bias in the review (low / high / unclear)	Low	High	High	High	Low	Low

#### AMSTAR

AMSTAR [14] items 7 (list of excluded studies), 10 (funding sources for included studies) and 16 (conflict of interest of review authors) received the poorest ratings possible (0) for the first three MAs (Linde 1997 & 1998, Cucherat [6–8]) and the best ratings possible (1 or 2) in the most recent MAs (Mathie 2014 [10] and 2017 [11]). The MA conducted by Shang [9] had two ‘0’ ratings and one ‘1’ (0–2 possible) (Table 11).

**Table 11:** Risk of bias of meta-analyses: AMSTAR items 7, 10, 16

Domain	Linde (1997) [6]	Linde (1998) [7]	Cucherat (2000) [8]	Shang (2005) [9]	Mathie (2014) [10]	Mathie (2017) [11]
7. Did the review authors provide a list of excluded studies and justify the exclusions? 0: No, 1: Partial Yes (provided a list of all potentially relevant studies that were read in full-text form but excluded from the review). 2 = Yes (1 + justified the exclusion from the review of each potentially relevant study)	0	0	0	0	2	2
10. Did the review authors report on the sources of funding for the studies included in the review? 0: No. 1: Yes, they reported on the sources of funding for individual studies included in the review OR the reviewers looked for this information but it was not reported by study authors	0	0	0	0	1	1
16. Did the review authors report any potential sources of conflict of interest, including any funding they received for conducting the review? 0: No. 1: The authors reported no competing interests. 2: The authors described their funding sources and how they managed potential conflicts of interest	0	0	0	1	2	2

### Primary outcome of this systematic review

#### All trials with extractable data for meta-analysis

Effect estimates—or for the MA conducted by Cucherat [8]: combined *p* values—for all trials with extractable data were reported in five MAs (not from Shang [9]). All analyses showed a significant positive effect of homoeopathy compared to placebo (Table 12).

#### Sample restriction to high-quality trials

Effect estimates for high-quality trials Data items / Primary outcome were available for four MAs (not for the MAs conducted by Linde (1998) [7] and Cucherat [8]). Three MAs (Linde 1997, Shang/Lüdtke, Mathie 2014 [6, 9, 10, 32]) showed a significant positive effect of homoeopathy compared to placebo, and one MA (Mathie 2017) [11] showed no significant difference between homoeopathy and placebo (Table 12).

**Table 12:** Primary outcomes of systematic review: effect estimates for all trials and for high-quality trials

Meta-analysis	*N* trials	*N* quality components	Effect size			Favours homoeopathy	Significant?
			Statistic	Metric	Estimate (95% confidence intervaI)		
*All trials with extractable data for meta-analysis*							
Linde (1997) [6]	89	0	Random effects	OR	2.45 (2.05–2.93)	> 1	Yes
Linde (1998) [7]	18	0	Fixed effects	RR	1.66 (1.20–2.28)	> 1	Yes
Linde (1998) [7]	18	0	Fixed effects	OR	2.62	> 1	Yes
Cucherat (2000) [8]	17^a^	0^b^	Not applicable		*p* = 0.000036	NA	Yes
Mathie (2014) [10]	22	0^c^	Random effects	OR	1.53 (1.22–1.91)	> 1	Yes
Mathie (2017) [11]	54	0^c^	Random effects	SMD	0.33 (0.21–0.44)	> 0	Yes
*Sample restriction to high-quality trials*							
Linde (1997) [6]	26	7	Random effects	OR	1.66 (1.33–2.08)	> 1	Yes
Linde (1997 and 1999) [6, 30]	26	7	Meta regression	OR	1.72 (1.28–2.31)	> 1	Yes
Shang (2005)/Lüdtke (2008) [32]	21	3	Random effects	OR	0.76 (0.59–0.99)	< 1	Yes
Mathie (2014) [10]	3	7	Random effects	OR	1.98 (1.16–3.38)	> 1	Yes
Mathie (2017) [11]	3	7	Random effects	SMD	0.18 (− 0.09 to + 0.46)	> 0	No

*OR* odds ratio, *RR* rate ratio, *SMD* standardised mean difference

^a^Cucherat [8]: 17 comparisons from 16 trials

^b^An eligibility criterion for Cucherat [8] was ‘trials with [one] clearly defined primary outcome’, which corresponds to a quality component applied in other MA

^c^An eligibility criterion for Mathie (2014 and 2017) was ‘publication format: peer-reviewed journal article of at least 500 words

### Secondary outcomes

#### Sensitivity analyses: Sample restriction to trials fulfilling quality criteria

Sample restriction to trials fulfilling 1 quality criterion

Sensitivity analyses with sample restriction to trials fulfilling 1 quality criterion were reported in four MAs [6, 7, 10, 11], with a total of 12 analyses based on 7 different single quality components (‘explicitly randomised’, ‘adequate concealment of random allocation’, ‘double-blinding stated’, ‘follow-up adequate/complete’, ‘main outcome predefined’, ‘Medline-listed’, ‘free of [funding-related] vested interest’). Of the 12 analyses, 11 showed a significant positive effect of homoeopathy compared to placebo (Suppl. Table 28).

Sample restriction regarding 2–4 quality components

Sensitivity analyses with sample restriction regarding 2–4 quality components were reported in 3 MAs. In the MA conducted by Linde (1997) [6], trials with a Jadad score > 2 had a significant positive effect of homoeopathy. In the MA conducted by Linde (1998) [7], the effect estimate for trials fulfilling 3 criteria (Medline-indexed + double-blind + “no other obvious relevant flaws”) did not differ significantly from placebo. In the MA conducted by Shang [9] and analysed by Lüdtke [32], the effect estimates for high-quality trials (interpreted as based on 3 components) fulfilling one additional criterion (Medline-listed, English language, Intention-to-treat principle, respectively) analysed with random-effects or meta-regression did not differ significantly from placebo (Suppl. Table 29).

Sample restriction regarding ≥ 5 quality components

Sensitivity analyses with sample restriction regarding 5 or more quality components were reported in 3 MAs with one analysis each. In the MA conducted by Linde (1997) [6], trials with an internal validity score > 4.5 (*n* = 7 components) had a significant positive effect of homoeopathy. In the MAs conducted by Mathie (2014 and 2017) [10, 11], high-quality trials and A- and B-rated trials (trials rated as having low or uncertain risk of bias in all seven domains of Cochrane RoB), respectively, both sets in addition rated as free from publication-rated vested interests (*n* = 8 components each) showed no significant effect differences between homoeopathy and placebo (Suppl. Table 29).

Cumulative MA with stepwise removal of trials by risk-of-bias ratings

Cumulative MA with stepwise removal of trials by risk-of-bias ratings was performed in four MAs, including three (Linde 1997/1999, Mathie 2014 and 2017 [6, 7, 10, 11]) using incremental removal according to interval-scaled instruments and one (Cucherat [8]) using a rank-ordered scale. The scales used by Linde (1997/1999 [6, 30]) were additive (sum of score points), while the remaining scales were in part [10, 11] or fully [8] hierarchically constructed.

In the MA conducted by Linde (1997/1999) [6, 30], two cumulative MAs were performed: (1) For the Jadad score (range 0–5, 5 points indicating highest possible quality), a significant positive effect of homoeopathy was retained with a score of 5 points (*n* = 10 trials). For the internal validity score (range 1–7, 7.0 points indicating highest possible quality), significant positive effects of homoeopathy were retained up to 6.5 points (*n* = 7 trials), while no significant difference was observed for 7.0 points (*n* = 5 trials) (Suppl. Table 31).

In the MA conducted by Cucherat [8], a cumulative MA was performed using a rank-ordered scale, with step 4 indicating the highest possible quality assessed by the authors. Significant positive effects of homoeopathy were retained up to step 3 (double-blind + dropout rate < 10%, *n* = 9 trials), while no significant difference was observed at step 4 (double-blind + dropout rate < 5%, *n* = 5 trials) (Suppl. Table 33).

In the MAs conducted by Mathie (2013/2014 [10, 28] and Mathie (2017) [11]), one cumulative MA was performed based on the Cochrane RoB tool (2011 version), with 7 items for which the risk of bias was rated as low (A), uncertain (B) or high (C). Trials with 7 × A were rated A, trials with 7x (A or B) were rated as B and trials with ≥ 1 × C were rated as C. In addition to this hierarchical classification, Mathie counted the number of A- and B-rated items for each trial, allowing for a more differentiated assessment.

- In the MA conducted by Mathie (2014) [10], significant positive effects of homoeopathy were retained throughout the range up to high-quality trials (criteria in Sect. 3.2.2.5, *n* = 3 trials) (Suppl. Table 31).
- In the MA conducted by Mathie (2017) [11], significant positive effects of homoeopathy were retained up to two steps below high-quality trials (*n* = 14 trials), while no significant difference was observed at one step below high-quality trials (*n* = 13 trials) (Suppl. Table 32).

#### Supplementary analyses: risk of bias across trials (meta-bias)

Statistical adjustment for possible publication bias or other small trial effects

Statistical adjustment for possible publication bias or small trial bias—without any additional sensitivity analysis—was performed for two MAs (Linde 1997, Mathie 2017 [6, 11]). In both cases, a significant positive effect of homoeopathy was retained after adjustment (Suppl. Table 34).

Sensitivity analyses with sample restriction to trials with a higher sample size

Sample restriction to trials with a higher sample size—without any additional sensitivity analysis—was performed for two MAs (Mathie 2014 and 2017) [10, 11]. In both cases, the sample was restricted to trials with a sample size above the median, and in both cases, a significant positive effect of homoeopathy was retained (Suppl. Table 30).

#### Combined sensitivity analyses

Sample restriction regarding methodological quality + restriction to trials with a higher sample size was performed in two MAs (Shang [9]: high-quality trials + “large” trials; Mathie (2017) [11]: A- and B-rated trials + sample size above the median for all trials). In both cases, no significant difference between homoeopathy and placebo was observed (Suppl. Table 35).

Lüdtke [32] performed a cumulative analysis, varying the cut-off point for ‘large trials’ among the 21 high-quality trials included in the MA conducted by Shang [9]: a significant effect of homoeopathy compared to placebo was observed with a sample restriction to the 20, 19, 18, 16, 15 and 14 largest trials, respectively, while no significant difference was found with a sample restriction to the 17, 13 and 1–12 largest trials, respectively [32].

In the MA conducted by Shang [9], meta-regression analyses of ‘predicted effect in trials as large as the largest trials included in the study’ (without further specification; we assume the authors meant the intercept from the regression of odds ratios on the standard error) showed no significant difference between homoeopathy and placebo (Additional file 2).

### Subgroup analyses

#### Tests for interactions

Subgroup interactions were analysed in 3 MAs (Shang, Mathie 2014 and 2017 [9–11]). No significant associations were found for duration of follow-up, indication type (acute/chronic/prophylaxis) or type of homoeopathy (4 groups) (Suppl. Table 36).

#### Effect estimates

Effect estimates were analysed in a total of 23 subgroups, pertaining to indication (acute or chronic), type of homoeopathy (*n* = 10 subgroups), homoeopathic potency (*n* = 6) and outcome metric in trials (*n* = 5) (Suppl. Table 37). Of these 23 analyses, 21 showed a significant positive effect of homoeopathy, while two showed no significant difference from placebo: potencies < 12C in the MA conducted by Mathie (2014) [10], which was restricted to I-HOM; homoeopathic combination products in the MA conducted by Mathie (2017) [11] (a category only described and evaluated in this MA, cf. Suppl. Table 10). No subgroup analyses were performed on patient age groups.

#### Statistical homogeneity/heterogeneity, funnel plot inspection and related tests

Neither statistical homogeneity/heterogeneity nor funnel plot inspection with related statistical tests were reported in any subgroup as defined in Section 'Methods / Subgroup analyses'. However, withstanding that Mathie (2014) [10] and Mathie (2017) [11] were part of one MA programme, these two MAs can be considered subgroup analyses pertaining to the type of homoeopathy. For I-HOM (Mathie 2014 [10], *n* = 22 trials), neither heterogeneity nor FPA was found. For NI-HOM (Mathie 2017 [11], *n* = 54 trials), significant heterogeneity as well as FPA were found (cf. Section 'Assessments of bias and heterogeneity', above).

#### Timing of subgroup analysis

Of the 23 subgroup analyses, 15 were specified in a prepublished protocol (Mathie 2014 and 2017 [10, 11]), while 8 analyses—albeit from MAs based on predefined protocols—were not explicitly stated to be prespecified (Linde 1997 [6], Cucherat 2000 [8]). Of the 15 former analyses, 14 showed a significant positive effect of homoeopathy, while 1 did not (Mathie 2014 [10], see above).

## Additional data: Shang [9]

Data for the comparison of MAs of placebo-controlled trials of homoeopathic and conventional treatment in Shang [9] are presented in Additional file 2.

### Additional data: Gartlehner [34]

After literature searches and data collection for this SR had been completed, an additional subgroup analysis of the MA conducted by Mathie (2017) [11] was published, which we decided to include, as it concerned an item that had not been analysed for any of the MAs: trial registration (Gartlehner 2022) [34]).

The 54 trials included in the MA conducted by Mathie (2017) [11] were published in the period from 1976 to 2014, and 20 of those trials were published from 2002 to 2014. Of this group, Gartlehner et al. analysed 19 trials, stratified according to clinical trial registration, which had been available at ClinicalTrials.gov since 2000. A random effects MA showed a positive significant effect of homoeopathy compared to placebo in *n* = 6 registered trials (SMD 0.53, 95% CI 0.20–0.87) and no significant difference from placebo in *n*= 13 unregistered trials (SMD 0.14, 95% CI − 0.07 to + 0.35). However, the between-group difference in effect estimates was not significant (meta-regression: SMD 0.39, 95% CI − 0.09 to + 0.87) [34]. It is not clear why trial #A93 of the MA conducted by Mathie (2017 [11], Lewith 2002, listed in Gartlehner [34], Supplement Table 3 as ‘not registered’) was not included in these analyses.

The proportion of registered trials was 100% (*n* = 3/3) among high-quality trials and 19% (*n* = 3/16) among the other trials (Suppl. Table 38).

### Confidence in cumulative evidence

The assessment of confidence in cumulative evidence for research questions 1 and 2 (cf. Section 'Research questions', above) according to the GRADE framework (cf. Section 'Confidence in cumulative evidence/Certainty assessment') is presented in Additional file 3. Conclusions are summarised in the following Sections:

#### Conclusion 1: Positive effect of homoeopathy beyond placebo?

The quality of evidence (high/moderate/low/very low) for significant positive effects of homoeopathy beyond placebo is moderate for ALL-HOM and NI-HOM and high for I-HOM.

If the data sources were restricted to MAs with a low risk of bias [6, 10, 11], the quality of evidence would be changed to high for ALL-HOM and remain high for I-HOM and moderate for NI-HOM.

The available data yield no support for the alternative hypothesis of no outcome difference between homoeopathy and placebo.

#### Conclusion 2: Common effect across different treatments and indications?

#### Different types of homoeopathic treatment

The notion of a common positive effect is

- supported for effects across different homoeopathy types, including different subtypes of NI-HOM,
- supported for effects of I-HOM,
- *not* supported for effects of NI-HOM.

As the MA of NI-HOM (Mathie 2017 [11]) comprised different indications treated with different homoeopathic products, the latter finding suggests that the effects of NI-HOM may differ across different indications and/or different homoeopathic products used. Such effect differences may include significant positive effects of NI-HOM as well as no significant difference between NI-HOM and placebo in different subgroups.

#### Different types of indications

The limited data available support the notion of a common positive effect of homoeopathy for acute as well as chronic indications. The issue of effect differences among different diagnoses or diagnosis groups is outside the scope of this SR.

## Discussion

### Main findings

In this first SR of MAs of placebo-controlled randomised trials of homoeopathy for any disorder in humans, homoeopathy had a significant positive effect compared to placebo for all eligible trials in 5 of 5 evaluable MAs and for high-quality trials in 3 of 4 MAs. Assessed by the GRADE system, the quality of evidence for positive effects (high/moderate/low/very low) was high for I-HOM and moderate for ALL-HOM as well as for NI-HOM. There was no support for the alternative hypothesis of no outcome difference between homoeopathy and placebo.

### Strengths and limitations

#### This systematic review as such

The strengths of this SR include a detailed, prepublished PRISMA-P [12] -compliant protocol with two focused research questions, comprehensive presentation of findings, the use of well-established assessment instruments (ROBIS [13], GRADE [20]) and adherence to standard reporting guidelines (PRISMA 2020 [27]).

The scope of this review had two clear limitations: it was restricted to efficacy in placebo-controlled trials and did not address results for specific indications or indication groups.

We used the GRADE system to assess confidence in the cumulative evidence and found it very helpful. Nonetheless, there are three relevant differences between the GRADE approach and this SR: (1) The GRADE approach is indication- and outcome-specific, while we studied MAs with effect estimates for trials with different indications and outcomes. (2) The GRADE framework is tailored to comparative effectiveness, while we assessed MAs of placebo-controlled trials. (3) The GRADE assessment of confidence in cumulative evidence refers to the magnitude of effects, while our research question concerned the existence of significant effects of homoeopathy beyond placebo (yes/no). Accordingly, our conclusions on confidence in the cumulative evidence may not be directly comparable to those of other SRs in the same research field.

#### The meta-analyses included in the review

According to the ROBIS framework, the risk of bias of the six included MAs was rated as low for Linde (1997) [6], Mathie (2014 [10]) and Mathie (2017 [11]) and high for Linde (1998) [7], Cucherat [8] and Shang [9].

- A particular feature of the MA conducted by Linde (1997/1999 [6, 30]) was the detailed assessment of associations between risk of bias and effect estimates in the second paper. *Low risk of bias.*
- The MA conducted by Linde (1998) [7] was an update on the MA conducted by Linde (1997) [6] but restricted to I-HOM. Compared to the 1997 MA, the 1998 MA had a more descriptive and discursive outlook. Having relied on formal and statistical assessments in the 1997 paper, in 1998, the authors made conscious use of subjective judgement, also for the assessment of the risk of bias. Some of these features are not reflected in the ROBIS framework. *High risk of bias.*
- The MA conducted by Cucherat [8] had two particular design features: Because of the expected heterogeneity, *p* value combination was used instead of effect estimation. While other MAs have used a hierarchical algorithm for the selection of outcomes for MAs, the authors restricted eligibility to trials with a single primary outcome. This led to a substantial loss of information that was unaccounted for in the discussion. *High risk of bias.*
- The MA conducted by Shang [9] had an additional comparison between placebo-controlled HOM and CON trials matched for indication and outcome type. Regrettably, the only published effect estimates were those of small subsamples from extreme scenario analyses with severely compromised matching. The authors aimed to demonstrate that effects of homoeopathy could be due to bias. Thereby, they strongly relied on funnel plot-based analyses that had been developed by the senior author [43]. Their approach and the published results were marred by an underlying circular logic, which can be expressed as follows: ‘We assume homoeopathy doesn’t work and found FPA, which may be due to publication bias and small study bias. Admittedly, there are many causes for FPA other than bias, and we know that the funnel plot-based approach cannot prove that results are due to bias (as conceded elsewhere [36]). However, because we assume homoeopathy doesn’t work anyway, we feel confident that the FPA in our MA was due to bias.’ *High risk of bias*.
- The MAs conducted by Mathie (2014 [10] and 2017 [11]) were a predefined MA pair, covering individualised (2014) and nonindividualised (2017) homoeopathy. The problem of persistent heterogeneity and FPA in the earlier MAs could now be clearly localised to the NI-HOM trials, while the I-HOM trials had neither heterogeneity nor FPA. The work also benefited from advances in methodology, guidance and reporting standards. *Low risk of bias for both MAs.*

#### The evidence generated in this systematic review

The evidence generated in this SR is based on 6 MAs, of which the risk of bias was rated as low for 3 and high for 3. If the data were restricted to the 3 MAs with a low risk of bias, the quality of evidence would be rated high for ALL-HOM and I-HOM and moderate for NI-HOM (Additional file 3).

Compared with trials of nonhomoeopathic interventions, which were assessed with identical rating instruments, the methodological quality of the homoeopathy trials in the MAs of this SR was similar for the MAs conducted by Mathie (2014 and 2017 [10, 11]) and higher for the MA conducted by Shang [9]. Significant associations between methodological quality and effect estimates were found in 12 of 24 analyses. After restricting the sample to high-quality trials according to predefined criteria, effect estimates were reduced [6, 11] or increased [10], with 3 of 4 MAs showing significant effects of homoeopathy compared to placebo. When adding a 5^th^ MA (Cucherat [8]) to the assessment and applying the same high-quality criteria as in the 3-component model of Shang [9], 4 of 5 MAs showed significant benefit of homoeopathy.

As assessed by the GRADE system, the quality of evidence for positive effects (high/moderate/low/very low) was high for I-HOM and moderate for NI-HOM and ALL-HOM. In comparison, among 608 Cochrane reviews published from January 2013 to June 2014, the GRADE-assessed quality of evidence for the primary outcome was high in only 13% of reviews, moderate in 31%, low in 32% and very low in 24% [44]. In a randomised sample of Cochrane reviews up until 2021, 90% of 1567 GRADE-assessed interventions were not supported by evidence of high quality [45].

This SR had two limitations. (1) As this was a SR of MAs rather than of individual trials, the trials examined herein were limited to those included in the MAs. Thus, eligible trials published after 2011 and 2014 for I-HOM and NI-HOM, respectively, could not be included. (2) Differential effects of homoeopathy on different indications and patient groups were only assessed for acute and chronic indications and for adults and children, with very limited data available.

### Interpretation of the results in the context of other evidence

According to this SR, homoeopathy can have positive effects beyond placebo on disease in humans. This is in accordance with laboratory experiments showing partially replicable effects of homoeopathically potentised preparations in physico-chemical [46], in vitro [47], plant-based [48, 49] and animal-based [50–52] test systems.

### Implications of the results for practice and policy

In contrast to frequent claims, the available MAs of homoeopathy in placebo-controlled randomised trials for any indication show significant positive effects beyond placebo. Compared to other medical interventions, the quality of evidence for efficacy of homoeopathy was similar or higher than for 90% of interventions across medicine [45]. Accordingly, the efficacy evidence from placebo-controlled randomised trials provides no justification for regulatory or political actions against homoeopathy in health-care systems.

### Recommendations for future research

For I-HOM, an update of the MA conducted by Mathie (2014 [10]) would be warranted to reassess efficacy evidence after inclusion of trials published after 2011. For NI-HOM, the results of the MA conducted by Mathie (2017 [11]) with 54 trials were heterogeneous. Accordingly, future research on the efficacy of NI-HOM should focus on specific nonindividualised forms of homoeopathic therapy or specific interventions therein for specific indications. Recommendations for comparative effectiveness research on homoeopathy are beyond the scope of this review.

### Supplementary Information

**Additional file 1. **Risk of bias of meta-analyses: ROBIS assessments of individual items with comments by the authors of this systematic review.

**Additional file 2. ** Additional data on the comparison of MA of placebo-controlled trials of homoeopathic and conventional treatment, respectively in Shang (2005).

**Additional file 3. ** Confidence in cumulative evidence for research questions 1 and 2, assessed according to the GRADE framework.

**Additional file 4. **Supplementary Tables.

**Additional file 5. **PRISMA 2020 flow diagram for updated systematic reviews which included searches of databases, registers and other sources.

## Data Availability

The complete protocol is permanently available on the website of the institution of the corresponding author: https://www.ifaemm.de/Abstract/PDFs/SMAP-HOM_Protocol_2020_11_25.pdf. All data extracted from the MA publications as well as analyses performed by the authors of this SR are presented in Tables 1, 2, 3, 4, 5, 6, 7, 8, 9, 10, 11, 12 and Additional files 1, 2, 3, 4, 5. Amendments, additional analyses and data Amendments to the protocol from 25 Nov. 2020 are listed and explained in Suppl. Table 39. Additional analyses and data, not described in the protocol, are listed and explained in Suppl. Table 40. Duplicate publications The content of the manuscript has not been published or submitted for publication elsewhere.
