# Supplementary material for: Efficacy of homoeopathic treatment: Systematic review of meta-analyses of randomised placebo-controlled homoeopathy trials for any indication
Source: Syst Rev. 2023 Oct 7;12:191. doi: 10.1186/s13643-023-02313-2 (PMC10559431; doi:10.1186/s13643-023-02313-2)
Supplement: Supplementary file 1 — Additional file 1. Risk of bias of meta-analyses: ROBIS assessments of individual items with comments by the authors of this systematic review. [file 13643_2023_2313_MOESM1_ESM.pdf]

Hamre *et al. Systematic Reviews* (2023), https://doi.org/10.1186/s13643-023-02313-2

Additional file 1. Risk of bias of meta-analyses: ROBIS assessments of individual items with comments by the authors of this systematic review.

Risk of bias of meta-analyses: ROBIS[1] assessments

Linde 1997

Table 1 Risk of bias of the Linde 1997 meta-analysis[2]: ROBIS assessments of individual items with comments by the authors of this systematic review

Domains, Signalling questions	Rating	Comments
1. STUDY ELIGIBILITY CRITERIA		
1.1 Did the review adhere to predefined objectives and eligibility criteria? (protocol)	Probably Yes	An analysis protocol was mentioned but not available. Eligibility criteria were stated as predefined.
1.2 Were the eligibility criteria appropriate for the review question?	Probably Yes	Withstanding the broad objective, eligibility criteria were appropriate, particularly in having no restrictions regarding condition and population. The interventions and outcomes were pragmatically defined in the data extraction section. These items could have been included as additional eligibility criteria.
1.3 Were eligibility criteria unambiguous?	Probably Yes	Eligibility criteria were mostly unambiguous. However, intervention and outcome were only pragmatically defined in the data extraction section and not in an unambiguous way in the eligibility section.
1.4 Were all restrictions in eligibility criteria based on study characteristics appropriate?	Yes	Key restrictions pertain to treatment allocation and blinding, these criteria reflect common methodological knowledge.
1.5 Were any restrictions in eligibility criteria based on sources of information appropriate?	Yes	There were no relevant restrictions based on sources of information.
1.6 Concerns? (low / high / unclear)	Low	There was considerable effort to predefine and adhere to eligibility criteria.
2. IDENTIFICATION AND SELECTION OF STUDIES		
2.1 Did the search include an appropriate range of databases/electronic sources for published and unpublished reports?	Yes	2 generic databases and 6 databases specialised in CAM or homoeopathy trials were searched. The authors also searched private databases and a previous systematic review of controlled clinical trials of homoeopathy for any disorder.[3]
2.2 Were methods additional to database searching used to identify relevant reports?	Yes	Experts and pharmaceutical companies were contacted. Homoeopathic books and conference proceedings were hand searched. Conferences on homoeopathy were attended.
2.3 Were the terms and structure of the search strategy likely to retrieve as many eligible studies as possible?	Probably No	The briefly described search strategy did not include names of manufacturers of homoeopathic products nor homoeopathic substances or products. The database searches might have missed potentially eligible trials of single products in case these were only indexed by the product or substance name without specification as homoeopathic product (although such trials could have been identified by the extensive expert consultation including homoeopathic manufacturers). Admittedly, a search with all potential homoeopathic products may have been unfeasible due to the extremely high number of products. Notably, Matthie 2013 reported 25 trials potentially eligible for Linde 1997 but not listed in Linde 1997. These make up an additional 28.1% (n = 25/89) of the included trials. The number of trials is in the similar range as assumed by Linde 1997 themselves (15-30 trials). The increased availability of full-text

		publications on the internet as well as improved and additional online databases during the 16-year period from Linde 1997 to Mathie 2013 may have contributed to the additional findings of Mathie.
2.4 Were restrictions based on date, publication format, or language appropriate?	Yes	Date: Time period from last month searched to submission of paper is unknown. Corresponding time period until publication of the paper in The Lancet was 23 months, which is reasonable. There were no restrictions regarding publication format or language.
2.5 Were efforts made to minimize error in selection of studies?	Yes	Study selection was performed independently by two reviewers. Prediscussion reliability of the selection process was assessed with the kappa statistic on a random selection of half the trials.
2.6 Concerns? (low / high / unclear)	Unclear	Four signalling questions were rated 'Yes' and one (2.3) 'Probably No'. Because of the additional 25 potentially eligible trials identified by Mathie 2013, without knowledge of eligibility and availability of outcome data extractable for meta-analysis of these trials, there is insufficient information reported to make a judgement on risk of bias for Domain 2.
3. DATA COLLECTION AND STUDY APPRAISAL		
3.1 Were efforts made to minimize error in data collection?	Yes	Data were independently extracted by two reviewers on pretested data extraction sheets.
3.2 Were sufficient study characteristics available for both review authors and readers to be able to interpret the results?	Yes	Descriptive summary data for all trials were available for 9 items. Data on individual trials were available for 9 items.
3.3 Were all relevant study results collected for use in the synthesis?	Yes	For meta-analysis, the authors used a predefined hierarchical selection algorithm to select of one outcome per trial, 1st priority was the outcome used for sample size, which is appropriate. 15 of 89 trials had continuous outcome; standardised mean differences were converted to odds ratios.
3.4 Was risk of bias (or methodological quality) formally assessed using appropriate criteria?	Probably Yes	Jadad score + Internal Validity scale were used, together comprising 9 items. The summary score calculations for the two instruments were, however, based on the unvalidated assumption that quality components have an additive and (for Internal Validity scale) equal impact on risk of bias.
3.5 Were efforts made to minimize error in risk of bias assessment?	Yes	Risk of bias was assessed independently by two reviewers. Interobserver reliability of the quality assessments before discussion was checked with the intraclass correlation coefficient for both scores. The authors used predefined criteria for high-quality trials.
3.6 Concerns? (low / high / unclear)	Low	All signalling questions were rated "Yes" or "Probably Yes". There are low concerns regarding data collection and risk-of-bias assessment.
4. SYNTHESIS AND FINDINGS		
4.1 Did the synthesis include all studies that it should?	Yes	An inclusion criterion was that sufficient data for calculating outcome rates were available. As a quantitative summary of the results was intended, this restriction was legitimate. 89 of 119 trials had adequate information for inclusion. Excluded vs included trials for meta-analyses were comparable for type of homoeopathy, dilution range, country, year, language of publication and. Compared to included trials, excluded trials had lower quality scores, smaller sample sizes, fewer MEDLINE-listed reports and had a similar proportion of trials with positive results (70 vs 67%).
4.2 Were all predefined analyses reported or departures explained?	Probably Yes	An analysis protocol was mentioned but not available. Outcomes for meta-analysis were selected according to a predefined algorithm. Criteria for high-quality trials were also stated as predefined. Descriptions in the method section suggest that further analyses may have been predefined e.g., *"We assumed that publication bias occurred in our data set …to test for its …"*

4.3 Was the synthesis appropriate, given the nature and similarity in the research questions, study designs, and outcomes across included studies?	Yes	The research question was directed at placebo-controlled trials with any indication and clinical outcome. Withstanding this framework, the synthesis was appropriate.
4.4 Was between-study variation (heterogeneity) minimal or addressed in the synthesis?	Yes	The framework described in 4.3 will necessarily lead to clinical heterogeneity with a risk of statistical heterogeneity, as noted by the authors. Accordingly, random-effects models were used in addition to fixed-effects. In the supplementary analyses in Linde 1999, also meta-regression was used. In a total of 8 analyses, heterogeneity was reanalysed after sample restriction to trials of higher methodological quality.
4.5 Were the findings robust, for example, as demonstrated through funnel plot or sensitivity analyses?	Yes	Findings were robust with persisting positive significant effects of homoeopathy compared to placebo after adjustment for possible publication bias, after sample restriction to high-quality-trials, and in cumulative meta-analyses with stepwise removal of trials by increasing quality ratings up to the highest possible rating for Jadad score and the second highest rating for Internal Validity scale.
4.6 Were biases in primary studies minimal or addressed in the synthesis?	Yes	See item 4.5. In addition, Linde 1999 performed extensive analyses of associations between trial quality and trial outcome.
4.7 Concerns? (Low / high / unclear)	Low	All signalling questions were rated “Yes” or “Probably Yes”. The synthesis is unlikely to produce biased results, because any limitations in the data were addressed,
SUMMARY OF CONCERNS		
Domain 1	Low	
Domain 2	Unclear	
Domain 3	Low	
Domain 4	Low	
RISK OF BIAS IN THE REVIEW		
A. Did the interpretation of findings address all of the concerns identified in Domains 1 to 4?	Yes	The concerns were assessed with additional analyses published in an extra paper (Linde 1999) and also extensively discussed (e.g., for concerns in 2.3 and 2.6 with estimation of number of fictive additional trials in order to increase the p-value to insignificance as well as adjustment for possible publication bias, cf. Additional file 3, Section 1.3.6).
B. Was the relevance of identified studies to the review's research question appropriately considered?	Yes	This issue was extensively discussed by the authors.
C. Did the reviewers avoid emphasizing results on the basis of their statistical significance?	Yes	
Risk of bias in the review (Low / high / unclear)	Low	

Linde 1998

Table 2 Risk of bias of the Linde 1998 meta-analysis[4]: ROBIS assessments of individual items with comments by the authors of this systematic review

**Domains, Signalling questions**	**Rating**	**Comments**
1. STUDY ELIGIBILITY CRITERIA		
1.1 Did the review adhere to predefined objectives and eligibility criteria? (protocol)	Probably No	No analysis protocol was mentioned. The eligibility criteria were not stated to be pre-defined. The systematic review and meta-analysis clearly built on the prior meta-analysis of the authors (Linde 1997). However, compared to the eligibility criteria for Linde 1997, those of Linde 1998 differ by including cross-over trials and quasi-randomised trials and allowing other than placebo control-treatments.
1.2 Were the eligibility criteria appropriate for the review question?	Probably No	The review question differs from eligibility criteria on one key issue: Research objective referred to "*randomized clinical trials*", while eligibility criteria comprised *"randomized or quasi-randomized"* studies. – According to table data, 27 of 32 included trials were explicitly randomised, 3 were quasi-randomised and 2 unclear.
1.3 Were eligibility criteria unambiguous?	Probably Yes	Parallel-group design was not mentioned and parallel-group trials were included. As this design is very widespread, its absence among criteria is unimportant. Crossover trials were not mentioned and were included. This design was excluded in four other meta-analyses and its eligibility in Linde 1998 should have been stated. All other criteria were unambiguous.
1.4 Were all restrictions in eligibility criteria based on study characteristics appropriate?	Yes	Key design restrictions ('double blind or randomised / quasi-randomised allocation') were appropriate. Also, three specific criteria for the intervention to be classified as 'individualised homoeopathy' were appropriate.
1.5 Were any restrictions in eligibility criteria based on sources of information appropriate?	Yes	There were no language restrictions nor other relevant restrictions on sources of information.
1.6 Concerns? (Low / high / unclear)	High	There was no protocol, nor were the eligibility criteria stated to be pre-defined. There was a discrepancy between research objective and criteria regarding randomization. A total of 5 of 32 studies that were not or possibly not appropriate for addressing the review question – referring to randomised trials – were included. According to the ROBIS Guidance document, a review with this problem should be classified as having high concerns.
2. IDENTIFICATION AND SELECTION OF STUDIES		
2.1 Did the search include an appropriate range of databases/electronic sources for published and unpublished reports?	Probably Yes	A recent meta-analysis by the authors with very comprehensive search strategy (Linde 1997) was consulted. 3 generic databases (Medline, Embase, Cochrane Trials Registry) and 1 homoeopathy-specific (HomInform) database were searched for new publications.
2.2 Were methods additional to database searching used to identify relevant reports?	Yes	Experts in the field were contacted. Homoeopathic manufacturers were not contacted, but these would very unlikely be relevant for individualised homoeopathy.
2.3 Were the terms and structure of the search strategy likely to retrieve as many eligible studies as possible?	Probably Yes	Search terms were homeop* and homoeop*, which is probably sufficient for individualized homoeopathy. (The omission of names of homoeopathic manufacturers and products is not relevant for individualized homoeopathy, which includes a considerable range of products.) Notably, Matthie 2013 reported an additional 21 trials potentially eligible for Linde 1997 but not listed in Linde 1997. Of these, 5 placebo-controlled trials on individualised homoeopathy published before 1998 (# A166, A170, A171, A173, A177 in Mathie 2013, Web-Appendix 2b) were potentially eligible for Linde 1998. These 5 trials make an additional

		28% to the 18 trials in the 1998 meta-analysis. Notably, the increased availability of full-text publications on the internet as well as improved and additional online trial databases during the 15-year period from Linde 1998 to Mathie 2013 may have contributed to the additional findings of Mathie. It is unclear how many (if any) of these 5 trials would have been technically available and confirmed eligible for Linde 1998.
2.4 Were restrictions based on date, publication format, or language appropriate?	Yes	Date: Time period from last month searched to publication of the paper was short (only 7 months or less). There were no restrictions on publication format nor language.
2.5 Were efforts made to minimize error in selection of studies?	No	Eligibility was assessed by one reviewer only.
2.6 Concerns? Low – High - Unclear	High	Eligibility was assessed by one reviewer only (2.5), the update from Linde 1997 was limited to 4 online databases (2.1) and there is some residual uncertainty about the 5 additional, potentially eligible trials identified in Mathie 2013 (2.3). It seems likely that some eligible studies were missing from this meta-analysis.
3. DATA COLLECTION AND STUDY APPRAISAL		
3.1 Were efforts made to minimize error in data collection?	No	Data extraction was performed by one reviewer only. For the trials included in Linde 1997, the information extracted formerly was used in part.
3.2 Were sufficient study characteristics available for both review authors and readers to be able to interpret the results?	Yes	Descriptive summary data for all trials were available for 5 items. Data on individual trials were available for 21 items.
3.3 Were all relevant study results collected for use in the synthesis?	Yes	For meta-analysis, the authors used a predefined hierarchical selection algorithm to select one outcome per trial, 1st priority was the outcome used for sample size. According to Table 3, this outcome was used for 7 trials, while 2nd and 3rd priorities (global assessments of patient and physician, respectively) were used for 9 of the 11 other trials.
3.4 Was risk of bias (or methodological quality) formally assessed using appropriate criteria?	Probably Yes	Primary bias assessment was only in part criteria-based and involved subjective judgment. In addition, Jadad score + Internal Validity scale were used, together comprising 9 items. The summary score calculations for the two instruments were, however, based on the unvalidated assumption that quality components have an additive and (for Internal Validity scale) equal impact on risk of bias.
3.5 Were efforts made to minimize error in risk of bias assessment?	No	Risk of bias was assessed was performed by one reviewer only. For the trials included in Linde 1997 the information extracted formerly was used in part.
3.6 Concerns? (Low / high / unclear)	Unclear	Data extraction and bias assessment were performed by one reviewer only, with exceptions to data and assessments extracted for Linde 1997. On the other hand, individual trials were thoroughly described including Jadad + Internal Validity score values and the selection of outcome for meta-analysis seems appropriate.
4. SYNTHESIS AND FINDINGS		
4.1 Did the synthesis include all studies that it should?	Probably Yes	18 of the 32 trials had extractable outcomes for meta-analysis. In addition, quantitative outcomes were described for all 32 trials.
4.2 Were all predefined analyses reported or departures explained?	Probably No	No protocol nor use of predefined analysis methods was mentioned.

4.3 Was the synthesis appropriate, given the nature and similarity in the research questions, study designs, and outcomes across included studies?	Probably No	Meta-analysis (also when using random effects model) in the absence of assessment of heterogeneity can be misleading and is not recommended in the ROBIS guidance document.
4.4 Was between-study variation (heterogeneity) minimal or addressed in the synthesis?	No	There was no assessment of statistical heterogeneity.
4.5 Were the findings robust, for example, as demonstrated through funnel plot or sensitivity analyses?	No	There was no assessment of association between study size, as reflected in Standard Error, and effect size (funnel plot). Pooled effect estimates were analysed in four subgroups regarding methodological quality. In the category with highest quality, findings were not robust.
4.6 Were biases in primary studies minimal or addressed in the synthesis?	Yes	Biases in the primary studies were addressed in the synthesis.
4.7 Concerns? (Low / high / unclear)	High	There were problems with items 4.2, 4.3, 4.4 and 4.5.
SUMMARY OF CONCERNS		
Domain 1	High	
Domain 2	High	
Domain 3	Unclear	
Domain 4	High	
RISK OF BIAS IN THE REVIEW		
A. Did the interpretation of findings address all of the concerns identified in Domains 1 to 4?	Probably No	Domain 1: No. Domains 2 and 3: Yes. Domain 4: Partly No.
B. Was the relevance of identified studies to the review's research question appropriately considered?	Yes	
C. Did the reviewers avoid emphasizing results on the basis of their statistical significance?	Yes	
Risk of bias in the review (Low / high / unclear)	High	It is unclear if there was a protocol and if eligibility criteria were predefined. Concerns identified were not (Domain 1) or only in part (Domain 4) addressed by the authors. The latter finding is, according to the ROBIS Guidance document, a criterion for rating the risk of bias in the review as ‘High’.

Cucherat 2000

Table 3 Risk of bias of the Cucherat 2000 meta-analysis[5]: ROBIS assessments of individual items with comments by the authors of this systematic review

**Domains, Signalling questions**	Rating	**Comments**
1. STUDY ELIGIBILITY CRITERIA		
1.1 Did the review adhere to predefined objectives and eligibility criteria? (protocol)	Probably Yes	The authors mentioned "*defined selection criteria described in the protocol for this project*". The protocol was not described as published and was not available for this systematic review. Eligibility criteria for Cucherat 2000 correspond to those in the preceding Boissel 1996 report from the same project, except the restriction to trials with one defined primary outcome (see item 1.2).
1.2 Were the eligibility criteria appropriate for the review question?	Probably No	Inclusion criteria were restricted to "*trials with [one] clearly defined primary outcome*". This criterion led to the exclusion of a very high proportion (78%, n = 92/118) of identified trials – which limits the ability to determine "*whether there is any evidence from randomised controlled trials that homoeopathy is efficacious*" (research objective of the review). In all five other meta-analyses, a predefined hierarchical algorithm was used for outcome selection. A pragmatic and much more informative approach had already been followed in Linde 1997*: "primary outcome measure [defined] by authors"* was used as a quality component (Linde 1997, Table 1) and sample restriction to trials fulfilling this criterion was included as sensitivity analysis (Linde 1997, Table 3, n = 21 of 89 trials).
1.3 Were eligibility criteria unambiguous?	Yes	
1.4 Were all restrictions in eligibility criteria based on study characteristics appropriate?	Probably No	The restriction to "*trials with [one] clearly defined primary outcome"* implied a substantial risk of a loss of information (see Item 1.2).
1.5 Were any restrictions in eligibility criteria based on sources of information appropriate?	Yes	There were no restrictions regarding sources of information.
1.6 Concerns? (Low / high / unclear)	High	There was no explicitly predefined nor pre-published protocol. The exclusion of 78% of identified trials because one single primary outcome was not defined implied a substantial risk of a loss of information. Thus, a criterion in the ROBIS Guidance document for 'High concerns' is fulfilled: "*Studies that would have been important and relevant to answering the review question are likely to have been excluded from the review, …because inappropriate restrictions were imposed*."
2. IDENTIFICATION AND SELECTION OF STUDIES		
2.1 Did the search include an appropriate range of databases/electronic sources for published and unpublished reports?	Yes	The search included 8 electronic databases including one specialised in complementary medicine.
2.2 Were methods additional to database searching used to identify relevant reports?	Yes	The authors searched reference lists of the selected papers and references provided by colleagues. They hand-searched homoeopathic journals and conference abstracts and contacted six pharmaceutical companies.
2.3 Were the terms and structure of the search strategy likely to retrieve as many eligible studies as possible?	No Information	The full search strategy and the exact search terms were not published and were not retrievable for this systematic review.
2.4 Were restrictions based on date, publication format, or language appropriate?	Yes	Date: Time period from last month searched to publication of the Boissel 1998 report, on which Cucherat 2000 is based, was only 6 months. There were no restrictions on publication format nor language.

2.5 Were efforts made to minimize error in selection of studies?	Probably No	The most detailed description of study selection was found in Boissel 1996, Section 11.3.2: One reviewer screened titles and abstracts, retrieved full texts and assessed them regarding 5 criteria ("*clinical trial of homoeopathic medicine, homoeopathic medicine, quantitative results reported, controlled trial, human medicine, control group receiving placebo or no treatment"*). A second reviewer checked the report of the first reviewer with additional assessment of *"randomisation"*. A third reviewer reassessed *"randomisation",* assessed *"primary outcome defined"* plus *"availability and quality of data required for the analysis"* and made the final decision on inclusion. – According to the ROBIS Guidance document, titles and abstracts should be screened independently by at least two reviewers in order to qualify for a 'Yes'-rating.
2.6 Concerns? Low – High - Unclear	Unclear	
3. DATA COLLECTION AND STUDY APPRAISAL		
3.1 Were efforts made to minimize error in data collection?	Yes	Data were extracted independently by two reviewers.
3.2 Were sufficient study characteristics available for both review authors and readers to be able to interpret the results?	Probably Yes	Descriptive summary data for all trials were available for 3 items. Data on individual trials were available for 12 items.
3.3 Were all relevant study results collected for use in the synthesis?	Yes	According to the approach chosen, the p-value of each primary outcome of each trial was combined, using eight different methods for p-value combination.
3.4 Was risk of bias (or methodological quality) formally assessed using appropriate criteria?	Probably Yes	According to Cucherat 2000, p.28, assessment of randomisation during study selection (2.5) included "*adequate concealment of treatment allocation (by a suitable randomisation) method*", implying that all included trials fulfilled the criteria "random treatment allocation" and "adequate allocation concealment". Likewise, all trials had one primary outcome defined in the trial report.<br>Criteria used for assessment of risk of bias of included trials were: A: double blinding, B: dropout <10%, C dropout <5%, resulting in Categories 1 (not A), 2 (A not B), 3 (A + B not C), 4 (A + C). This cumulative meta-analysis does not seem appropriate, as no other relevant methodological were addressed, leading to a relative overemphasizing of absolute dropout rates.<br>On the other hand, the Category 2 includes three components pertaining to the key design elements randomisation and blinding: random allocation + allocation concealment + double-blinding, albeit without separate assessment of 'blinding of patients' and 'blinding of evaluators'. Accordingly, Category 2 in Cucherat 2000 corresponds exactly to the three-item version of high-quality trials in Shang 2005.
3.5 Were efforts made to minimize error in risk of bias assessment?	Probably No?	The assessment of randomisation (two criteria implied) was done by one reviewer and checked by another (cf. 2.5 and 3.2). Dropout rates were extracted independently by two reviewers. There is no explicit statement about the number of reviewers assessing double-blinding.
3.6 Concerns? (Low / high / unclear)	Unclear	Regarding data collection and synthesis there is little concern, while there is some concern about risk of bias assessment.
4. SYNTHESIS AND FINDINGS		
4.1 Did the synthesis include all studies that it should?	Probably Yes	According to Cucherat 2000, 10 of 118 RCTs were excluded because of "*methodological defects*", which is vague. However, the Boissel 1996 report has a similar number of trials excluded because of "*preventive intervention"* (n = 6) and "*homoeopathy vs allopathy*" (n = 6), which are both exclusion criteria for Cucherat 2000. Thus, the data synthesis probably included all studies that it should.

4.2 Were all predefined analyses reported or departures explained?	Probably Yes	In the absence of a protocol a straightforward answer is not possible. However, in lieu of a protocol the earlier Boissel 1996 report on the same project gives relevant information on the four features of the analyses reported in Cucherat 2000:<br>1. The decision to use p-value combination instead of pooled effect estimates is described in Boissel 1996.<br>2. In Boissel 1996, trials with one defined main outcome are called “Class I trials”. For trials with more than one primary outcome or without any specification of primary/secondary outcomes, analyses had been planned but the authors saw no sound way to choose one outcome out of several, and alternative statistical approaches were not judged as suitable (Boissel 1996, p.201). Thus, from the point of view of the authors in 1996, the decision to restrict eligibility to trials with one defined main outcome, as described in Cucherat 2000, seems natural.<br>3. Percentages of trials with/without double-blinding are described (without sensitivity analysis).<br>4. Cut-off points of 5% and 10% for patient dropout are used in Boissel 1996 (with additional sensitivity analyses).<br>In addition, Boissel 1996 reports percentages of trials with/without some other methodological features that are not reported in Cucherat 2000: prior sample size calculation, intention-to-treat analysis, informed consent sought, multicentre trial (no sensitivity analyses).<br>Essentially, the analyses reported in Cucherat 2000 are also reported (or for double-blinding, conceptually present) in the Boissel 1996 report, with only minor deviations.
4.3 Was the synthesis appropriate, given the nature and similarity in the research questions, study designs, and outcomes across included studies?	Probably Yes	The authors refrained from conducting a meta-analysis, arguing that with the expected clinical heterogeneity of patients, indications, homoeopathic treatments, endpoints, “*the assumption of a common underlying treatment effect size used in conventional meta-analytical techniques is clearly not true”*. Instead, they used p-value combination. This approach was unique among the 6 meta-analyses of this review, but the argumentation of the authors cannot be rejected a priori. Their viewpoint is also reflected in the finding of statistical heterogeneity in all meta-analyses except Mathie 2014.<br>Regarding the p-value combination as such, the authors described seven different methods and selected the most conservative for the primary analysis, which is appropriate.
4.4 Was between-study variation (heterogeneity) minimal or addressed in the synthesis?	Yes	Because of expected clinical heterogeneity (cf. 4.3) the authors refrained from performing meta-analysis.
4.5 Were the findings robust, for example, as demonstrated through funnel plot or sensitivity analyses?	Probably Yes	Publication bias was deemed unlikely, as “the addition of 63 fictive non-significant comparisons was needed to obtain a p-value greater than 0.01 and 155 to obtain a P value greater than 0.05”. Findings were also robust after sample restriction to double-blind trials with dropout <10% but not with dropout <5%. See also 4.6.
4.6 Were biases in primary studies minimal or addressed in the synthesis?	Probably No	Bias in primary studies was only to some extent addressed in the synthesis (cf. items 3.4 and 4.5).
4.7 Concerns? (Low / high / unclear)	Unclear	There are some problems with items 4.2 and 4.6, while the other items are adequate.
SUMMARY OF CONCERNS		
Domain 1	High	
Domain 2	Unclear	
Domain 3	Unclear	

Domain 4	Unclear	
RISK OF BIAS IN THE REVIEW		
A. Did the interpretation of findings address all of the concerns identified in Domains 1 to 4?	Probably Yes	The majority of concerns were addressed directly or indirectly in the authors' interpretation.
B. Was the relevance of identified studies to the review's research question appropriately considered?	Probably No	According to the ROBIS guidance document "an important aspect when interpreting the review findings is to consider the relevance (applicability/external validity) of the identified (included) studies to the review's research question." The exclusion of 78% of identified studies because they lacked a "clearly defined primary outcome" (cf. item 1.2) poses a serious threat to the external validity. This problem was not considered.
C. Did the reviewers avoid emphasizing results on the basis of their statistical significance?	Yes	The authors comment on this issue: "The significant combined p-value obtained in the main analysis does not imply that the homoeopathic treatments were efficacious in all the pooled comparisons. This result provides evidence that …more trials had a positive result than would be expected due to chance alone."
Risk of bias in the review (Low / high / unclear)	High	There was substantial loss of information due to restriction to trials with one defined primary outcome (cf. item 1.2). The implication of this problem for external validity of the results was not considered (signalling question B). According to the ROBIS guidance document, a systematic review with this problem ("*the conclusions did not consider the relevance of the included studies to the review question*") should be rated as having a high risk of bias, irrespective of any other merits of the review.

Shang 2005

Table 4 Risk of bias of the Shang 2005 meta-analysis[6]: ROBIS assessments of individual items with comments by the authors of this systematic review

**Domains, Signalling questions**	**Rating**	**Comments**
1. STUDY ELIGIBILITY CRITERIA		
1.1 Did the review adhere to predefined objectives and eligibility criteria? (protocol)	Probably No	A protocol was not mentioned. The eligibility criteria were said to be defined a priori, with one exception: Homoeopathy trials with indications for which no matched trial of conventional medicine could be found were excluded; this was not described as *exclusion* criterion. – These eligibility criteria apply to inclusion into the review. For inclusion into meta-analysis with reported effect estimates, only “large” high-quality trials were permitted. The criteria for high quality and for “large trials” (cf. 1.3) as described in the paper, seem very likely to be defined post-hoc.
1.2 Were the eligibility criteria appropriate for the review question?	Probably Yes	No review question is found in the Methods section. In the Discussion section, the authors report having “assumed that the effects observed in placebo-controlled trials of homoeopathy could be explained by a combination of methodological deficiencies and biased reporting” and “that the same biases could not explain the effects observed in comparable placebo-controlled trials of conventional medicine”. The indirect effect comparison between two meta-analyses with the implicit matching criterion (cf. 1.1) is in keeping with these assumptions. The remaining eligibility criteria were similar to those of the other meta-analyses.
1.3 Were eligibility criteria unambiguous?	No	The matching criteria *“similar disorders + similar outcomes”* are vague and not operationalized. About 3½ months after publication of the paper, the authors published tables with trial characteristics including indications and outcomes on their institution website (no longer available). These tables are grouped by indication groups, but within each group the matched pairs are not shown. Accordingly, the appropriateness of matching procedures cannot be reliably assessed for each pair, only approximately (our reconstruction of matching of indication + outcome is presented in Additional file 2.<br>The very important criterion for high-quality trials is ambiguous, involving either three or four components. Criteria for “large” [high-quality] trials were not defined; for the meta-analysis of conventional medicine trials, procedures were erratic, as the first, second and fourth-to seventh largest high-quality trials were included, omitting the third largest trial.
1.4 Were all restrictions in eligibility criteria based on study characteristics appropriate?	No	The restriction to homoeopathy (HOM) trials with matching trials on conventional medicine (CON) lead to a failure to include all indications for homoeopathy. 14% (n = 7/51) of excluded trials were excluded for this reason. For inclusion into meta-analysis with published effect estimates, the restriction to “large” high-quality trials was inappropriate (cf. Signalling question 4.1).
1.5 Were any restrictions in eligibility criteria based on sources of information appropriate?	Yes	There were no restrictions regarding sources of information.
1.6 Concerns? (Low / high / unclear)	High	A protocol was not mentioned. A possible review question only appears in the Discussion section. Eligibility criteria for (1) matching, (2) high-quality trials and (3) “large” trials are likely to be defined post-hoc (1-3); criteria are imprecise (1) or ambiguous (2+3), and inappropriate (3).

2. IDENTIFICATION AND SELECTION OF STUDIES		
2.1 Did the search include an appropriate range of databases/electronic sources for published and unpublished reports?	Yes	The authors searched 19 databases, including 1 specialised in complementary or alternative medicine and 3 specialised in homoeopathy.
2.2 Were methods additional to database searching used to identify relevant reports?	Yes	The dataset of Linde 1997 was used. Reference lists of relevant papers were searched and experts "*in the specialty*" i.e. homoeopathy experts contacted.
2.3 Were the terms and structure of the search strategy likely to retrieve as many eligible studies as possible?	Probably No	The search strategy was described for Medline not for other databases. The strategy did not include names of manufacturers of homoeopathic products nor homoeopathic products. The database searches might therefore have missed potentially eligible trials of single products in case these were only indexed by the product or substance name without specification as homoeopathic product. There is also no information on homoeopathic manufacturers having been contacted, which could have informed about such trials.<br>Notably, Matthie 2013 reported 41 trials potentially eligible for Shang 2005 but not listed in Shang 2005. These make up an additional 37% (n = 41/110) of the included trials. To some extent, the increased availability of full-text publications on the internet as well as more complete online databases during the 8-year period from Shang 2005 to Mathie 2013 may have contributed to the additional findings of Mathie.
2.4 Were restrictions based on date, publication format, or language appropriate?	Yes	Date: The time period from last month searched to submission of paper is unknown. Corresponding time period until publication of the paper in The Lancet was 29 months, which seems reasonable. There were no restrictions regarding publication format or language.
2.5 Were efforts made to minimize error in selection of studies?	Probably No	5 authors were said to be involved with literature search and selection of trials. However, neither for searches nor for assessment of eligibility was it mentioned if the procedure was done a) once by one author or b) twice in parallel with subsequent comparison or c) once by one author and checked by another author.
2.6 Concerns? Low – High - Unclear	High	Two signalling questions were answered with "Probably No": The database search strategy was incomplete regarding homoeopathic manufacturers and products, homoeopathic manufacturers were not contacted, and there were no documented efforts to minimize error in selection of studies. Matthie 2013 reported an additional 41 trials (37% of the included trials) potentially eligible for Shang 2005 but not listed in Shang 2005. Furthermore, 7 otherwise eligible trials were excluded because no matchable trials of conventional medicine were found. For these reasons, a number of eligible trials are likely to be missing from the review. According to the ROBIS guidance document, this conclusion indicates high concerns.
3. DATA COLLECTION AND STUDY APPRAISAL		
3.1 Were efforts made to minimize error in data collection?	Yes	Data were extracted independently by two reviewers on pretested data extraction sheets, discrepancies were resolved by discussion.
3.2 Were sufficient study characteristics available for both review authors and readers to be able to interpret the results?	No	At the time of publication, the identity of the 8 + 6 trials used for the only published meta-analytic effect estimate was not disclosed to readers, nor were lists of individual trial characteristics or of excluded trials available. The 8 + 6 trials were disclosed in the authors' reply to letters to the editor concerning the paper 3½ months after its publication. At the same time, lists of characteristics of the 2 x 110 trials and of excluded homoeopathy trials were published on authors' institution website (no longer available).<br>Descriptive summary data for all trials were available for 5 items. Data on individual trials were available for 8 items.

3.3 Were all relevant study results collected for use in the synthesis?	Yes	For meta-analysis, the authors used a predefined hierarchical selection algorithm to select of one outcome per trial. 1st priority was the outcome used for sample size, which is appropriate. For trials with continuous outcomes, standardised mean differences were converted to odds ratios.
3.4 Was risk of bias (or methodological quality) formally assessed using appropriate criteria?	Probably Yes	Risk of bias of individual trials was assessed with 5 components: 1) double blind, 2) generation of allocation sequence, 3) concealment of allocation, 4) ITT analysis, 5) Medline-listed journal. Of these five, either #1-3 or #1-4 were used to define high-quality trials (no use of validated risk of bias instrument, but operational criteria for #1-4 were listed). The number of components used to classify high-quality trials is lower than Linde 1997 (n = 9) and Mathie 2014 + 2017 (n = 7 for each) but does include three items pertaining to the key design elements randomisation and blinding (1-3).
3.5 Were efforts made to minimize error in risk of bias assessment?	Probably No	There is no explicit statement about the number of reviewers assessing risk of bias.
3.6 Concerns? (Low / high / unclear)	High	Two signalling questions were answered ‘No’ (3.2) or ‘Probably no’ (3.5). Some bias may have been introduced through the risk of bias assessment processes.
4. SYNTHESIS AND FINDINGS		
4.1 Did the synthesis include all studies that it should?	No	The <u>published</u> data synthesis had no effect estimate for the included n = 110 HOM trials and n = 110 CON trials, nor for the n = 21 HOM and n = 9 CON high-quality trials. Effect estimates were only published for a small extreme-scenario subgroup: the eight largest HOM high-quality trials and the first, second and fourth- to seventh largest CON high-quality trials. The third largest CON high-quality trial (Reuman, #80), was omitted from the data synthesis. An effect estimate for the 21 HOM high-quality trials were published independently of the Shang et al team by Lüdtke 2008.[7]<br>Such a situation is one of three points of concern described in ROBIS Guidance document: “… three situations relating to the studies that have been identified for inclusion in the review… (iii) the reviewers have purposefully excluded the results. …reviewers may make inappropriate decisions to exclude some studies from a synthesis. One potential example would be exclusion of studies deemed to be driving a large between-studies heterogeneity based on statistical considerations alone (although this may be reasonable as a sensitivity analysis).”
4.2 Were all predefined analyses reported or departures explained?	No	No protocol nor the use of predefined analysis methods was mentioned. The authors’ decision to only publish effect estimates for the largest HOM and CON high-quality trials seems very likely to be defined post hoc.
4.3 Was the synthesis appropriate, given the nature and similarity in the research questions, study designs, and outcomes across included studies?	No	The synthesis was focused on “*treatment effects in trials least likely to be affected by bias*” (Shang 2005, Abstract/Background), that is, effects in (A+B): A: the largest trials (in order to eliminate possible publication bias and/or small trial effects, as suspected because of funnel plot asymmetry (FPA) with associated tests for asymmetry coefficient as well as meta-regression analyses), + B: high-quality trials only.<br>1. A major problem concerns A: The authors failed to consider any other causes of funnel plot asymmetry than publication bias and/or small trial effects, while none of the analyses deployed can discern between different causes of FPA, only assess correlation between FPA and trial properties.<br>2. An additional problem concerns B: Following the logic behind A), the meta-regression can inform about relative influence of different trial properties on the effect estimate. In meta-regression analyses, *“asymmetry coefficient was the dominant variable in both groups. Coefficients of other variables, including study quality, were attenuated and became non-significant”* (authors’ description). As study quality (B) was

		outpowered by the asymmetry coefficient in meta-regression (A), a logical consequence would be to implement A but not B in the synthesis, that is, sample restriction to large trials of any quality. But this was not reported, not even as a sensitivity analysis.<br>3. The lack of reporting effect estimates among all trials was commented on in 4.1<br>4. The extreme sample restriction from 2x110 to 8 (7.3%) HOM trials and 6 (5.5%) CON trials lead to a major loss of information, while the matching by indication was severely compromised, with 57% of trials unmatched (Additional file 2, Section 2.3.1).<br>For these four reasons the synthesis was not appropriate.
4.4 Was between-study variation (heterogeneity) minimal or addressed in the synthesis?	Probably Yes	Heterogeneity was present in both trial sets: HOM: Chi^2 = 309, df 109, p<0.0001, I^2 = 65%; CON: Chi^2 = 481, df 109, p<0.0001, I^2 = 77%. However, the authors did not report if or to which extent heterogeneity was reduced by the sample restrictions implemented (Sign. Question 4.3).
4.5 Were the findings robust, for example, as demonstrated through funnel plot or sensitivity analyses?	No Information	Robustness cannot be reliably assessed from the Shang 2005 paper, because the effect estimates for all eligible 110 HOM trials were not published, nor any sensitivity analyses of trial subsets with higher quality. The only published data were two extreme scenarios (1: combined sample restriction to the largest high-quality trials without justification of the cut-off point for large trials; 2: predicted treatment effects in trials as large as the largest trials, both with no significant difference between homoeopathy and placebo) and a subgroup analysis of one indication (acute upper respiratory tract infections) with substantial and significant advantage of homoeopathy over placebo.
4.6 Were biases in primary studies minimal or addressed in the synthesis?	No	Risk of bias was assessed in terms of proportion of trials free from bias risk and associations between risk of bias and outcomes. However, risk of bias was hardly assessed in the synthesis as such: one would have expected to see effect estimates after sample restrictions to trials fulfilling each of the quality criteria implemented in the project: a) double blind; b) generation of allocation sequence, c) concealment of allocation, d) ITT analysis, e) Medline-listed journal f) high-quality (=a+b+c [+d?]). None of these were published in the paper, just the extreme scenario analysis #1 in 4.5.
4.7 Concerns? (Low / high / unclear)	High	Bias may have been introduced by<br>1. Problems with the synthesis [see Sign. Question 4.3],<br>2. Error in the identification of “large” CON trials,<br>3. Post hoc approach to critical analyses,<br>4. Failure to report effect estimates for all trials, all high-quality trials [4.6, f], and trials fulfilling individual quality criteria [4.6, a-e].
SUMMARY OF CONCERNS		
Domain 1	High	
Domain 2	High	
Domain 3	High	
Domain 4	High	
RISK OF BIAS IN THE REVIEW		
A. Did the interpretation of findings address all of the concerns identified in Domains 1 to 4?	No	Most of the concerns and problems of Domains 1 to 4 were not identified by the review authors and were not addressed in the interpretation of the findings.

B. Was the relevance of identified studies to the review's research question appropriately considered?	Probably No	The external validity of the identified studies is not discussed by authors, except for context factors and therapist-patient relationship not being addressed in placebo-controlled trials.
C. Did the reviewers avoid emphasizing results on the basis of their statistical significance?	No	For this signalling question, the ROBIS Guidance document calls for "*a balanced account of all analyses*". The authors chose to report and emphasize only two non-significant effect estimates from extreme scenarios ("largest" high-quality trials with an arbitrary cut-off point + trials as large as the largest trials), failing to report other key results (cf. 4.7, #4). – A subsequent independent re-analysis (Lüdtke 2008[7]) provided one of the missing key results (effect estimate in high-quality trials, showing a significant effect of homoeopathy beyond placebo), and showed that by slightly changing the cut-off point for "large" trials, results could switch from significant to not significant and the other way around. Thus, an emphasis on selectively reported results based on their statistical non-significance cannot be excluded.
Risk of bias in the review (Low / high / unclear)	High	In Phase 2 there were high concerns for all Domains 1-4. In Phase 3, the three signalling questions were answered 'No' (A, C) or 'Probably No' (B).

HOM: Homoeopathy. CON: Conventional medicine

Mathie 2014

Table 5 Risk of bias of the Mathie 2014 meta-analysis[8]: ROBIS assessments of individual items with comments by the authors of this systematic review

**Domains, Signalling questions**	**Rating**	**Comments**
1. STUDY ELIGIBILITY CRITERIA		
1.1 Did the review adhere to predefined objectives and eligibility criteria? (protocol)	Yes	The research objective and eligibility criteria had been defined in a pre-published protocol. Apart from some wording differences, the objective and criteria were identical in protocol and publication. Eligibility criteria were adhered to in the review.
1.2 Were the eligibility criteria appropriate for the review question?	Yes	With regard to the research objective (“To examine the efficacy of the range of homoeopathic medicines that have been used in the context of placebo-controlled trials of individualised homoeopathic treatment” [Protocol]), the eligibility criteria were appropriate, particularly in having no restrictions regarding condition and population. Population/condition, intervention, outcome and control group were all defined and appropriate.
1.3 Were eligibility criteria unambiguous?	Yes	Criteria were well described and unambiguous.
1.4 Were all restrictions in eligibility criteria based on study characteristics appropriate?	Yes	The exclusions of designs (crossover trials and single-blinded trials) and interventions (homoeopathic prophylaxis in healthy individuals, radionically prepared preparations, homoeopathic treatment combined with other therapy) were appropriate.
1.5 Were any restrictions in eligibility criteria based on sources of information appropriate?	Probably Yes	Publication type was restricted to peer-reviewed journal articles of at least 500 words. Peer-review is associated with higher methodological quality, but this restriction might lead to loss of relevant information from trial reports without peer review. However, after a detailed examination of 137 peer-reviewed and 80 non-peer-reviewed placebo-controlled homoeopathy trials (thereof 41 peer-reviewed and 13 non-peer-reviewed trials of individualized homoeopathy), the authors argued: “*the records in the peer-reviewed and non-peer-reviewed domains of the homoeopathy literature have broadly similar characteristics* [suggesting] *that our …focus solely on peer-reviewed publications will not misrepresent the balance of the literature overall” (Mathie 2013, p.24)*. Thus, the restriction seems unproblematic and well justified.
1.6 Concerns? (Low / high / unclear)	Low	No potential concern with the eligibility criteria has been identified. The review objective was clear, eligibility criteria were well defined and justified and had been published a priori.
2. IDENTIFICATION AND SELECTION OF STUDIES		
2.1 Did the search include an appropriate range of databases/electronic sources for published and unpublished reports?	Yes	The authors searched 11 databases, including 1 specialised in complementary and alternative medicine and 2 specialised in homoeopathy.
2.2 Were methods additional to database searching used to identify relevant reports?	Yes	The authors hand-searched reference lists or bibliography sections of previous systematic reviews including Linde 1997 and Shang 2005, original RCT papers, and key text-books. Experts in the field were contacted.
2.3 Were the terms and structure of the search strategy likely to retrieve as many eligible studies as possible?	Yes	Search strings for each database were presented. (The omission of names of homoeopathic manufacturers and products is not relevant for individualized homoeopathy, which includes a considerable range of products.)

2.4 Were restrictions based on date, publication format, or language appropriate?	Yes	Date: the time period from last month searched to submission of paper was only 6 months. Format: the restriction to full-text journal articles was in accordance with the eligibility criteria (cf. 1.5). There were no restrictions regarding publication language.
2.5 Were efforts made to minimize error in selection of studies?	Probably No	There is no information on the number of persons screening titles and abstracts or assessing full text for inclusion. On the other hand, the processes of trial identification and selection are described down to minute details. Identifying trials, the authors identified an additional 25 and 41 trials potentially eligible for Linde 1997 and Shang 2005, respectively, but not listed in these papers. Selecting trials for inclusion, the authors identified studies reported as RCTs (n = 20) or “homoeopathic” (n = 12) which were actually not randomized and/or controlled or “non-homoeopathic”, respectively (Mathie 2014, Additional file 3). Altogether then, substantial efforts appear to have been made. Nonetheless, in the absence of information on the number of reviewers involved, the ROBIS guidance document recommends against a “Yes”-rating.
2.6 Concerns? Low – High - Unclear	Low	Signalling questions 2.1-2.4 were answered “Yes”. Question 2.5 was answered “Probably No” because of lack of information on the number of persons screening titles and abstracts or assessing full text for inclusion. For reasons stated under 2.5 and also with regard to our comments on the other questions, we still think it unlikely that eligible trials were missing in this review.
3. DATA COLLECTION AND STUDY APPRAISAL		
3.1 Were efforts made to minimize error in data collection?	Yes	Data were extracted independently by two reviewers, using a standard recording approach. Discrepancies were resolved by discussion.
3.2 Were sufficient study characteristics available for both review authors and readers to be able to interpret the results?	Yes	Descriptive summary data for all trials were available for 5 items. Data on individual trials were available for 32 items.
3.3 Were all relevant study results collected for use in the synthesis?	Yes	For meta-analysis, the authors used a predefined hierarchical selection algorithm to select of one outcome per trial, based on a WHO classification system. This algorithm does not include the primary outcome as defined by the trial authors or used for sample size calculation. The primary outcome was used as highest priority in the selection algorithms of Linde 1997 & 1998 and Shang 2005 and was the only permissible outcome in Cucherat 2000. This primacy of the primary outcome reflects the inherent logic of clinical trials. For the WHO-based algorithm, highest priority was mortality, which could potentially be problematic e.g., if trials were designed to test effects on symptoms or quality of life in a condition with significant mortality. The subsequent priorities (2: Morbidity, and 3: Health impairment etc.) are unproblematic and may even have advantages over the algorithms of the other meta-analyses. Of the 22 trials included in the data synthesis, only one (4.5%) had mortality as extracted primary outcome. Thus, although objections could be raised against the use of the WHO-based algorithm, its use seems unproblematic here. – For trials with a continuous outcome, standardised mean differences were converted to odds ratios.
3.4 Was risk of bias (or methodological quality) formally assessed using appropriate criteria?	Yes	For descriptive purposes and the classification of high-quality trials (called ‘reliable evidence’), the authors used the Cochrane RoB-1 tool with 7 domains: (I) sequence generation, (II) allocation concealment used to implement the random sequence, (IIIa) blinding of participants and study personnel, (IIIb) blinding of outcome assessors, (IV) incomplete outcome data, (V) selective outcome reporting, (VI) other sources of bias. For each domain, freedom from bias was rated as Yes, Uncertain or No. High-quality trials had ‘Yes’ for the domains (I) to (IIIb) and for at least two of the Domains IV-VI, ‘Uncertain for maximum one of the Domains IV-VI, and ‘No’ for no domain. The primacy of Domains I-IIIb is very appropriate, as these pertain

		to the key design features of randomisation and blinding. – In addition, potential conflicts of interest (called 'vested interests') due to funding sources were assessed.
3.5 Were efforts made to minimize error in risk of bias assessment?	Probably yes	According to the pre-published protocol, "Two assessors will mutually scrutinise and compare their judgments, with discrepancies between them resolved by consensus discussion". In the publication, data extraction was explicitly described as performed independently by two reviewers (item 3.1) but the corresponding procedures for risk of bias rating were not mentioned. The authors having followed the protocol in all other respects; we assume that they did so for this procedure as well.
3.6 Concerns? (Low / high / unclear)	Low	All signalling questions were rated as "Yes" (3.1-4) or "Probably Yes" (3.5). The review processes of data collection and risk of bias assessment are therefore unlikely to have introduced bias.
4. SYNTHESIS AND FINDINGS		
4.1 Did the synthesis include all studies that it should?	Yes	32 trials fulfilled eligibility criteria and were included in the systematic review. Of these, 22 trials had extractable data for meta-analysis and were included in the meta-analysis. The remaining 10 trials without extractable data for meta-analysis were presented at the same level of detail as the 22 trials in the meta-analysis.
4.2 Were all predefined analyses reported or departures explained?	Probably Yes	All published analyses were predefined in the protocol except one: While the framework and categorization of trials into one of 37 possible risk-of-bias tiers was described in the protocol, the additional definition of high-quality trials ('reliable evidence') had not described in the protocol. That said, the criteria for high-quality trials (cf. 3.4) were more comprehensive and conservative than those of Shang 2005 (7 components instead of 3 or 4), represented a clear improvement from the approach of Linde 1997 (the model being hierarchical + additive instead of only additive), and thus did not entail any risk of introducing bias into the synthesis. – The assessment of model validity of included trials was published separately (cf. Risk of bias in the review, signalling question B).
4.3 Was the synthesis appropriate, given the nature and similarity in the research questions, study designs, and outcomes across included studies?	Yes	Because of expected clinical heterogeneity, the authors used random-effect models. Furthermore, appropriate sensitivity analyses and subgroup analyses were performed.
4.4 Was between-study variation (heterogeneity) minimal or addressed in the synthesis?	Yes	Statistical heterogeneity between trials was low (I^2 = 0%, 95% confidence interval 0-40%).
4.5 Were the findings robust, for example, as demonstrated through funnel plot or sensitivity analyses?	Yes	In a sensitivity analysis with sample restriction to high-quality trials, the effect estimate for homoeopathy vs placebo (OR = 1.98, n = 3 trials) was higher than in the full dataset (OR = 1.63, n = 22). The published funnel plot was symmetric, with Egger's test non-significant (p = 0.59).
4.6 Were biases in primary studies minimal or addressed in the synthesis?	Yes	Trials were categorized according to Cochrane RoB-1 (three categories):<br>Rating A = Low risk of bias in all seven domains (n = 0 trials).<br>Rating Bx = Uncertain risk of bias in x domains; low risk of bias in all other domains (n = 12).<br>Rating Cy.x = High risk of bias in y domains; uncertain risk of bias in x domains; low risk of bias in all other domains (n = 20).<br>Using x- and y- RoB-1 numbers within a hierarchical model, cumulative meta-analysis with incremental stepwise removal of trials with higher risk of bias (twelve categories; Mathie 2014, Fig. 4) was performed. Contrary to what might be expected, the effect estimate remained stable or even increased with progressive restriction to trials with comparatively lower risk of bias.

4.7 Concerns? (Low / high / unclear)	Low	5 of 6 signalling questions were rated 'Yes', one (4.2) as 'Probably Yes'. The synthesis is unlikely to produce biased results.
SUMMARY OF CONCERNS		
Domain 1	Low	
Domain 2	Low	
Domain 3	Low	
Domain 4	Low	
RISK OF BIAS IN THE REVIEW		
A. Did the interpretation of findings address all of the concerns identified in Domains 1 to 4?	Yes	There were 4x 'Low concerns' identified in Domains 1 to 4.
B. Was the relevance of identified studies to the review's research question appropriately considered?	Yes	As stated in the protocol, the relevance to the research questioned (called 'model validity') of the 32 included trials was assessed in a separate paper. According to predefined criteria [9], model validity was categorized into three levels: 'acceptable' (n = 19 trials), 'uncertain' (n = 9), 'inadequate' (n = 4) [10].
C. Did the reviewers avoid emphasizing results on the basis of their statistical significance?	Yes	The authors discussed limitations of primary trials and the data synthesis, prompting "*caution in interpreting the findings"* (Abstract).
Risk of bias in the review (Low / high / unclear)	Low	In Phase 2 there were Low concerns for all four domains. In Phase 3, all three signalling questions were answered 'Yes'. The findings of this review are likely to be reliable. No relevant concerns with the review and analysis process were identified. The findings were robust, while limitations of the evidence were clearly pointed out by the authors.

Mathie 2017

Table 6 Risk of bias of the Mathie 2017 meta-analysis[11]: ROBIS assessments of individual items with comments by the authors of this systematic review

Domains, Signalling questions	Rating	Comments
1. STUDY ELIGIBILITY CRITERIA		
1.1 Did the review adhere to predefined objectives and eligibility criteria? (protocol)	Yes	Research objective and eligibility criteria had been defined in a pre-published protocol. Apart from some wording differences, the objective and criteria were identical in protocol and publication. Eligibility criteria were adhered to in the review.
1.2 Were the eligibility criteria appropriate for the review question?	Yes	With regard to the research objective ("To examine the efficacy of the range of homoeopathic medicines that have been used in the context of placebo-controlled trials of non-individualised homoeopathic treatment" [Protocol]), the eligibility criteria were appropriate, particularly in having no restrictions regarding condition and population. Population/indication, intervention, outcome and control group were all defined and appropriate.
1.3 Were eligibility criteria unambiguous?	Yes	Criteria were well described and unambiguous.
1.4 Were all restrictions in eligibility criteria based on study characteristics appropriate?	Yes	The exclusions of designs (crossover trials and single-blinded trials) and interventions (homoeopathic prophylaxis in healthy individuals; radionically prepared preparations, homoeopathic treatment combined with other therapy) were appropriate.
1.5 Were any restrictions in eligibility criteria based on sources of information appropriate?	Probably Yes	Publication type was restricted to peer-reviewed journal articles of at least 500 words. Peer-review is associated with higher methodological quality, but this restriction might lead to loss of relevant information from trial reports without peer review. However, after a detailed examination of 137 peer-reviewed and 80 non-peer-reviewed placebo-controlled homoeopathy trials (thereof 96 peer-reviewed and 67 non-peer-reviewed trials of non-individualized homoeopathy), the authors argue: "*the records in the peer-reviewed and non-peer-reviewed domains of the homoeopathy literature have broadly similar characteristics* [suggesting] *that our …focus solely on peer-reviewed publications will not misrepresent the balance of the literature overall" (Mathie 2013, p.24)*. Thus, the restriction seems unproblematic and well justified.
1.6 Concerns? (Low / high / unclear)	Low	No potential concern with the eligibility criteria has been identified. The review objective was clear, eligibility criteria were well defined and justified and had been published a priori.
2. IDENTIFICATION AND SELECTION OF STUDIES		
2.1 Did the search include an appropriate range of databases/electronic sources for published and unpublished reports?	Yes	The authors searched 11 databases, including 1 specialised in complementary and alternative medicine and 2 specialised in homoeopathy.
2.2 Were methods additional to database searching used to identify relevant reports?	Yes	The authors hand-searched reference lists or bibliography sections of previous systematic reviews including Linde 1997 and Shang 2005, original RCT papers, and key text-books. Experts in the field were contacted.
2.3 Were the terms and structure of the search strategy likely to retrieve as many eligible studies as possible?	Probably Yes	Search strings for each database were presented. The search strategy did not include names of manufacturers of homoeopathic products nor homoeopathic products. The database searches might have missed potentially eligible trials of single products, in case these were only indexed by the product or substance name without specification as homoeopathic product. On the other hand, extensive efforts were

		made in addition to database searches (item 2.2). And empirically, Matthie 2013 reported an additional 25 and 41 trials potentially eligible for Linde 1997 and Shang 2005, respectively but not listed in these two papers. Thus, the likelihood of having missed eligible trials seems low.
2.4 Were restrictions based on date, publication format, or language appropriate?	Yes	Date: the time period from last month searched to submission of paper was 20 months, which is acceptable for a very thorough systematic review with meta-analysis on a complicated topic. Format: the restriction to full-text journal articles is in accordance with the eligibility criteria (cf. 1.5). There were no restrictions regarding publication language.
2.5 Were efforts made to minimize error in selection of studies?	Probably No	There is no information on the number of persons screening titles and abstracts or assessing full text for inclusion. On the other hand, the processes of trial identification and selection are described down to minute details. Identifying trials, the authors identified an additional 25 and 41 trials potentially eligible for Linde 1997 and Shang 2005, respectively, but not listed in these papers. Selecting trials for inclusion, the authors identified studies reported as RCTs (n = 24) or “homoeopathic” (n = 12) which were actually not randomized and/or controlled or “non-homoeopathic”, respectively (Fig. 1). Altogether then, substantial efforts appear to have been made. Nonetheless, in the absence of information on the number of reviewers involved, the ROBIS guidance document recommends against a “Yes”-rating.
2.6 Concerns? Low – High - Unclear	Low	Signalling questions 2.1-2.4 were answered ‘Yes’ (3x) or ‘Probably Yes’ (1x). Question 2.5 was answered ‘Probably No’ because of lack of information on the number of persons screening titles and abstracts or assessing full text for inclusion. For reasons stated under 2.5 and also with regard to our comments on the other questions, we still think it unlikely that eligible trials were missing in this review.
3. DATA COLLECTION AND STUDY APPRAISAL		
3.1 Were efforts made to minimize error in data collection?	Yes	Data were extracted independently by two reviewers, using a standard recording approach. Discrepancies were resolved by discussion.
3.2 Were sufficient study characteristics available for both review authors and readers to be able to interpret the results?	Yes	Descriptive summary data for all trials were available for 6 items. Data on individual trials were available for 33 items.
3.3 Were all relevant study results collected for use in the synthesis?	Yes	For meta-analysis, the authors used a predefined hierarchical selection algorithm to select of one outcome per trial, based on a WHO classification system. This algorithm does not include the primary outcome as defined by the trial authors (or used for sample size calculation). The primary outcome was used as highest priority in the selection algorithms of Linde 1997 & 1998 and Shang 2005, while its availability was a mandatory inclusion criterion in Cucherat 2000. This primacy of the primary outcome reflects the inherent logic of clinical trials. For the WHO-based algorithm, highest priority was mortality, which could potentially be problematic e.g., if trials were designed to test effects on symptoms or quality of life in a condition with significant mortality. However, none of the included trials had mortality as outcome.<br>The subsequent priorities (2: Morbidity, and 3: Health impairment etc.) are unproblematic and may even have advantages over the algorithms of the other meta-analyses.
3.4 Was risk of bias (or methodological quality) formally assessed using appropriate criteria?	Yes	For descriptive purposes and the classification of high-quality trials (called ‘reliable evidence’), the authors used the Cochrane RoB-1 tool with 7 domains: (I) sequence generation, (II) allocation concealment used to implement the random sequence, (IIIa) blinding of participants and study personnel, (IIIb) blinding of outcome assessors, (IV) incomplete outcome data, (V) selective outcome reporting, (VI) other sources of bias. For each domain, freedom from bias was rated as Yes, Uncertain or No. High-quality trials had ‘Yes’ for the domains (I) to (IIIb) and for at least two of the Domains IV-VI, ‘Uncertain for maximum one of the

		Domains IV-VI, and 'No' for no domain. The primacy of Domains I-IIIb is very appropriate, as these pertain to the key design features of randomisation and blinding.<br>In addition, potential conflicts of interest (called 'vested interests') due to funding sources were assessed.
3.5 Were efforts made to minimize error in risk of bias assessment?	Probably Yes	According to the pre-published protocol, "Two assessors will mutually scrutinise and compare their judgments, with discrepancies between them resolved by consensus discussion". In the publication, data extraction was explicitly described as performed independently by two reviewers (item 3.1) but the corresponding procedures for risk of bias rating were not mentioned. The authors having followed the protocol in all other respects; we assume that they did so for this procedure as well.
3.6 Concerns? (Low / high / unclear)	Low	All signalling questions were rated as 'Yes' (3.1-4) or 'Probably Yes' (3.5). The review processes of data collection and risk of bias assessment are unlikely to have introduced bias.
4. SYNTHESIS AND FINDINGS		
4.1 Did the synthesis include all studies that it should?	Probably Yes	75 trials fulfilled eligibility criteria and were included in the systematic review. Of these, 54 trials had extractable data for meta-analysis and were included in the meta-analysis. The remaining 21 trials without extractable data for meta-analysis were presented at the same level of detail as the 54 trials in the meta-analysis.
4.2 Were all predefined analyses reported or departures explained?	Yes	All reported analyses were predefined in pre-published protocol except two:<br>1) The protocol foresaw separate effect estimates for continuous (standardised response mean, SMD) and binary (odds ratios, OR) outcomes, with an additional effect estimate for all trials using OR, as was done in Mathie 2014. Instead SMD was chosen as metric for the effect estimate for all trials. This departure from the protocol is appropriate (as in Mathie 2014, most trials had continuous outcomes) and was explained by the authors.<br>2) In the protocol three subtypes of non-individualised homoeopathy (clinical homoeopathy, complex homoeopathy and isopathy) had been described without predefining their use in any subgroup analysis. Such a subgroup analysis was performed, thereby an additional category 'combination product' was plausibly defined and used. These additional analyses are clearly appropriate, withstanding the research objective, the prior description of the subgroups in the protocol, and the published analyses of the same subgroups (except combination product) in Linde 1997 and Cucherat 2000.
4.3 Was the synthesis appropriate, given the nature and similarity in the research questions, study designs, and outcomes across included studies?	Yes	Because of expected clinical heterogeneity, the authors used random-effect models. Furthermore, appropriate sensitivity analyses and subgroup analyses were performed.
4.4 Was between-study variation (heterogeneity) minimal or addressed in the synthesis?	Yes	Statistical heterogeneity between trials was considerable and significant (I-squared = 65%; tau−squared = 0.1094, p < 0.0001.) The authors also reported heterogeneity after adjustment for possible publication bias with 'trim-and-fill' method (I-squared = 75%).
4.5 Were the findings robust, for example, as demonstrated through funnel plot or sensitivity analyses?	No	The effect estimate for homoeopathy vs placebo was SMD 0.33 (95%-CI 0.21-0.44) for all 54 trials. In a sensitivity analysis with sample restriction to high-quality trials, the effect size was reduced and no longer significant (SMD 0.18, 95%-CI -0.09 to +0.46, n = 3 trials).<br>In addition, the authors performed a cumulative meta-analysis with incremental removal of studies with higher risk of bias: At all steps the effect estimate favoured homoeopathy over placebo. The difference was significant from "all studies" and up till "Retain B3 studies and better", while it was not insignificant from "Retain B2 studies and better" and upwards.

		The published funnel plot was asymmetric on inspection, confirmed by Egger's test (p = 0.002). After adjustment by trim-and-fill, the effect size was reduced but remained significant, compared to placebo: SMD 0.16 (95%-CI 0.02-0.31), p = 0.023.<br>Because of the low number of high-quality trials with a small and not significant effect size, findings were clearly not robust.
4.6 Were biases in primary studies minimal or addressed in the synthesis?	Yes	The biases in primary trials were addressed in the synthesis and discussion section.
4.7 Concerns? (Low / high / unclear)	Low	The synthesis is unlikely to produce biased results. Limitations of individual trials, between-trial heterogeneity and funnel plot asymmetry were all addressed and accounted for.
SUMMARY OF CONCERNS		
Domain 1	Low	
Domain 2	Low	
Domain 3	Low	
Domain 4	Low	
RISK OF BIAS IN THE REVIEW		
A. Did the interpretation of findings address all of the concerns identified in Domains 1 to 4?	Yes	There were 4x 'Low concerns' identified in Domains 1 to 4.
B. Was the relevance of identified studies to the review's research question appropriately considered?	Probably Yes	As stated in the protocol, the relevance to the research questioned (called 'model validity') of the trials was assessed in a separate paper. This analysis was restricted to trials rated "*not at high risk of bias*" i.e. excluding C-rated trials, which is acceptable. According to predefined criteria[9], model validity was categorized into three levels: 'acceptable' (n = 9 trials), 'uncertain' (n = 10), 'inadequate' (n = 7).[12]
C. Did the reviewers avoid emphasizing results on the basis of their statistical significance?	Yes	The analysed effect estimates and other outcomes were predefined. The conclusions reflect both significant and non-significant findings.
Risk of bias in the review (Low / high / unclear)	Low	In Phase 2 there were Low concerns for all four domains. In Phase 3, the signalling questions were answered 'Yes' (A, C) or 'Probably Yes'. The findings of this review are likely to be reliable. No concerns with the review and analysis processes were identified. Limitations of the trials were addressed in an appropriate way and reflected in the overall conclusions.

References

1. Whiting P, Savovic J, Higgins JP, et al. ROBIS: A new tool to assess risk of bias in systematic reviews was developed. *J Clin Epidemiol* 2016; **69**: 225-34 https://doi.org/10.1016/j.jclinepi.2015.06.005.
2. Linde K, Clausius N, Ramirez G, et al. Are the clinical effects of homoeopathy placebo effects? A meta-analysis of placebo-controlled trials. *Lancet* 1997; **350**: 834-43 https://doi.org/10.1016/s0140-6736(97)02293-9.
3. Kleijnen J, Knipschild P, ter Riet G. Clinical trials of homoeopathy. *BMJ* 1991; **302**(6772): 316-23 https://doi.org/10.1136/bmj.302.6772.316.
4. Linde K, Melchart D. Randomized controlled trials of individualized homeopathy: a state-of-the-art review. *J Altern Complement Med* 1998; **4**(4): 371-88 https://doi.org/10.1089/acm.1998.4.371.
5. Cucherat M, Haugh MC, Gooch M, Boissel JP. Evidence of clinical efficacy of homeopathy. A meta-analysis of clinical trials. HMRAG. Homeopathic Medicines Research Advisory Group. *Eur J Clin Pharmacol* 2000; **56**(1): 27-33 https://doi.org/10.1007/s002280050716.
6. Shang A, Huwiler-Muntener K, Nartey L, et al. Are the clinical effects of homoeopathy placebo effects? Comparative study of placebo-controlled trials of homoeopathy and allopathy. *Lancet* 2005; **366**(9487): 726-32 https://doi.org/10.1016/S0140-6736(05)67177-2.
7. Lüdtke R, Rutten AL. The conclusions on the effectiveness of homeopathy highly depend on the set of analyzed trials. *J Clin Epidemiol* 2008; **61**(12): 1197-204 https://doi.org/10.1016/j.jclinepi.2008.06.015.
8. Mathie RT, Lloyd SM, Legg LA, et al. Randomised placebo-controlled trials of individualised homeopathic treatment: systematic review and meta-analysis. *Syst Rev* 2014; **3**: 142 https://doi.org/10.1186/2046-4053-3-142.
9. Mathie RT, Roniger H, van WM, et al. Method for appraising model validity of randomised controlled trials of homeopathic treatment: multi-rater concordance study. *BMC Med Res Methodol* 2012; **12**: 49 https://doi.org/10.1186%2F1471-2288-12-49.
10. Mathie RT, Van Wassenhoven M, Jacobs J, et al. Model validity of randomised placebo-controlled trials of individualised homeopathic treatment. *Homeopathy* 2015; **104**(3): 164-9 https://doi.org/10.1016/j.homp.2015.02.004.
11. Mathie RT, Ramparsad N, Legg LA, et al. Randomised, double-blind, placebo-controlled trials of non-individualised homeopathic treatment: systematic review and meta-analysis. *Syst Rev* 2017; **6**(1): 63 https://doi.org/10.1186/s13643-017-0445-3.
12. Mathie RT, Van Wassenhoven M, Rutten ALB, et al. Model validity of randomised placebo-controlled trials of non-individualised homeopathic treatment. *Homeopathy* 2017; **106**(4): 194-202 https://doi.org/10.1016/j.homp.2017.07.003.
