# Supplementary material for: Efficacy of homoeopathic treatment: Systematic review of meta-analyses of randomised placebo-controlled homoeopathy trials for any indication
Source: Syst Rev. 2023 Oct 7;12:191. doi: 10.1186/s13643-023-02313-2 (PMC10559431; doi:10.1186/s13643-023-02313-2)
Supplement: Supplementary file 2 — Additional file 2. Additional data on the comparison of MA of placebo-controlled trials of homoeopathic and conventional treatment, respectively in Shang (2005). [file 13643_2023_2313_MOESM2_ESM.pdf]

Hamre *et al. Systematic Reviews* (2023), https://doi.org/10.1186/s13643-023-02313-2

Additional file 2. Additional data on the comparison of MA of placebo-controlled trials of homoeopathic and conventional treatment, respectively in Shang (2005).

Additional data on Shang 2005

We here present data for the comparison of meta-analyses (MAs) of placebo-controlled trials of homoeopathic and conventional treatment, respectively, in Shang 2005.[1]

1. Methods

1.1 Research hypothesis

The authors (Shang 2005, Discussion, p.730) "*assumed that the effects observed in placebo-controlled trials of homoeopathy could be explained by a combination of methodological deficiencies and biased reporting*" and "*that the same biases could not explain the effects observed in comparable placebo-controlled trials of conventional medicine*".

1.2 Design, literature search and inclusion

Shang 2005 was designed as a prospective comparison of two MAs of placebo-controlled trials: one MA of any type of homoeopathic treatment for any disorder and one MA on conventional treatment, whereby each homoeopathy trial (HOM trial) was matched with a trial on conventional treatment (CON trial) "*that enrolled patients with similar disorders and assessed similar outcomes*" (Shang 2005, Methods, p.727).

Potentially eligible HOM trials were searched for in 19 electronic databases and by hand search of the Linde 1997 MA.[2] Matching CON trials were searched for in 1 database (Cochrane Controlled Trials Register, 2003, Issue 1). The authors "*used computer-generated random numbers to select one from several eligible trials of conventional medicine. Outcomes were selected and trials matched without knowledge of trial results*" (Shang 2005, Methods, p.727).

2. Results

2.1 All trials

2.1.1 Descriptive data, trial results

Among all trials, (n = 110 patients in each group) sample size was very similar (median 65.5 and 65.0 patients in HOM and CON trials, respectively, p = 0.875) (Table 1).

Regarding publication format, a significantly smaller proportion of HOM trials than CON trials were published as journal articles (85.5%, n = 94/110 vs. 100.0%, n = 110/110, p < 0.001), published in Medline-indexed journals (40.0% vs. 86.4%, p < 0.001) and published in the English language (52.7% vs. 85.5%, p < 0.001), respectively (Table 1).

Median publication year was 1992 and 1994 for HOM and CON trials, respectively. Outcome types and outcome assessments were very similar among HOM and CON trials, without any significant differences (Table 1).

In accordance with the matching, the distribution of indication groups was identical in the two trial sets (Table 2).

The proportion of trials with a significant effect of the intervention, compared to placebo was 36.4% (n = 40/110) for HOM trials and 50.0% (n = 55/110) for CON trials (p = 0.056) (Table 1).

Table 1 Characteristics of trials of homoeopathy and conventional medicine, respectively

	Homoeopathy (n = 110)		Conventional medicine (n = 110)		Comparison*
Sample size					Mann-Whitney U-test
Median	65.5		65.0		p = 0.875, median difference (HOM–CON) -1.0, 95% CI -14.0 to +10.0
Interquartile range	37.0-104.0		35-131.5		
Total range	10-1573		12-1367		
Mean (SD)	117.1 (211.2)		132.7 (225.9)		
Publication year					
Median year (range)	1992 (1966-2003)		1994 (1974-2002)		
Publication format	N	Percent	N	Percent	Fisher's exact test
Journal article	94	85.5%	110	100.0%	p < 0.001
Medline-indexed journal	45	40.9%	95	86.4%	p < 0.001
English language	58	52.7%	94	85.5%	p < 0.001
Type and assessment of outcome					
Overall assessment of response	54	49.1%	49	44.5%	p = 0.5890
Occurrence or duration of disorder	26	23.6%	26	23.6%	No difference
Assessment of symptoms	21	19.1%	26	23.6%	p = 0.5109
Measurement of function or state	6	5.5%	6	5.5%	No difference
Assessment of clinical signs	3	2.7%	3	2.7%	No difference
Trial results					
Intervention > Placebo significant (p < 0.05)	40	36.4%	55	50.0%	p = 0.056
Intervention > Placebo not significant	51	46.4%	38	34.5%	
Placebo > Intervention not significant	18	16.4%	16	14.5%	
Placebo > Intervention significant (p < 0.05)	1	0.9%	1	0.9%	
Methodological quality					
Described as double-blind	101	91.8%	96	87.3%	p = 0.3785
Adequate generation of allocation sequence	27	24.5%	30	27.3%	p = 0.7585
Adequate concealment of allocation	49	44.5%	21	19.1%	p < 0.0001
Intention-to-treat analysis	33	30.0%	40	36.4%	p = 0.3904
High-Quality trials	21	19.1%	9	8.2%	p = 0.0294

*Statistical tests analysed by authors of this systematic review.

Table 2 Indication groups of all trials of homoeopathy and conventional medicine, respectively

Indication groups	Homoeopathy		Conventional medicine		Total	
	N	Percent	N	Percent	N	Percent
Gastroenterology	12	10.9%	12	10.9%	24	10.9%
Gynaecology and obstetrics	14	12.7%	14	12.7%	28	12.7%
Musculoskeletal disorders	11	10.0%	11	10.0%	22	10.0%
Neurology	10	9.1%	10	9.1%	20	9.1%
Pollinosis and asthma	16	14.5%	16	14.5%	32	14.5%
Respiratory-tract infections	21	19.1%	21	19.1%	42	19.1%
Surgery and anaesthetics	12	10.9%	12	10.9%	24	10.9%
Miscellaneous	14	12.7%	14	12.7%	28	12.7%
TOTAL	110	100.0%	110	100.0%	220	100.0%

Indications and outcomes were listed in separate supplementary tables for HOM and CON trials, respectively, without apparent matching of individual trials. In Table 3 we have matched trials within each indication group for similar indications and similar outcomes.

Table 3 Indications and outcomes of all trials of homoeopathy and conventional medicine, respectively

HOMOEOPATHY				CONVENTIONAL MEDICINE			
**Author (ref)**	**Indications**	**Outcomes**	**ICD-10**	**Author (ref)**	**Indications**	**ICD-10**	**Outcomes**
Gastroenterology				Gastroenterology			
Solanki (85)	Intestinal amoebiasis and giardiasis	Global assessment, other	A06-A07	Rossignol (82)	Intestinal infection	A09	Global assessment, other
Mössinger (64)	Cholecystopathia	Global assessment, physician	A09	Sand (86)	Gallbladder stones	K80	Pain
Jacobs (46)	Diarrhoea	Duration of diarrhoea	A09	Alam (2)	Diarrhoea	A09	Duration of diarrhoea
Jacobs (48)	Diarrhoea	Duration of diarrhoea	A09	Cezard (18)	Diarrhoea	A09	Duration of diarrhoea
Jacobs (49)	Diarrhoea	Duration of diarrhoea	A09	Kaplan (53)	Diarrhoea	A09	Duration of diarrhoea
Ritter (79)	Gastropathy	Global assessment, other	K31	Battaglia (7)	Irritable bowel syndrome	K58	Global assessment, other
Mössinger (65)	Gastropathy	Global assessment, patient	K31	Skoubo-Kirstensen (93)	Gastritis	K29	Global assessment, patient
Mössinger (65)	Gastropathy	Global assessment, patient	K31	Nayak (69)	Irritable bowel syndrome	K58	Pain
Mössinger (65)	Irritable bowel syndrome	Global assessment, patient	K58	Gade (38)	Irritable bowel syndrome	K58	Global assessment, physician
Rahlfs (73)	Irritable bowel syndrome	Global assessment, patient	K58	Hovdenak (46)	Irritable bowel syndrome	K58	Global assessment, patient
Rahlfs (74)	Irritable bowel syndrome	Global assessment, patient	K58	Milo (64)	Irritable bowel syndrome	K58	Global assessment, other
Bignamini (12)	Anal fissures	Global assessment, patient	K60	Maria (57)	Anal fissure	K60	Global assessment, physician
Gynaecology & obstetrics				Gynecology & obstetrics			
Carey (18)	Vaginal discharge	Global assessment, other	N89	Stein (95)	Vaginal candidiasis	B37	Global assessment, physician
Ustianowski (92)	Cystitis	Global assessment, physician	N30	Warren (107)	Cystitis	N30	Global assessment, patient
Bergmann (10)	Amenorrhea	Global assessment, physician	N91	Mendes (63)	Amenorrhoea	N91	Global assessment, physician
Bergmann (10)	Oligomenorrhea	Global assessment, physician	N91	Fleming (35)	Oligomenorrhoea	N91	Global assessment, physician
Chapman (22)	Premenstrual syndrome	Global assessment, other	N94	Grio (41)	Premenstrual syndrome	N94	Global assessment, other
Lepaisant (58)	Premenstrual syndrome	Global assessment, other	N94	Di Carlo (28)	Premenstrual syndrome	N94	Depression
Yakir (104)	Premenstrual syndrome	Global assessment, patient	N94	Eriksson (33)	Premenstrual syndrome	N94	Global assessment, patient
Gauthier (39)	Menopausal complaints	Global assessment, patient	N95	Von Holst (106)	Postmenopausal complaints	N95	Flushes
Beer (9)	Childbirth	Time to uterine contraction	O80	McCaul (58)	Childbirth	O80	Time to delivery
Coudert (25)	Childbirth	Global assessment, other obs.	O80	Humphrey (49)	Childbirth	O80	Occurrence of preterm labour
Dorfman (32)	Childbirth	Occurrence of dystocia	O80	Demory (27)	Childbirth	O80	Dystocia
Hofmeyr (43)	Childbirth	Pain	O80	Hopkinson (44)	Childbirth	O80	Pain
Ives (45)	Childbirth	Pain	O80	Bettigole (12)	Childbirth	O80	Pain
Berrebi (11)	Breastfeeding	Pain	O92	Louviere (56)	Breastfeeding	O92	Pain

Musculoskeletal complaints				Musculoskeletal complaints			
Thiele (89)	Haemarthrosis	Global assessment, physician	M25	Ramesh (78)	Injuries	T14	Global assessment, patient
Mössinger (65)	Cramps	Global assessment, patient	R25	Memeo (61)	Soft tissue injury	T14	Global assessment, patient
Tveiten (91)	Physical activity	Soreness	M79	Grossman (42)	Physical activity	M79	Soreness
Vickers (95)	Physical activity	Soreness	M79	Semark (91)	Physical activity	M79	Soreness
Vickers (94)	Physical activity	Soreness	M79	Thompson (101)	Physical activity	M79	Soreness
Jawara (50)	Physical activity	Soreness	M79	Barlas (6)	Physical activity	M79	Pain
Casanova (20)	Myalgia	Global assessment, patient	M79	Weller (108)	Physical activity	M79	Global assessment, patient
Bohmer (15)	Sports injury	Global assessment, patient	T14	Lacey (54)	Soft tissue injury	T14	Global assessment, physician
Schmidt (83)	Subcutaneous mechani-cal injuries	Global assessment, patient	T14	Paddon-Jones (74)	Muscle damage	T14	Soreness
Zell5)	Sprain	Joint angulation	S93	McLatchie (59)	Ankle sprains	S93	Global assessment, physician
Dorfman (30)	Infusion	Pain	T80	Giner (39)	Arterial cannulation	T80	Pain
Neurology				Neurology			
Brigo (16)	Migraine	Global assessment, patient	G43	Diamond (29)	Migraine	G43	Global assessment, patient
Straumsheim (87)	Migraine	Frequency of attacks	G43	Storey (97)	Migraine	G43	Frequency of attacks
Whithmarsh (99)	Migraine	Migraine attacks	G43	Goldstein (40)	Migraine	G43	Migraine attacks
Savage (81)	Stroke	Death	I64	Sacco (84)	Stroke	I64	Death
Savage (82)	Stroke	Death	I64	Horn (45)	Stroke	I64	Occurrence of poor outcome
Master (59)	Aphasia	Global assessment, physician	R47	Huber (47)	Aphasia	R47	Global assessment, physician
Walach (96)	Headache	Pain	R51	Schmitt (89)	Headache	R51	Pain
Chapman (21)	Traumatic brain injury	Activities	S06	Narotam (68)	Brain injury	S07	Global assessment, physician
Dexpert (28)	Motion sickness	Global assessment, other	T75	Attias (4)	Seasickness	T75	Global assessment, patient
Ponti (72)	Seasickness	Global assessment, patient	T75	Shupak (92)	Seasickness	T75	Global assessment, patient
Pollinosis and asthma				Pollinosis and asthma			
Mokkapatti (63)	Conjunctivitis	Occurrence of conjunctivitis	H10	Davis (24)	Conjunctivitis	H10	Global assessment, physician
Mössinger (67)	Rhinitis	Global assessment, patient	J00	de Molina (26)	Rhinitis	J00	Global assessment, patient
Taylor (88)	Allergic rhinitis	Global assessment, patient	J30	Munk (67)	Allergic rhinitis	J30	Global assessment, patient
Wiesenauer (100)	Pollinosis	Ocular symptoms	J30	Althaus (3)	Pollinosis	J30	Ocular symptoms
Wiesenauer (101)	Pollinosis	Ocular symptoms	J30	Taudorf (100)	Pollinosis	J30	Ocular symptoms
Wiesenauer (103)	Pollinosis	Ocular symptoms	J30	Ciprandi (20)	Pollinosis	J30	Global assessment, physician
Aabel (1)	Pollinosis	Global assessment, patient	J30	Bodtger (16)	Pollinosis	J30	Global assessment, patient
Reilly (77)	Pollinosis	Global assessment, patient	J30	Brunet (17)	Pollinosis	J30	Global assessment, patient
Reilly (78)	Pollinosis	Global assessment, patient	J30	Wuthrich (109)	Pollinosis	J30	Global assessment, patient

Aabel (2)	Pollinosis	Global assessment, other	J30	Ciprandi (19)	Pollinosis	J30	Global assessment, other
Wiesenauer (102)	Pollinosis	Global assessment, other	J30	Moller (66)	Pollinosis	J30	Global assessment, other
Matusiewicz (61)	Asthma	FEV1	J45	Bergmann (10)	Asthma	J45	FEV1
Matusiewicz (61)	Asthma	FEV1	J45	Boos (14)	Asthma	J45	FEV1
Matusiewicz (60)	Asthma	FEV1/FVC	J45	Reid (79)	Asthma	J45	PEFR
Freitas (37)	Asthma	Occurrence of asthma	J45	Huerta Lopez (48)	Asthma	J45	Global assessment, physician
Reilly (76)	Asthma	Global assessment, patient	J45	Stelmach (96)	Asthma	J45	Global assessment, other
Respiratory tract infections				Respiratory tract infections			
Jacobs (47)	Otitis media	Global assessment, other	H66	Bertin (11)	Otitis media	H66	Pain
Mössinger (68)	Otitis media	Global assessment, physician	H66	Ruohola (83)	Otitis media	H66	Occurrence of acute otitis media
Weiser (97)	Sinusitis	Global assessment, other	J01	Frachet (36)	Sinusitis	J01	Global assessment, patient
Mössinger (66)	Pharyngitis	Duration of symptoms	J02	Nelson (71)	Pharyngitis	J02	Global assessment, other
de Lange (27)	Upper respiratory tract infection	Global assessment, other	J06	Hutton (50)	Common cold	J00	Global assessment, other
Diefenbach (29)	Upper respiratory tract infection	Global assessment, patient	J06	Schlenter (88)	Sinusitis	J01	Headache
Attena (5)	Influenza like disease	Occurrence of influenza like disease	J11	Crawford (22)	Influenza	J11	Occurrence of influenza like disease
Brydak (17)	Influenza like disease	Occurrence of influenza like disease	J11	De Flora (25)	Influenza like disease	J11	Occurrence of influenza like disease
Ferley (36)	Influenza like disease	Occurrence of influenza like disease	J11	Dolin (30)	Influenza	J11	Occurrence of influenza like disease
Heilmann (42)	Influenza like disease	Occurrence of influenza like disease	J11	Kaiser (52)	Influenza	J11	Occurrence of influenza like disease
Davies (26)	Influenza like disease	Occurrence of influenza like disease	J11	Belshe (8)	Influenza	J11	Occurrence of influenza
Nolleveaux (69)	Influenza like disease	Occurrence of influenza like disease	J11	Cohen (21)	Influenza	J11	Occurrence of influenza
Rottey (80)	Influenza like disease	Occurrence of symptoms	J11	Peters (76)	Influenza	J11	Occurrence of influenza
Lecocq (57)	Influenza like disease	Global assessment, patient	J11	Reuman (80)	Influenza	J11	Occurrence of influenza
Papp (71)	Influenza like disease	Global assessment, patient	J11	Van Voris (104)	Influenza	J11	Global assessment, other
Ferley (35)	Influenza like disease	Global assessment, physician	J11	Nicholson (72)	Influenza	J11	Global assessment, other
Casanova (19)	Influenza like disease	Temperature	J11	Treanor (102)	Influenza	J11	Temperature
Friese (38)	Adenoid vegetations	Global assessment, physician	J35	Sclafani (90)	Adenotons. hypertrophy	J35	Global assessment, physician
Hourst (44)	Respiratory tract infection	Occurrence of symptoms	J98	Van Eygen (103)	Respiratory tract infection	J98	Occurrence of symptoms

Torbieka (90)	Respiratory tract infection	Global assessment, physician	J98	Rodriguez (81)	Respiratory tract infection	J98	Global assessment, physician
Bordes (13)	Cough	Global assessment, patient	R05	Morrone (65)	Cough	R05	Global assessment, patient
Surgery & anaesthesiology				Surgery & anesthesiology			
Kaziro (53)	Tooth extraction	Pain	K08	Mehnert (60)	Tooth extraction	K08	Pain
Chevrel (23)	Postoperative ileus	Time to first wind	K91	Benson (9)	Postoperative ileus	K91	Time to first wind
Dorfman (31)	Postoperative ileus	Time to first wind	K91	Sadek (85)	Postoperative ileus	K91	Time to first wind
Estrangnin (34)	Postoperative ileus	Occurrence of first wind	K91	Boghaert (13)	Postoperative Ileus	K91	Occurrence of first wind
Aulagnier (6)	Postoperative ileus	Global assessment, patient	K91	Taguchi (99)	Postoperative ileus	K91	Occurrence of first wind
Valero (93)	Postoperative infection	Postoperative infection	T81	Crowley (23)	Postoperative infection	T81	Occurrence of postoperative infection
Ramelet (75)	Surgery	Occurrence of haematomas	Y83	Oertli (73)	Surgery	Y83	Occurrence of haematomas
Jeffrey (51)	Hand surgery	Pain	Z09	Schaffer (87)	Surgery	Y83	Pain
Stevinson (86)	Hand surgery	Pain	Z09	Hoernecke (43)	Hand surgery	Z09	Pain
Hart (41)	Hysterectomy	Pain	Z90	Ilkjaer (51)	Hysterectomy	Z90	Pain
Alibeu (3)	Postoperative agitation	Agitation	Z98	Mendel (62)	Postoperative agitation	Z98	Agitation
Kennedy (54)	Postoperative chest problems	Occurrence of complications	Z98	Negro (70)	Postoperative lung complications	J95	Occurrence of complications
Miscellaneous				Miscellanous			
Labreque (55)	Warts	Global assessment, physician	B07	Eron (34)	Condylomata acuminata	A63	Global assessment, physician
Kainz (52)	Warts	Area of warts	B07	Yazar (110)	Warts	B07	Global assessment, other
Schmidt (84)	Fasting	body weight	E66	Enzi (32)	Obesity	E66	Body weight
Werk (98)	Overweight	BMI	E66	Bahadori (5)	Obesity	E66	BMI
Cialdella (24)	Substitution of benzodiazepines	Global assessment, physician	F13	Petrovic (77)	Benzodiazepine withdrawal	F13	Global assessment, other
McCutcheon (62)	Anxiety	Anxiety	F41	Frattola (37)	Anxiety	F41	Anxiety
Awdry (7)	Post viral fatigue syndrome	Global assessment, patient	G93	Vercoulen (105)	Chronic fatigue syndrome	G93	Global assessment, patient
Ernst (33)	Varicosis	Pain	I83	Dominguez (31)	Venous insufficiency	I87	Pain
Oberbaum (70)	Stomatitis	Global assessment, other	K12	Adamietz (1)	Mucositis	K12	Global assessment, other
Balzarini (8)	Radiodermatitis	Global assessment, physician	L58	Lievens (55)	Radiotherapy	K12	Mucositis
Gibson (40)	Rheumatoid Arthritis	Global assessment, patient	M06	Boureau (15)	Rheumatoid Arthritis	M06	Global assessment, patient
Andrade (4)	Rheumatoid Arthritis	Global assessment, physician	M06	Smolen (94)	Rheumatoid Arthritis	M06	Global assessment, physician
Leaman (56)	Burns	Pain	T30	Patterson (75)	Burns	T30	Pain
Bourgois (14)	Infusion	Pain	T80	Taddio (98)	Injection	T80	Pain

Indications and outcomes: free text from Shang 2005. ICD-10 3-digit codes and matching of studies within each indication group provided by authors of this systematic review.

2.1.2 Methodological quality, heterogeneity

Four components of methodological quality were assessed, of which three (double blinding, adequate generation of allocation sequence, intention-to-treat analysis) did not differ among the two trial sets, while the proportion trials with adequate concealment of allocation was higher among HOM trials (44.5%, n = 49/110) than CON trials (19.1%, n = 21/110) (p < 0.0001). Likewise, the proportion of high-quality trials was higher for HOM trials (19.1%) than CON trials (8.2%) (p = 0.0294) (Table 1). Heterogeneity was significantly (F test, p = 0.011) less pronounced among HOM trials (I-squared 65%) than CON trials (I-squared 77%) (Table 4).

Table 4 Tests for statistical heterogeneity

Intervention	Chi-squared	I-squared
Homoeopathy	309, df 109, p < 0.0001	65%
Conventional medicine	481, df 109, p < 0.0001	77%

2.1.3 Funnel plot inspection and associated statistical tests

In both trial sets funnel plots were asymmetric, trials with higher standard error having larger effects. Likewise, Egger's test was significant for both trial sets, with less asymmetry for HOM trials than for CON trials (asymmetry coefficients 0.17 and 0.21, respectively, Table 5).

Table 5 Tests for funnel plot asymmetry

Trials	Funnel plot asymmetry?	Egger's test	
		Asymmetry coefficient (95%-CI)	p-value
Homoeopathy	Yes	0.17 (0.10-0.32)	p < 0.0001
Conventional medicine	Yes	0.21 (0.11-0.40)	p < 0.0001

2.1.4 Associations between methodological quality or subgroups and effect estimates

Univariate associations between quality components and diagnostic categories, respectively, and effect estimates were tested in both trial sets. Among HOM trials, significant associations were found for 3 of 7 single quality components (Medline-indexed, double-blinding, adequate generation of allocation sequence) as well as for high-quality trials. Among CON trials, the same eight tests showed no significant associations. In both trial sets, there were no significant associations between indication group and effect estimates, Furthermore, for HOM trials no significant associations were found between homoeopathy type and effect estimates (Table 6).

Table 6 Univariate associations between subgroups and effect estimates

	Homoeopathy			Conventional medicine		
Methodological quality	ROR (95%-CI)	p-value	Significant?	ROR (95%-CI)	p-value	Significant?
English language	0.73 (0.53-1.00)	p = 0.05	No	0.67 (0.40-1.14)	p = 0.114	No
Medline-indexed	0.69 (0.50-0.94)	p = 0.019	Yes	1.03 (0.61-1.75)	p = 0.906	No
Double-blinding	0.44 (0.22-0.87)	p = 0.017	Yes	0.63 (0.36-1.11)	p = 0.107	No
Adequate generation of allocation sequence	0.76 (0.48-0.95)	p = 0.024	Yes	0.98 (0.65-1.46)	p = 0.913	No
Adequate concealment	0.78 (0.57-1.07)	p = 0.117	No	0.76 (0.48-1.16)	p = 0.193	No
Intention-to-treat	1.25 (0.87-1.80)	p = 0.225	No	1.14 (0.78-1.66)	p = 0.506	No
High quality trials	0.62 (0.43-0.90)	p = 0.011	Yes	0.61 (0.34-1.09)	p = 0.095	No
Other subgroups						
Duration of follow-up	No data	p = 0.862	No	No data	p = 0.594	No
Indication group (n = 8)	No data	p = 0.660	No	No data	p = 0.360	No

ROR: Rate of odds ratios. 95%-CI: 95% confidence interval

In multivariate analyses, as summarised by the authors "the standard error of the log odds ratio (asymmetry coefficient) was the dominant variable in both groups. Coefficients of other variables, including study quality, were attenuated and became non-significant" (Shang 2005, pp. 729-730).

2.1.5 Primary outcomes of this systematic review

Meta-analytic effect estimates were not available for either of the two trial sets.

2.2 High-quality trials

2.2.1 Descriptive data

A total of 21 HOM and 9 CON trials were of high quality (individual trials listed in Table 7).

Table 7 High-quality trials of homoeopathy and conventional medicine, listed in order of descending sample size

Homoeopathy trials (n = 21)					Conventional medicine trials (n = 9)			
No.	Author (trial #)	Indication group	N		No.	Author (trial #)	Indication group	N
1	Rottey (80)	Influenza like disease	501		1	Horn (45)	Stroke	454
2	Vickers (94)	Physical activity	400		2	Nicholson (72)	Influenza	319
3	Papp (71)	Influenza like disease	334		3	Reuman (80)	Influenza	316
4	Schmidt (84)	Fasting	208		4	Crowley (23)	Postoperative infection	273
5	Labreque (55)	Warts	162		5	Kaplan (53)	Diarrhoea	256
6	Jacobs (46)	Diarrhoea	116		6	De Flora (25)	Influenza like disease	248
7	Weiser (97)	Sinusitis	104		7	Möller (66)	Pollinosis	146
8	Walach (96)	Headache	98		8	Humphrey (49)	Childbirth	89
9	Jacobs (49)	Diarrhoea	81		9	Gade (38)	Irritable bowel syndrome	54
10	Jacobs (47)	Otitis media	75					
11	Hart (41)	Hysterectomy	73					
12	Wiesenauer (102)	Pollinosis	72					
13	Zell (105)	Sprain	69					
14	Böhmer (15)	Sports injury	67					
15	Vickers (95)	Physical activity	57					
16	Jawara (50)	Physical activity	50					
17	Chapman (21)	Traumatic brain injury	50					
18	Tveiten (91)	Physical activity	46					
19	Stevinson (86)	Hand surgery	42					
20	Lepaisant (58)	Premenstrual syndrome	36					
21	Chapman (22)	Premenstrual syndrome	10					

"Large" high-quality trials (highlighted in yellow) identified in [3].

Among these, sample size was significantly lower in HOM trials than in CON trials (median 73.0 and 256.0 patients, respectively; median difference 154.0 patients, 95%-CI 17.0-223.0; p = 0.022, Table 8).

Table 8 Sample size of high-quality trials of homoeopathy and conventional medicine. respectively

Sample size	Homoeopathy (n = 21)	Conventional medicine (n = 9)	Mann-Whitney U-test
Median	73.0	256.0	p = 0.022, median difference (CON – HOM) 154.0, 95%-CI 17.0-223.0
Interquartile range	50.0-139.0	117.5-317.8	
Total range	10-501	54-454	
Mean (SD)	126.2 (129.8)	239.4 (125.3)	

Analysed by the authors of this systematic review

Following sample restriction to high-quality trials, the distribution of indications in the two trial subsets (Table 2) was altered, with 73% (n = 22/30) of trials unmatched (Table 9, also described in Rutten 2008[4], p.171).

Table 9 Matching by indication for high-quality trials of homoeopathy and conventional medicine, respectively

Indications, free text (ICD-10, 3-digit codes)	Homoeopathy		Conventional medicine		Total		Unmatched	
	N	Percent	N	Percent	N	Percent	N	Percent
Childbirth (O80)	0	0%	1	11%	1	3%	1	3%
Diarrhoea (A09)	2	10%	1	11%	3	10%	1	3%
Fasting (E66)	1	5%	0	0%	1	3%	1	3%
Hand surgery (Z09)	1	5%	0	0%	1	3%	1	3%
Headache (R51)	1	5%	0	0%	1	3%	1	3%
Hysterectomy (Z90)	1	5%	0	0%	1	3%	1	3%
Influenza OR Influenza-like disease (J11)	2	10%	3	33%	5	17%	1	3%
Irritable bowel syndrome (K58)	0	0%	1	11%	1	3%	1	3%
Otitis media (H66)	1	5%	0	0%	1	3%	1	3%
Physical activity (M79)	4	19%	0	0%	4	13%	4	13%
Pollinosis (J30)	1	5%	1	11%	2	7%	0	0%
Postoperative infection (T81)	0	0%	1	11%	1	3%	1	3%
Premenstrual syndrome (N94)	2	10%	0	0%	2	7%	2	7%
Sinusitis (J01)	1	5%	0	0%	1	3%	1	3%
Sports injury (T14)	1	5%	0	0%	1	3%	1	3%
Sprain (S93)	1	5%	0	0%	1	3%	1	3%
Stroke (I64)	0	0%	1	11%	1	3%	1	3%
Traumatic brain injury (S06)	1	5%	0	0%	1	3%	1	3%
Warts (B07)	1	5%	0	0%	1	3%	1	3%
TOTAL	21	100%	9	100%	30	100%	22	73%

Coding of indications and matching according to Table 3

2.2.2 Primary outcomes of this systematic review

Effect estimates were available for high-quality HOM trials (analysed in Lüdtke 2008[5]), showing a significant positive effect of homoeopathy, compared to placebo (Article, Table 12). For high-quality CON trials, no meta-analytic effect estimate was available.

2.3 Large high-quality trials

2.3.1 Descriptive data

Following sample restriction to "large" (Shang 2005, abstract) or "larger" (Shang 2005 p.730) high-quality trials, a total of 8 HOM and 6 CON trials remained. These trials were identified by the authors[3] and are highlighted in yellow in Table 7.

No criteria for "large/larger" trials were stated. It was not explained why the third largest high-quality CON trial (Reuman, CON trial no. 80, n = 316 patients) was not included among the "large/larger" trials. – In this case we made an exception to the principle of not reading individual trial publications when conducting a SR of SR (cf. Article, Section 'Data collection process'): the Reuman trial publication[6] presented two placebo-controlled randomised trials of the same drug, thereof one four-armed trial with altogether n = 78 patients and one three-armed trial with a very similar sample size (n = 318 or n = 317 patients for amantadine 100 mg or 200 mg vs placebo, respectively) to the reported sample size of n = 316 and results compatible with those reported in Shang 2005. We could thus confirm that this trial did have the third largest sample size among the high-quality CON trials (Table 7).

Median sample sizes were 185.0 and 264.5 for HOM and CON trials, respectively; the difference was not significant (p = 0.491, Table 10).

Table 10 Sample size of "large" high-quality trials of homoeopathy and conventional medicine. respectively

Sample size	Homoeopathy (n = 8)	Conventional medicine (n = 6)	Mann-Whitney U-test
Median	185.0	264.5	p = 0.491<br>median difference (HOM – CON)<br>-59.5 (95% CI -175.0 to + 144.5)
Interquartile range	107.0-385.5	222.5-352.75	
Total range	98-501	146-454	
Mean (SD)	240.38 (152.92)	282.67 (101.35)	

Analysed by the authors of this systematic review.

The 14 trials covered 10 different indications, with 57% (n = 8/14) of trials unmatched (Table 11).

Table 11 Shang 2005: Large high-quality trials of homoeopathy and conventional medicine, ordered by indication

Indications (free text)	Homoeopathy		Conventional medicine		Total		Unmatched	
	N	Percent	N	Percent	N	Percent	N	Percent of Total
Diarrhoea	1	13%	1	17%	2	14%	0	0%
Headaches	1	13%	0	0%	1	7%	1	7%
Muscle soreness	1	13%	0	0%	1	7%	1	7%
Plantar warts	1	13%	0	0%	1	7%	1	7%
Post operative infection	0	0%	1	17%	1	7%	1	7%
Prevention of influenza	1	13%	1	17%	2	14%	0	0%
Sinusitis	1	13%	0	0%	1	7%	1	7%
Stroke (venous)	0	0%	1	17%	1	7%	1	7%
Treatment of influenza	1	13%	2	33%	3	21%	1	7%
Weight loss	1	13%	0	0%	1	7%	1	7%
TOTAL	8	100%	6	100%	14	100%	8	57%

2.3.2 Combined sensitivity analyses

Effect estimates for large high-quality trials showed no significant difference between intervention and placebo for the 8 largest HOM trials and a positive significant effect compared to placebo for the 1st,2nd and 4th to 7th largest CON trials (Table 12).

Table 12 Effect estimates after sample restriction to "large" high-quality trials

Intervention	N trials		Odds ratio (95% confidence interval)	Significant?
	All	After sample restriction		
Homoeopathy	110	8	0.88 (0.65-1.19)	No
Conventional medicine	110	6	0.58 (0.39-0.85)	Yes

An odds ratio < 1 favours the intervention

2.4 The largest trials

Meta-regression analyses of "*predicted effect in trials as large as the largest trials*" (cf. Article, Section 'Secondary outcomes') showed no significant difference between intervention and placebo for HOM trials and a positive significant effect compared to placebo for CON trials (Table 13).

Table 13 Predicted effects in trials as large as the largest trials

Intervention	Odds ratio (95% confidence interval)	Significant?
Homoeopathy	0.96 (0.73-1.25)	No
Conventional medicine	0.67 (0.48-0.91)	Yes

Meta-regression analyses. An odds ratio < 1 favours the intervention

References

1. Shang A, Huwiler-Muntener K, Nartey L, et al. Are the clinical effects of homoeopathy placebo effects? Comparative study of placebo-controlled trials of homoeopathy and allopathy. *Lancet* 2005; **366**(9487): 726-32 https://doi.org/10.1016/S0140-6736(05)67177-2.
2. Linde K, Clausius N, Ramirez G, et al. Are the clinical effects of homoeopathy placebo effects? A meta-analysis of placebo-controlled trials. *Lancet* 1997; **350**: 834-43 https://doi.org/10.1016/s0140-6736(97)02293-9.
3. Shang A, Jüni P, Sterne JAC, Huwiler-Müntener K, Egger M. Are the clinical effects of homoeopathy placebo effects? - Authors' reply. *Lancet* 2005; **366**(9503): 2083-5 https://doi.org/10.1016/S0140-6736(05)67881-6.
4. Rutten AL, Stolper CF. The 2005 meta-analysis of homeopathy: the importance of post-publication data. *Homeopathy* 2008; **97**(4): 169-77 https://doi.org/10.1016/j.homp.2008.09.008.
5. Lüdtke R, Rutten AL. The conclusions on the effectiveness of homeopathy highly depend on the set of analyzed trials. *J Clin Epidemiol* 2008; **61**(12): 1197-204 https://doi.org/10.1016/j.jclinepi.2008.06.015.
6. Reuman PD, Bernstein DI, Keefer MC, Young EC, Sherwood JR, Schiff GM. Efficacy and safety of low dosage amantadine hydrochloride as prophylaxis for influenza A. *Antiviral Res* 1989; **11**(1): 27-40 https://doi.org/10.1016/0166-3542(89)90018-1.
