# Supplementary material for: Efficacy of homoeopathic treatment: Systematic review of meta-analyses of randomised placebo-controlled homoeopathy trials for any indication
Source: Syst Rev. 2023 Oct 7;12:191. doi: 10.1186/s13643-023-02313-2 (PMC10559431; doi:10.1186/s13643-023-02313-2)
Supplement: Supplementary file 3 — Additional file 3. Confidence in cumulative evidence for research questions 1 and 2, assessed according to the GRADE framework. [file 13643_2023_2313_MOESM3_ESM.pdf]

Hamre *et al. Systematic Reviews* (2023) https://doi.org/10.1186/s13643-023-02313-2

Additional file 3. Confidence in cumulative evidence for research questions 1 and 2, assessed according to the GRADE framework.

Confidence in cumulative evidence

We here present the assessments of confidence in cumulative evidence.

The assessments pertain to research questions 1 and 2 (cf. Article, Section 'Objectives'):

1. Does homoeopathic treatment have positive effects beyond placebo in MAs of randomised placebo-controlled trials for any condition?
2. Do the findings from these meta-analyses (MAs) support the notion of a common effect – or absence thereof – across different types of homoeopathic treatment (e.g., individualised, clinical, or complex homoeopathy) and across different types of indications (e.g., acute, chronic)?

Assessments are performed according to the conceptual framework of the Grading of Recommendations Assessment, Development and Evaluation (GRADE) group[1] (cf. Article, Section 'Methods' / 'Confidence in cumulative evidence').

1. Cumulative evidence #1: Positive effect of homoeopathy beyond placebo?

The cumulative evidence for Research question 1 is as follows: homoeopathy had significant positive effects beyond placebo in 5 of 5 MAs of all eligible trials (no data available from Shang 2005[2]).

According to the GRADE assessment system, the quality of this evidence (high/moderate/low/very low) is rated as high. The GRADE framework does, however, specify several possible occasions for rating down or rating up the quality of evidence[3], depending on what is called 'confidence in the cumulative evidence'. In Sections 1.1-7 of this document, this confidence is assessed according to the GRADE framework. The assessments pertain to all MAs as well as MAs grouped by scope: MAs of individualised homoeopathy (I-HOM), of nonindividualised homoeopathy (NI-HOM), and of all homoeopathy types (ALL-HOM = I-HOM + NI-HOM), as appropriate. In Section 1.8, the alternative hypothesis of no outcome difference between homoeopathy and placebo is assessed.

1.1 Risk of bias (methodological quality) of individual trials

Effect estimates from trials with high methodological quality may be closer to "true effects" than estimates from trials with lower quality. However, for trials of different indications and therapy modalities as in the MAs of this SR, such differences may also affect effect estimates independently of trial quality.

Significant associations between methodological quality and effect estimates were found in 12 of 24 analyses from 4 MA. Of these 12 analyses, one was of high-quality trials, and 11 were of single-quality components.

For 10 different single quality components, the proportion of trials fulfilling the respective criterion was assessed in at least two MAs, with a total of 43 analyses. Of these analyses, 44% (n = 19/43) showed a fulfilment rate of ≥50% of trials. Compared to trials with other interventions, the quality of the homoeopathy trials in the MAs of this SR was similar (Mathie 2014[4] & 2017[5]) or higher (Shang 2005).

In this SR, the predefined main outcome analysis with respect to methodological quality was the effect estimate after sample restriction to trials of higher quality, as defined by the authors of the respective MA, including at least 3 quality components, and restricted to one sample subset per MA. In 3 of 4 such analyses, the significant positive effects of homoeopathy beyond placebo were retained, thereof 2 MAs (Linde 1997[6], Shang 2005/Lüdtke 2008[7]) on ALL-HOM and 1 MA (Mathie 2014) on I-HOM. The significant positive effect was not retained in 1 MA (Mathie 2017) of NI-HOM.

Notably, all four MAs included three components pertaining to the key design elements randomisation and blinding ('generation of allocation sequence', 'allocation concealment' and 'blinding of patients and evaluators'), which were also associated with the effect estimate in 5 of 8 analyses in this SR. In a 5th MA (Cucherat 2000[8] on ALL-HOM), sample restriction to trials fulfilling these criteria – albeit not classified as high-quality trials in this SR – a significant positive effect of homoeopathy beyond placebo was also retained.

Conclusion: For NI-HOM (1 MA, Mathie 2017) there are reasons to rate down the quality of evidence because the significant positive effects were not retained after sample restriction to high-quality trials. For ALL-HOM and I-HOM, there is no occasion for rating down.

1.2 Inconsistency/heterogeneity

Inconsistency refers to variability in the magnitude of effects across trials. Inconsistency can be assessed graphically (variation of effect estimates, degree of overlap of confidence intervals) and mathematically as statistical heterogeneity.

Statistical heterogeneity of effect estimates was assessed in 4 MAs. Of these, 1 MA of I-HOM (Mathie 2014) showed no signs of heterogeneity, while 1 MA of NI-HOM (Mathie 2017) and 2 MAs of ALL-HOM (Linde 1997, Shang 2005) showed significant heterogeneity. In the MA conducted by Linde 1997, heterogeneity was reduced by restricting the sample to trials with higher methodological quality (6 of 7 analyses, τ-squared reduced from 0.43 to range 0.31-0.41), while a significant positive effect of homoeopathy remained in these trial subsets. In the MA conducted by Mathie 2017, heterogeneity was increased after trim-and-fill for FPA (I-squared 65% → 79%).

This statistical heterogeneity might reflect clinical heterogeneity with heterogeneous treatment effects for different indications, different NI-HOM interventions and possibly different outcome measures.[6]

Subgroup interaction tests did not show significant differences when contrasting low and high homoeopathic potencies (2 predefined analyses, Mathie 2014 & 2017), acute/chronic/prophylactic indications, eight diagnosis groups, or four homoeopathy types (1 analysis each, not predefined, Shang 2005).

Nonetheless, each type of NI-HOM comprises a range of interventions used for different indications, which may have heterogeneous effects that are not captured in analyses contrasting homoeopathy types.

Heterogeneity is relevant for Research question 2 (cf. Section 2). With 81% of 310 trials in the 6 MAs showing a superiority of homoeopathy over placebo and only 0.3% (one trial) showing a significant effect of placebo over homoeopathy, heterogeneity doers not seem relevant for Research question 1.

1.3 Risk of nonreporting bias and small study bias

1.3.1 Comparison of effect estimates in available and unavailable/unidentified trials

Direct assessments of nonreporting bias, including publication bias and location bias[9], (typically smaller treatment effects in unavailable or unidentified but eligible trials than in available trials) by comparison of effect estimates in available and unavailable/unidentified trials[10] were not found.

An indirect proxy assessment by comparison of effect estimates in 6 registered and 13 unregistered trials from Mathie 2017 showed no significant difference between the two estimates (Gartlehner 2022[11]). The significant positive effect of homoeopathy beyond placebo among all trials analysed by Mathie 2017 was retained for the 6 registered trials (with presumed low/zero risk of publication bias) and not retained for the 13 unregistered trials. The latter finding was weakened by the omission of 1 eligible unregistered trial from the analysis (Article, Section 'Additional data: Gartlehner 2022').[11]

1.3.2 Unavailable trials

Among three MAs (Linde 1997, Cucherat 2000 and Shang 2005), a total of 9 unavailable trial records were identified. Of these 9 records, 4 were assessed for eligibility in subsequent MAs but not included because of insufficient data (abstract only, Linde 1998[12], n = 1 record) or ineligible publication format (article in nonpeer-reviewed journal, Mathie 2014 & 2017, n = 3), while 5 records of unavailable trials remained unassessed. – In addition, 7 otherwise eligible HOM trials for Shang 2005 were not included, as no matching CON trials were found.

1.3.3 Unidentified trials

Mathie 2013 identified 25 and 41 trials that were potentially eligible for inclusion in the MAs conducted by Linde 1997 and Shang 2005, respectively, but they were not listed therein. Most of these reports (23 and 27, respectively) were not peer-reviewed publications and hence not eligible for inclusion in the MAs conducted by Mathie 2014 or Mathie 2017. The increased availability of full-text trial reports on the internet as well as improved and additional online databases during the period between these two MAs (published 1997-2005) and the analyses of Mathie (published 2013) may have contributed to the additional findings of Mathie 2013.

1.3.4 Funnel plot and associated analyses

Funnel plot inspection and associated tests were performed in 4 MAs. Of these, 3 MAs – assessing ALL-HOM (Linde 1997, Shang 2005) or NI-HOM (Mathie 2017) – showed FPA, with trials with higher standard error, which typically is correlated with lower sample size, having larger effects. One MA, assessing I-HOM (Mathie 2014), showed no signs of FPA.

FPA with larger effects in small trials may be associated with nonreporting bias or poor methodological quality with inflated effect estimates in smaller trials (methodological quality of individual trials is assessed in Section 1.1). However, FPA can have a number of other causes.

For all three MA with FPA, there was evidence for statistical heterogeneity of effect estimates, which may lead to FPA[13,14] (cf. Article, Section 'Heterogeneity'). Another possible cause of FPA in the three MAs is the outcome metrics: odds ratios (used in the MA conducted by Linde 1997 and Shang 2005[13,15,16]) and SMD (in the MAs conducted by Mathie 2017[17]) may both produce false-positive or exaggerated estimates of FPA.[13-17] Other technical problems include the use of Egger's test for binary outcomes (in Shang 2005 and Mathie 2014), for which this test is not recommended, and statistical testing for FPA in MA samples with fewer than 10 trials (one analysis in Shang 2005/Lüdtke 2008[13,14]).

On the other hand, FPA can reflect genuine larger treatment effects in smaller trials because of more restrictive eligibility criteria and thus more responsive patient population[9], trials on indications with known large effects and consequently lower sample sizes estimated a priori[7], better treatment implementation in smaller trials[9,14], and larger treatment effect in trials with high-risk patients who are difficult to recruit – leading to low sample sizes.[14] FPA may also occur by chance.[13,14,16]

None of the analyses reported in the Article, Section 'Secondary outcomes' can discern between true nonreporting bias/small study bias and the other causes of FPA described above.

1.3.5 Industry funding

Industry-funded trials might be more prone to publication bias than other trials. In the MAs conducted by Mathie 2014 & 2017, significant effects of homoeopathy were retained after sample restriction to trials rated as free from funding-related vested interests.

1.3.6 Trim-and-fill, adjustment for possible publication bias

The number of fictive additional trials with zero result to increase the p value to insignificance was 923 in the MA conducted by Linde 1997 (random-effects model, $p \geq 0.05$), 115 in the MA conducted by Cucherat 2000 (p value combination, $p > 0.05$) and 11 in the MAs conducted by Mathie (no

specification). Despite the limitations of these analyses[14], it should be noted that the n = 923 fictive trials estimated by Linde 1997 are 37 times higher than the unidentified additional 25 trials potentially eligible for that meta-analysis that were subsequently detected by Mathie 2013.

By using different methods and assumptions for adjustment for possible publication bias, a significant positive effect of homoeopathy beyond placebo was retained in the MAs conducted by Linde 1997 and Mathie 2017 but not the MA conducted by Shang 2005.

1.3.7 Discussion and conclusion

It is not possible to draw any conclusions from the findings of FPA in conjunction with heterogeneity and from the related analyses referred to above. The identification by Mathie 2013 of an additional 25 (28% of 89) and 41 (37% of 110) trials potentially eligible for the MAs conducted by Linde 1997 and Shang 2005, respectively, is more relevant. Both MAs concerned ALL-HOM. For Linde 1997, the trim-and-fill-analysis reported above indicates that any nonreporting bias would very unlikely change conclusions on a significant positive effect of homoeopathy.

For Shang 2005, the situation is different: the effect estimate for all 110 trials is unknown, and a significant effect was found in the Lüdtke 2008 analysis of high-quality trials but not in the extreme scenario regression model for large high-quality trials published in Shang 2005. The latter analysis is open to criticism for reliance on untestable assumptions about FPA and for the risk of introducing new bias by exclusion of relevant trials. Nonetheless, the body of 41 additional, potentially eligible trials identified by Mathie 2013 is not accounted for: without knowledge of eligibility, availability of outcome data extractable for meta-analysis, and the direction and magnitude of effect of these 41 trials, it remains uncertain if effect estimates for all trials or for high-quality trials would have been changed if they had been identified by Shang 2005. The same applies to the 7 otherwise eligible trials excluded by Shang 2005 because no matchable CON trials were found.

In summary, for the 28% (n = 25/89) additional trials of Linde 1997, any nonreporting bias would very unlikely change the conclusions. For the 41% (n = [41+7]/[110+7]) additional trials of Shang 2005, a relevant impact of nonreporting bias cannot be excluded. Because the absolute and relative body of unaccounted additional trials is larger for the MA conducted by Shang 2005 than for the earlier MA by Linde 1997, the MA by Shang has greater weight for this issue.

Accordingly, there is occasion for rating down the quality of evidence of a significant positive effect for ALL-HOM because of uncertainty about nonreporting bias in the MA conducted by Shang 2005.

1.4 Imprecision

Imprecision refers to a wide CI around effect estimates and the question of whether conclusions would differ if the upper or lower border of the CI represented the true effect.[18] For two analyses of high-quality trials, both showing significant positive effects of homoeopathy, imprecision is a potential concern: 95%-CI for effect estimate was

- very close to 'no difference' in Shang 2005/Lüdtke 2008: OR 0.76, 95%-CI 0.59-0.99 (<1 favouring homoeopathy), n = 21 trials
- fairly close to 'no difference' in the MAs conducted by Mathie 2014: OR 1.98, 95%-CI 1.16-3.38 (>1 favouring homoeopathy), n = 3 trials

The analysis of high-quality trials in Shang 2005/Lüdtke 2008 concerned ALL-HOM, for which more precise data are available from Linde 1997.

The analysis in the MAs conducted by Mathie 2014 is the only available analysis of high-quality trials of I-HOM. The result for high-quality trials needs to be seen in context, however. In the cumulative MAs with incremental removal of trials with the highest rated risk of bias from all trials (n = 22) to high-quality trials (n = 3) (Suppl. Table 31, lower part; Mathie 2014, Fig. 4)

- all effect estimates show a positive, significant effect of homoeopathy beyond placebo,
- upon incremental removal of trials, the effect size increases (No. 1-4: odds ratio 1.5 → 1.66), remains nearly stable (No 5-11: odds ratio 1.63-1.66) and increases again (No 11-13: odds ratio 1.66-1.98).
- while the lower end of the 95% confidence interval remains in the range 1.16-1.30.

The number of high-quality trials is low. Nonetheless, the results of this cumulative MA – with positive, significant, and even increasing effect sizes upon incremental removal of trials with lower quality – do not give occasion for rating down the quality of evidence.

1.5 Indirectness, subgroup analyses

Indirectness refers to differences between a set of trials to the topic of interest regarding patient populations, interventions or outcomes, as well as to indirect comparisons of interventions.[19]

1.5.1 Patient populations, interventions, outcomes

The topic of this SR was homoeopathy for all patients, homoeopathy types and clinical outcomes, and the included MAs had no relevant eligibility restrictions in this regard. The trials included in the MA covered 18 different countries, a broad range of indications, all four major homoeopathy types, and a variety of outcomes. Fifteen percent of trials included children only, with a small number of trials per MA (data available for 3 MAs on individualised or NI-HOM, respectively, with n = 4, n = 4, and n = 6 paediatric trials, respectively); thus, the applicability of results to homoeopathy for children is somewhat limited.

Effect estimates were analysed in subgroups pertaining to indication (acute or chronic), type of homoeopathy, homoeopathic potency and outcome metric in trials. Homoeopathy had positive effects beyond placebo in 21 of 23 analyses, including 14 of 15 predefined analyses.

1.5.2 Indirect comparisons of interventions

The MA conducted by Shang 2005 included an indirect comparison of two MAs of placebo-controlled trials of homoeopathy and conventional medicine, respectively, matched for similar indications and outcome types (not for publication format). This design is methodologically weak, and the matching by indication/outcome was of limited value: meta-analytic effect estimates were not published for the two full datasets, only for 30 (HOM 21, CON 9) high-quality trials and the 14 largest trials among these (while omitting the third largest CON trial); for these subsets, 73% and 57% of trials, respectively, were unmatched. Therefore, this comparison does not provide any reliable information on the comparative effects of HOM and CON. It does, however, yield comparative information on methodological quality, heterogeneity and FPA, which were published for the full sets of 110 HOM and 110 CON trials.

1.5.3 Conclusion

There was no reason to rate down the quality of evidence because of indirectness.

1.6 Occasions for rating up the quality of evidence

In the GRADE guidelines, three possible occasions for rating up the quality of evidence are described:

- Large effects: According to Cohen's criteria, large effect estimates are standardised mean difference ≥0.80[20], corresponding to odds ratios ≥5.[21] These criteria were not fulfilled for the primary outcomes of this SR.
- Dose–response gradient: not observed.
- Conclusion that plausible residual confounding would further support inferences regarding treatment effect: not applicable.

Conclusion: No occasion for rating up the quality of evidence.

1.7 Conclusion on significant positive effects of homoeopathy

The quality of evidence for significant positive effects of homoeopathy beyond placebo is moderate for ALL-HOM and NI-HOM and high for I-HOM (Table 1).

Table 1 Profile of evidence for significant positive effects of homoeopathy beyond placebo.

	All homoeopathy types	Individualised homoeopathy	Non-individualised homoeopathy
All trials, results	2 MAs (89 trials + 17 comparisons):<br>2x HOM>PLAC significant	2 MAs (18 + 22 trials):<br>2x HOM>PLAC significant	1 MA (54 trials):<br>1x HOM>PLAC significant
High-quality trials, results	1 MA (26 trials):<br>1x HOM>PLAC significant	1 MA (3 trials):<br>HOM>PLAC significant	1 MA (3 trials): HOM vs. PLAC no significant difference
Inconsistency	2 MAs (89 + 110 trials): 2x heterogeneity, not relevant for research question	1 MA (22 trials): no heterogeneity	1 MA (54 trials): heterogeneity, not relevant for research question
Indirectness	No data available on children. Otherwise, no serious indirectness	2 MAs (4 + 6 trials) on children without subgroup analyses. Otherwise, no serious indirectness	1 MA (4 trials) on children without subgroup analysis. Otherwise, no serious indirectness
Imprecision	Concern in 1 of 2 MAs of high-quality trials (21 trials). Otherwise, no serious imprecision	No serious imprecision	No serious imprecision
Non-reporting bias	2 MAs (89 + 110 trials): additional 25 and 41 trials potentially eligible but not listed trials, respectively, identified in a later MA.<br>1 MA (89 trials); Any publ. bias from the additional 25 trials would very unlikely change conclusions.<br>1 MA (110 trials): Residual uncertainty because of additional 41 potentially eligible trials + 7 trials excluded because unavailable for comparison to trials of conventional medicine.<br>2x: funnel plot asymmetry which could be explained by other factors	1 MA (22 trials): no funnel plot asymmetry.<br>4 trials free of funding-related vested interests: HOM>PLAC significant	1 MA funnel plot asymmetry, which could be explained by other factors.<br>11 trials free of funding-related vested interests: HOM>PLAC significant.<br>Adjustment for possible publication bias: HOM>PLAC significant
Quality of evidence	**Moderate quality** because of uncertainty about possible non-reporting bias in 1 MA	**High quality**	**Moderate quality** because of only 3 high-quality trials with HOM vs. PLAC no significant difference

Table adapted from reference no. [22], MA: Meta-analysis. HOM>PLAC significant: Significant positive effect of homoeopathy compared to placebo.

If the data sources were restricted to MAs with a low risk of bias (Linde 1997, Mathie 2014 & 2017), the quality of evidence would be changed to high for ALL-HOM and remain high for I-HOM and moderate for NI-HOM (Table 2).

Table 2 Profile of evidence for significant positive of effects of homoeopathy beyond placebo in meta-analyses with low risk of bias

	**All homoeopathy types (Linde 1997/1999)**	**Individualised homoeopathy (Mathie 2014)**	**Non-individualised homoeopathy (Mathie 2017)**
All trials, results	89 trials: HOM>PLAC significant	22 trials: HOM>PLAC significant	54 trials: 1x HOM>PLAC significant
High-quality trials, results	26 trials: HOM>PLAC significant	3 trials: HOM>PLAC significant	3 trials: HOM vs. PLAC no significant difference
Inconsistency	89 trials: heterogeneity, not relevant for research question	22 trials: no heterogeneity	54 trials: heterogeneity, not relevant for research question
Indirectness	No data available on children. Otherwise, no serious indirectness	6 trials on children without subgroup analyses. Otherwise, no serious indirectness	4 trials on children without subgroup analysis. Otherwise, no serious indirectness
Imprecision	No serious imprecision	No serious imprecision	No serious imprecision
Publication bias	Additional 25 potentially eligible but not listed trials were identified in a later MA. However, 923 fictive additional trials with zero result would be required in order to increase p-value to ≥ 0.05 (random-effects model). Funnel plot asymmetry which could be explained by other factors.	No data on unavailable trial records.<br>22 trials: no funnel plot asymmetry.<br>4 trials free of funding-related vested interests: HOM>PLAC significant	54 trials: funnel plot asymmetry which could be explained by other factors.<br>11 trials free of funding-related vested interests: HOM>PLAC significant
Quality of evidence	**High quality**	**High quality**	**Moderate quality** because of only 3 high-quality trials with HOM vs. PLAC no significant difference.

Table adapted from reference no. [22], MA: Meta-analysis. HOM>PLAC significant: Significant positive effect of homoeopathy compared to placebo.

1.8 Alternative hypothesis: no outcome difference between homoeopathy and placebo

In case of no outcome difference between homoeopathy and placebo, one would expect the following:

- A similar proportion of trials show the superiority of either intervention. This was not the case: a superiority was found for homoeopathy in 80.6% of trials and for placebo in 19.4% of trials, and a significant positive effect was found in 36.5% vs. 0.3% of trials, respectively (Article, Table 7).
- Most meta-analytic effect estimates show no significant differences between homoeopathy and placebo. This was not the case: significant positive effects of homoeopathy compared to placebo were found in 5 of 5 analyses of all eligible trials and 3 of 4 analyses (or 4 of 5 when including the MA conducted by Cucherat 2000, cf. Article, Section ‘Assessments of bias and heterogeneity’) of high-quality trials.
- The hypothesis in the MA conducted by Shang 2005 that all effects of homoeopathy could be explained by a combination of [A] risk of bias of individual trials (Section 1.1, above) and [B] publication bias/small study bias (Section 1.3) cannot be substantiated:

[A] Effect estimates after sample restriction to high-quality trials showed a significant positive effect of homoeopathy compared to placebo in 3 of 4 MA, including the MA conducted by Shang 2005/Lüdtke 2008 analysis. No small study bias (i.e., inflated effect estimates because of quality deficiencies of small trials) was demonstrated.

[B] Shang's hypothesis on [B] relied on FPA, which was assessed in 4 MA. Of these, all 3 MAs with FPA also had statistical heterogeneity, which, together with other factors, could explain FPA. The MA conducted by Mathie 2014 had no FPA and no heterogeneity. Thus, FPA was clearly associated with statistical heterogeneity, suggesting that heterogeneity was a driver of FPA, while the postulated publication bias/small study bias could not be substantiated in any analysis.

In conclusion, the available data yield no support for the hypothesis of no outcome difference between homoeopathy and placebo.

2. Cumulative evidence #2: Common effect across different treatments and indications?

In this section, the cumulative evidence for a common effect across different homoeopathic treatments and different indications (Research question 2 in the Article, Section 'Background and rationale') is assessed.

2.1 Different types of homoeopathic treatment

2.1.1 Comparisons between homoeopathy types

One subgroup interaction test showed no significant effect difference between the 4 homoeopathy types (p = 0.636, Shang 2005).

Effect estimates or p value combinations for different homoeopathy types showed a significant positive effect of homoeopathy compared to placebo in 11 of 12 analyses (not for the homoeopathic combination product in the MAs conducted by Mathie 2017) Table 3).

Table 3 Effect estimates for different homoeopathy types

	Linde 1997	Cucherat 2000	Mathie 2014	Mathie 2017
Metric	Odds Ratio (95%-CI)	p-value combination	Odds Ratio (95%-CI)	Stand. Mean Diff. (95%-CI)
Favours homoeopathy	>1	<0.05	>1	>0
Homoeopathy type				
Individualised	2.91 (1.57-5.37) 13 trials	p = 0.021 3 trials	1.53 (1.22-1.91) 22 trials	
Non-individualised		p = 0.00011 14 trials		0.33 (0.21-0.44) 54 trials
-Clinical	2.00 (1.60-2.51) 49 trials			0.28 (0.09-0.47) 23 trials
-Complex	2.94 (2.12-4.08) 20 trials			0.53 (0.27-0.79) 15 trials
-Combination product				0.20 (-0.05 to +0.45) 9 trials
-Isopathy	5.04 (2.24-11.32) 7 trials			0.21 (0.07-0.36) 7 trials

95%-CI: 95% confidence interval

2.1.2 Statistical homogeneity within homoeopathy types

For I-HOM, no statistical heterogeneity was found (1 MA, I-squared 0% [95%-CI 0%-40%], Mathie 2014).

For NI-HOM, significant heterogeneity was found (1 MA, I-squared 65%, τ-squared 0.11, p<0.0001, Mathie 2017). Likewise, significant heterogeneity was found for ALL-HOM combined (2 MA, Linde

1997, Shang 2005). The latter finding might originate from NI-HOM, as this homoeopathy type was used in 85% and 84% of trials, respectively, in the two MAs (Article, Table 7).

2.1.3 Comparisons between different homoeopathic potencies

Subgroup interaction tests showed no significant effect difference between high (≥ 12C) and low (< 12C) homoeopathic potencies (2 MA, Mathie 2014 & 2017).

Effect estimates for subgroups pertaining to homoeopathic potency showed a significant effect of homoeopathy compared to placebo in 5 of 6 analyses (not for medium and low potencies [$\leq 10^{-13}$ mol/L] in I-HOM) (Table 4).

Table 4 Effect estimates for different homoeopathic potencies and indication types

Author year	Homoeopathy type	Subgroup	N trials	Metric	Effect estimate (95% conf. int.)	Favours homoeo.	Significant?
		Potency [concentration]					
Linde 1997	All	High [$\leq 10^{-27}$ mol/L]	31	OR	2.66 (1.83-3.87)	>1	Yes
Mathie 2014	Individualised	High (≥12C)	8	OR	2.00 (1.38-2.88)	>1	Yes
Mathie 2017	Non-individualised	High (≥12C)	21	SMD	0.21 (0.05-0.36)	>0	Yes
Linde 1997	All	High + medium*	51	OR	2.77 (2.09-3.67)	>1	Yes
Mathie 2014	Individualised	Medium + low (<12C)	14	OR	1.30 (0.98-1.73)	>1	No
Mathie 2017	Non-individualised	Medium + low (<12C)	33	SMD	0.42 (0.25-0.60)	>0	Yes
		Indication type					
Mathie 2017	Non-individualised	Acute	38	SMD	0.34 (0.19-0.49)	>0	Yes
Mathie 2017	Non-individualised	Chronic	16	SMD	0.26 (0.11-0.41)	>0	Yes

OR: Odds ratio. SMD: Standardised Mean Difference. *$\leq 10^{-13}$mol/L

2.1.4 Conclusions

The notion of a common positive effect is

- supported for effects across different homoeopathy types, including different subtypes of NI-HOM,
- supported for effects of I-HOM,
- <u>not</u> supported for effects of NI-HOM.

As the MA of NI-HOM comprised different indications treated with different homoeopathic products, the latter finding suggests that the effects of NI-HOM may differ across different indications and/or different homoeopathic products used. Such effect differences may include significant positive effects of NI-HOM as well as no significant difference between NI-HOM and placebo in different subgroups.

2.2 Different types of indications

2.2.1 Analysis results

Subgroup interaction tests showed no significant effect difference between acute or chronic or prophylactic indications (1 MA, p = 0.487, Shang 2005). Effect estimates showed a significant effect of homoeopathy compared to placebo for acute as well as chronic indications (1 MA, Mathie 2017, Table 4).

2.2.2 Conclusion

The limited data available support the notion of a common positive effect of homoeopathy for acute as well as chronic indications. The issue of effect differences among different diagnoses or diagnosis groups is outside the scope of this SR.

References

1. Guyatt G, Oxman AD, Akl EA, et al. GRADE guidelines: 1. Introduction-GRADE evidence profiles and summary of findings tables. *J Clin Epidemiol* 2011; **64**(4): 383-94 https://doi.org/10.1016/j.jclinepi.2010.04.026.

2. Shang A, Huwiler-Muntener K, Nartey L, et al. Are the clinical effects of homoeopathy placebo effects? Comparative study of placebo-controlled trials of homoeopathy and allopathy. *Lancet* 2005; **366**(9487): 726-32 https://doi.org/10.1016/S0140-6736(05)67177-2.

3. Balshem H, Helfand M, Schünemann HJ, et al. GRADE guidelines: 3. Rating the quality of evidence. *J Clin Epidemiol* 2011; **64**(4): 401-6 https://doi.org/10.1016/j.jclinepi.2010.07.015.

4. Mathie RT, Lloyd SM, Legg LA, et al. Randomised placebo-controlled trials of individualised homeopathic treatment: systematic review and meta-analysis. *Syst Rev* 2014; **3**: 142 https://doi.org/10.1186/2046-4053-3-142.

5. Mathie RT, Ramparsad N, Legg LA, et al. Randomised, double-blind, placebo-controlled trials of non-individualised homeopathic treatment: systematic review and meta-analysis. *Syst Rev* 2017; **6**(1): 63 https://doi.org/10.1186/s13643-017-0445-3.

6. Linde K, Clausius N, Ramirez G, et al. Are the clinical effects of homoeopathy placebo effects? A meta-analysis of placebo-controlled trials. *Lancet* 1997; **350**: 834-43 https://doi.org/10.1016/s0140-6736(97)02293-9.

7. Lüdtke R, Rutten AL. The conclusions on the effectiveness of homeopathy highly depend on the set of analyzed trials. *J Clin Epidemiol* 2008; **61**(12): 1197-204 https://doi.org/10.1016/j.jclinepi.2008.06.015.

8. Cucherat M, Haugh MC, Gooch M, Boissel JP. Evidence of clinical efficacy of homeopathy. A meta-analysis of clinical trials. HMRAG. Homeopathic Medicines Research Advisory Group. *Eur J Clin Pharmacol* 2000; **56**(1): 27-33 https://doi.org/10.1007/s002280050716.

9. Guyatt GH, Oxman AD, Montori V, et al. GRADE guidelines: 5. Rating the quality of evidence--publication bias. *J Clin Epidemiol* 2011; **64**(12): 1277-82 https://doi.org/10.1016/j.jclinepi.2011.01.011.

10. Boutron I, Page MJ, Higgins JPT, et al. Concerning bias and conflicts of interests among the included studies. In: Higgins JPT, Thomas J, Chandler J, et al., eds. Cochrane Handbook for systematic reviews of interventions. 2. ed. Chichester: John Wiley & Sons; 2019: 177-204.https://doi.org/10.1002/9781119536604.ch7.

11. Gartlehner G, Emprechtinger R, Hackl M, et al. Assessing the magnitude of reporting bias in trials of homeopathy: a cross-sectional study and meta-analysis. *BMJ Evid Based Med* 2022; **27**: 345-51 http://dx.doi.org/10.1136/bmjebm-2021-111846.

12. Linde K, Scholz M, Ramirez G, Clausius N, Melchart D, Jonas WB. Impact of study quality on outcome in placebo-controlled trials of homeopathy. *J Clin Epidemiol* 1999; **52**(7): 631-6 https://doi.org/10.1016/s0895-4356(99)00048-7.

13. Sterne JA, Sutton AJ, Ioannidis JP, et al. Recommendations for examining and interpreting funnel plot asymmetry in meta-analyses of randomised controlled trials. *BMJ* 2011; **343**: d4002 https://doi.org/10.1136/bmj.d4002.

14. Page MJ, Higgins JPT, Sterne JAC. Assessing risk of bias due to missing results in a synthesis. In: Higgins JPT, Thomas J, Chandler J, et al., eds. Cochrane Handbook for systematic reviews of interventions. 2. ed. Chichester: John Wiley & Sons; 2019: 728.https://doi.org/10.1002/9781119536604.ch13.

15. Lau J, Ioannidis JP, Terrin N, Schmid CH, Olkin I. The case of the misleading funnel plot. *BMJ* 2006; **333**(7568): 597-600 https://doi.org/10.1136/bmj.333.7568.597.

16. Schwarzer G, Rücker G. Statistical methods for detecting and adjusting for publication bias. *Z Evid Fortbild Qual Gesundhwes* 2010; **104**(4): 306-13 https://doi.org/10.1016/j.zefq.2010.03.016.

17. Zwetsloot PP, van der Naald M, Sena ES, et al. Standardized mean differences cause funnel plot distortion in publication bias assessments. *Elife* 2017; **6**: https://doi.org/10.7554/elife.24260.

18. Guyatt GH, Oxman AD, Kunz R, et al. GRADE guidelines: 6. Rating the quality of evidence--imprecision. *J Clin Epidemiol* 2011; **64**(12): 1283-93 https://doi.org/10.1016/j.jclinepi.2011.01.012.

19. Guyatt GH, Oxman AD, Kunz R, et al. GRADE guidelines: 8. Rating the quality of evidence--indirectness. *J Clin Epidemiol* 2011; **64**(12): 1303-10 https://doi.org/10.1016/j.jclinepi.2011.04.014.

20. Cohen J. Statistical power analysis for the behavioral sciences. 2. ed. Hillsdale, NJ: Lawrence Erlbaum; 1988.

21. Chen H, Cohen P, Chen S. How big is a big odds ratio? Interpreting the magnitudes of odds ratios in epidemiological studies. *Commun Stat Simul Comput* 2010; **39**(4): 860-4 https://doi.org/10.1080/03610911003650383.

22. Guyatt G, Oxman AD, Sultan S, et al. GRADE guidelines: 11. Making an overall rating of confidence in effect estimates for a single outcome and for all outcomes. *J Clin Epidemiol* 2013; **66**(2): 151-7 https://doi.org/10.1016/j.jclinepi.2012.01.006.
