# Supplementary material for: Efficacy of homoeopathic treatment: Systematic review of meta-analyses of randomised placebo-controlled homoeopathy trials for any indication
Source: Syst Rev. 2023 Oct 7;12:191. doi: 10.1186/s13643-023-02313-2 (PMC10559431; doi:10.1186/s13643-023-02313-2)
Supplement: Supplementary file 4 — Additional file 4. Supplementary Tables. [file 13643_2023_2313_MOESM4_ESM.pdf]

Additional file 4: Supplementary tables

Heading levels and texts of this file correspond to the levels and texts of the article.

Methods

Data items

Other variables

Suppl. Table 1 Other variables collected from the meta-analyses

Topic	Items
Publication subject	Protocol for MA / primary publication of MA results / additional analyses of MA
Research questions of the MA	
Eligibility criteria for trials in the MA	Design: Randomisation? Blinding? Parallel group? Crossover trials?
	Publication types: Language restriction? Publication format restriction?
	Patients: Restrictions regarding age / gender / indications?
	Definition of homoeopathy
	Interventions: Prevention? Treatment of existing symptoms or diseases? Types of homoeopathic treatment included
Protocol mentioned in publication?	Stated as predefined? Pre-published?
Literature searches	End search (YYYY-MM-DD), Manuscript submitted (YYYY-MM-DD)
	Electronic databases searched: Number and names of databases
	Previous MA or SR consulted? Other searches, e.g., grey literature, hand searches? Contact with experts? Contact with pharmaceutical companies?
Quality of trial data handling	Screening of titles and abstracts, assessment of full text for inclusion, data extraction, assessment of trial quality/risk of bias (for each item: performed by one person / by two persons, one checking the other / by two persons, independently / other)
Excluded trials	List of excluded trials?
	Reason provided for exclusion of each trial?
Included trials	N eligible trials (or comparisons): trials without sufficient data for MA / included for MA
	Year range for trial publication
	Sample size, country and language of trial publications
	N trials with continuous or rank-ordered outcomes / with binary outcomes

	Funding source of trials
	Data presented on individual trials (name of each item)
Patient characteristics	Age, gender, indications
Homoeopathic treatment	Homoeopathy type: Individualised OR Classical / Non-individualised (Clinical homoeopathy, Complex homoeopathy, Isopathy, other).
	Potency/concentration: classification and criteria (e.g., “low potency: < Cx”, “high dilution: ≥ Cy”), N trials with high potencies
Assessment of risk of bias (methodological quality) of trials in the MA	Name of risk of bias instruments used in the MA
	Name of each quality components used. Total number of components used in each MA.
	Assessment of outcome reporting bias
	High-quality trials: criteria, described as predefined?
Heterogeneity, meta-bias	Data on unavailable and unidentified trials
	Statistical heterogeneity test findings
	Funnel plot inspection findings, asymmetry coefficient, other tests for possible publication bias / small study bias (name and result of test)
Results of individual trials (significance at p > 0.05 level): n trials with a…	significant positive effect of homoeopathy compared to placebo
	non-significant superiority of homoeopathy
	non-significant superiority of placebo
	significant positive effect of placebo, compared to homoeopathy

Results

Identification, screening and inclusion of meta-analyses

Suppl. Table 2 List of excluded reports with reasons for exclusion

Reasons for exclusion	Publications
Not a systematic review with meta-analysis of randomised controlled trials	Évaluation des médicaments homéopathiques soumis à la procédure d'enregistrement prévue à l'article l.5121-13 du CSP. Avis 26 juin 2019: Haute Autorité de santé, Direction de l'Evaluation Médicale, Economique et de Santé Publique. Commission de la Transparence, 2019.
	Antonelli M, Donelli D. Reinterpreting homoeopathy in the light of placebo effects to manage patients who seek homoeopathic care: A systematic review. Health Soc Care Community 2019; 27(4): 824-47.
	Chanda P, Furnham A. Does homoeopathy work? Part I: A review of studies on patient and practitioner reports. Focus Altern Complement Ther 2008; 13(2): 82-9.
	Chanda P, Furnham A. Does homoeopathy work? Part II: A review of recent scientific papers. Focus Altern Complement Ther 2008; 13(3): 157-67.
	Raza SA, Saleem Z. Homeopathic remedies to heal afflicted persons: A systematic review. SAJ Pharmacy and Pharmacology 2018; 5(2):1-11.
	Sigurdson MK, Sainani KL, Ioannidis JPA. Homeopathy can offer empirical insights on treatment effects in a null field. J Clin Epidemiol. 2023;155:64-72.
	Van Wassenhoven M. Meta-analyses des travaux récents en clinique homéopathique. Revue Belge d'Homoeopathie 2000; 52(1): 51-72.
	von Ammon K, Torchetti L, Frei-Erb M. Ergebnisse von Original-RCTs mit individueller Homöopathie und Hochpotenzen im Vergleich zu Placebo und Standard-Therapien. Der aktuelle Stand der Forschung zur Homöopathie: Versorgungsforschung, randomisierte kontrollierte klinische Studien, Meta-Analysen, Grundlagenforschung. Köthen (Anhalt): Wissenschaftliche Gesellschaft für Homöopathie; 2016: 13-23.
	Waisse S. Clinical research in homeopathy: systematic reviews and randomized clinical trials. Rev homeopatia (Sao Paulo) 2017; 80(3/4): 121-33.
Therapeutic benefit not assessed	Mathie RT, Wassenhoven MV, Jacobs J, et al. Model validity of randomised placebo-controlled trials of individualised homeopathic treatment. Homeopathy 2015; 104(3): 164-9.
	Mathie RT, Van Wassenhoven M, Jacobs J, et al. Model validity and risk of bias in randomised placebo-controlled trials of individualised homeopathic treatment. Complement Ther Med 2016; 25: 120-5.
Publication criteria not fulfilled	Eizayaga J. The Lancet e o proclamado fim da homeopatia: revisão crítica da publicaçãode Shang et al (2005) e dos artigos relacionados subsequentes. Rev homeopatia (Sao Paulo) 2013; 76(1/2).
	Ernst E, Pittler MH. Statistical comments on a re-analysis of a previous meta-analysis of homeopathic RCTs. J Clin Epidemiol 2002; 55: 2.
	Kahn MF, Seed P, Paterson C, et al. Meta-analysis of homoeopathy trials. Lancet 1998; 351(9099): 365-8.

Description of meta-analyses

Methods of the meta-analyses

Suppl. Table 3 Main research objective or question for meta-analyses

Author, year	Main research objective or hypothesis (quotation source)
Linde 1997	"…to assess whether the clinical effect reported in RCTs of homoeopathic remedies equivalent to that reported for placebo" (Abstract)
Linde 1998	"…to summarize the actual state of clinical efficacy research on individualised homoeopathy" (Abstract). "…to give an overview of the current state of the approaches, results and problems of the available RCTs on individualised homoeopathy that should be useful for future research" (Discussion)
Cucherat 2000	"To establish …whether there is any evidence from RCTs of the efficacy of homoeopathic treatment in patients with any disease" (Abstract)
Shang 2005	"We assumed that the effects observed in placebo-controlled trials of homoeopathy could be explained by a combination of methodological deficiencies and biased reporting." (Discussion)
Mathie 2014	To test "the hypothesis that the outcome of an individualised homoeopathic treatment approach using homoeopathic medicines is distinguishable from that of placebos". (Abstract)
Mathie 2017	To test "the null hypothesis that the main outcome of treatment using a non-individualised (standardised) homoeopathic medicine is indistinguishable from that of placebo" (Abstract)

Suppl. Table 4 Eligibility criteria for meta-analyses: Design, publication types

Design	Response categories	Linde 1997	Linde 1998	Cucherat 2000	Shang 2005	Mathie 2014	Mathie 2017
Randomisation [RCT]? Quasi-randomised [QRCT]?	0: Not a criterion, 1: RCT, 2: RCT or DB, 3: RCT or QRCT, 4: RCT or DB or QRCT	2	4	1	3	1	1
Double blinding [DB]?	0: Not a criterion, 1: DB, 2: DB or placebo control, 3: DB or RCT	3	3	0	2	3	3
Parallel group included?	0: No, 1: Yes, 2: Not stated	1	2	2	1	1	1
Crossover trials included?	0: No, 1: Yes, 2: Not stated	0	2	2	0	0	0
N-of-1-trials = single case experiments included?	0: No, 1: Yes, 2: Not stated	0	2	2	0	2	2
**Publication type**							
Language restriction?	0: No, 1: Yes, 2: Not stated	0	0	0	0	2	2
Publication format restrictions?	0: No, 1: Yes	0	0	0	0	1*	1*

*Published peer-reviewed journal articles of at least 500 words.

Suppl. Table 5 Eligibility criteria for meta-analyses: Patients and indications

Item	Response categories	Linde 1997	Linde 1998	Cucherat 2000	Shang 2005	Mathie 2014	Mathie 2017
Age restriction?	0: No, 1: Yes, 2: Not stated	2	2	2	2	0	0
Gender restriction?	0: No, 1: Yes, 2: Not stated	2	2	2	2	0	0
Provings with healthy subjects excluded?	0: No, 1: Yes, 2: Not stated	1	1	2	1	1	1

Suppl. Table 6 Eligibility criteria for meta-analyses: Interventions, comparators

Items (0: No, 1: Yes)	Linde 1997	Linde 1998	Cucherat 2000	Shang 2005	Mathie 2014	Mathie 2017
Interventions						
Prevention?	1	1	0	1	0	0
Treatment of existing symptoms/disease?	1	1	1	1	1	1
Homoeopathy definition						
Conceptual definition?	1	1	0	1	1	1
Operational definition?	0	0	1	0	1	1
Types of homoeopathic treatment						
Individualised = classical homoeopathy	1	1	1	1	1	0
Clinical homoeopathy	1	0	1	1	0	1
Complex homoeopathy	1	0	1	1	0	1
Isopathy	1	0	1	1	0	1
Comparators						
Other than placebo?	0	1	0	0	0	0

Suppl. Table 7 Eligibility criteria for meta-analyses: other

**Author, year**	**Other eligibility criteria?**	
	0: No, 1: Yes	Free text
Linde 1997	1	Inclusion: Sufficient data for calculation of outcome rates in both groups
Linde 1998	0	
Cucherat 2000	1	Inclusion: One clearly defined primary outcome
Shang 2005	1	Inclusion: Sufficient data for calculation of outcome rates.<br>Exclusion: Homoeopathy trials with indications for which no matched trial of conventional medicine could be found.
Mathie 2014 & Mathie 2017	1	Exclusion: Radionically prepared „homoeopathic" medicines, anthroposophic medicine*, homotoxicology*; intervention is homoeopathy combined with other (complementary or conventional) medicine or therapy, trials described as single-blind

*Statements in different publications not unequivocal. Mathie 2013: "Studies fulfilled the inclusion criteria if they used an explicitly labelled 'anthroposophic' or 'homotoxicologic' medicine prepared homoeopathically and whose dilution (or that of each of its components) was ≥1X". Mathie 2014-Protocol: "Homoeopathic medicines are also used in other therapeutic approaches such as anthroposophic medicine and homotoxicology, which are not the subject of our review work described below". Mathie 2017-Protocol: Anthroposophic medicine mentioned but not as an exclusion criterion.

Suppl. Table 8 Literature searches

**Item**	**Response categories**	**Linde 1997**	**Linde 1998**	**Cucherat 2000**	**Shang 2005**	**Mathie 2014**	**Mathie 2017**
Timeline							
End search	YYYY-MM-DD	1995-10	1998-05	1998-06	2003-01-31	2013-12-31	2014-12-31
Manuscript of meta-analysis submitted	YYYY-MM-DD	ND	ND	1999-08-19*	ND	2014-06-26	2016-09-19
Previous meta-analysis or systematic review consulted?	0: No, 1: Yes	1	1	1	1	1	1
Which?	Free text	Kleijnen 1991 (1)	Linde 1997	Kleijnen 1991 (1) Linde 1997	Linde 1997	Linde 1997; Shang 2005	Linde 1997; Shang 2005; Mathie 2014
Electronic databases searched							
Number		8	4	8	19	11	11
Names	Free text	Medline, EMBASE, Woodward Foundation, CISCOM, AMED HomInform, IDAG, CCRH	Medline, EMBASE, HOMINFORM, Cochrane Trials Registry	Medline, Embase, Biosis, PsychInfo, Cinahl, British Library Stock Alert Service, SIGLE, Amed	Medline, Pre-Medline, EMBASE, DARE, CCTR, CDSR, CINAHL, AMED, MANTIS, Toxline, PASCAL, BIOL, Sci Cit Index, CISCOM, Br. Homoeopathic Library, HomInform Homoeopathic library, NCCAM, SIGLE	AMED, CAM-Quest, CINAHL, Cochrane Central Register of Controlled Trials, Embase, Hom-Inform, LILACS, PubMed, Sci Cit Index, Scopus, CORE-Hom.	
Further searches							
Contact with experts	0: No, 1: Yes	1	1	1	1	1	1
Contact with pharmaceutical companies	0: No, 1: Yes	1	0	1	0	0	0
Hand search of reference lists, bibliography of text books and similar	0: No, 1: Yes	1	0	1	1	1	1
Other searches?	0: No, 1: Yes	1**	0	0	0	0	0

*The Boissel 1996 report, on which the Cucherat 2000 paper is based, was published in December 1998. **Individual collections, homoeopathic meetings

Suppl. Table 9 Outcome extraction criteria

**Author, year**	**Criteria predefined?**	**Criteria reported?**	**Criteria**
	0: No, 1: Yes	0: No, 1: Yes	Free text
Linde 1997, Linde 1998	1	1	Hierarchical: 1. Outcome used for sample size calculation; 2. patients' global assessment of improvement; 3. physicians' global assessment of improvement; 4. most important, judged by reviewers; 5. random selection of eligible outcomes
Cucherat 2000	1	1	Defined primary outcome in publication
Shang 2005	1	1	Hierarchical: 1. Outcome used for sample size calculation; 2. patients' global assessment of improvement; 3. physicians' global assessment of improvement; 4. most important, judged by reviewers. If several outcomes were judged equally relevant. one outcome was identified by random selection.
Mathie 2014, Mathie 2017	1	1	Hierarchical, based on WHO ICF Classification System for Levels of Functioning Linked to Health Condition): 1. Mortality; 2. Morbidity, 3. Health impairment; 4. Limitation of activity, 5. Restriction of participation; 6. Surrogate outcome

Suppl. Table 10 Definition/description of different homoeopathy types, verbatim citations from the meta-analyses

**Homoeopathy types**	**Description**
Individualised = classical homoeopathy	
Linde 1997	Single homoeopathic remedy selected, based on the total symptom picture of a patient
Linde 1998	Operational criteria: prescribers completely free in their choice of remedy OR prescribers free in their choice from a list of remedies frequently used for the condition OR inclusion restricted to patients with symptoms matching homoeopathic drug picture
Shang 2005	Comprehensive homoeopathic history-taking, followed by the prescription of a single individualised remedy, possibly with subsequent change of remedy in response to changing symptoms
Mathie 2014	Typically involves a long interview between the practitioner and the patient
Mathie 2017*	Typically, a single homoeopathic medicine is selected on the basis of the „total symptom picture" of a patient, including his/her mental, general and constitutional type
Clinical homoeopathy	
Linde 1997	One or several single remedies administered for standard clinical situations or conventional diagnoses.
Linde 1998	Use of a single remedy in patients with a conventional diagnosis; use of fixed combinations of several remedies in patients with a conventional diagnosis
Shang 2005	No comprehensive, homoeopathic history was taken, all patients received a single, identical remedy
Mathie 2017*	One or more homoeopathic medicines are administered for standard clinical situations or conventional diagnoses
Complex homoeopathy	
Linde 1997	Multiple remedies mixed into a standard formula to cover a person's symptoms and diagnoses
Shang 2005	Prescription of a mixture of several different remedies
Mathie 2017	More than one medicine is used in a fixed preparation as a… 'complex' or OTC-complex homoeopathic medicine (available as an over the-counter [OTC] proprietary formulation)
Homoeopathic combination product	
Mathie 2017	More than one medicine is used in a fixed preparation as a 'combination' (devised by researchers)
Isopathy	
Linde 1997	Serial agitated dilutions made from the causative agent in an infectious or toxicological condition (as with vaccination)
Linde 1998	Use of causative agents in potency
Shang 2005	The agent that was judged to be the cause of the disorder was used (for example, pollen in pollinosis)
Mathie 2017*	Use of homoeopathic dilutions from the causative agent of the disease itself, or from a product of the disease process, to treat the condition.

*Identical description also in protocols for Mathie 2014 & 2017. There are two within-group differences among the descriptions: 1. Shang 2005 limits 'clinical homoeopathy' to the use of single products, while Linde 1997 & 1998 and Mathie 2017 include the use of more than one product. 2. Mathie 2017 separates 'Homoeopathic combination product' from the category 'complex homoeopathy', which is not the case in Linde 1997 and Shang 2005.

Suppl. Table 11 3 or 4 of quality components used to define high-quality trials in Shang 2005?

Number of components	Statement (in Lüdtke 2008: criteria)
3 components:<br>I. Generation of allocation sequence,<br>II. Randomisation concealment,<br>III. Double-blinding	“Trials described as double-blind, with adequate methods for the generation of allocation sequence and adequate concealment of allocation, were classified as of higher methodological quality” (Shang 2005, p. 728, left column, lines 24-28)
	“Trials described as double-blind, with adequate generation of allocation sequence and adequate concealment of allocation.” (Shang 2005, Table 2, Table note to ‘Higher quality’)
	Sample restriction criteria for Shang 2005: High-quality + Intention-to-treat principle (Lüdtke 2008, Table 2)
4 components:<br>I-III +<br>IV. Intention-to-treat analysis	“Assessment of study quality focused on three key domains of internal validity: randomisation (generation of allocation sequence and concealment of allocation), masking (of patients, therapists, and outcome assessors), and data analysis (by intention to treat or other” (Shang 2005, p.728, left column, lines 5-10)
	“…variables: …trial quality (masking, generation of allocation sequence, concealment of allocation, intention-to-treat analysis)” (Shang 2005, p. 728, right column, lines 14-18)

Suppl. Table 12 Quality instruments and components used for analysis

Items	Linde 1997	Linde 1998	Cucherat 2000	Shang 2005	Mathie 2014	Mathie 2017
Name of quality instruments	Jadad score, IV Scale	Jadad score, IV Scale	NA	NA	Cochrane risk-of-bias appraisal tool [RoB 1]	Cochrane risk-of-bias appraisal tool [RoB 1]
Quality components						
1. Generation of allocation sequence	1	1	0	1	1	1
2. Randomisation concealment	1	1	0	1	1	1
3. Double-blinding [OR: A+B]	1	1	1	1	0	0
4. ..A. Blinding of patients	1	1	0	0	1*	1*
5. ..B. Blinding of evaluators	1	1	0	0	1	1
6. Baseline comparability	1	1	0	0	0	0
7. Selection bias after randomisation	1	1	0	0	0	0
8. Incomplete outcome data	0	0	0	0	1	1
9. Dropout/withdrawals described	1	1	1†	0	0	0
10. Intention-to-treat analysis	0	0	0	1	0	0
11. Statistical analysis adequate	1	1	0	0	0	0
12. Selective outcome reporting	1	0	1††	0	1	1
13. Medline-indexed publication	1	1	0	1	0	0
14. English language publication	0	0	0	1	0	0
15. Publication free of funding-related vested interests	0	0	0	0	1	1
Other sources of bias?	0	1	0	1	1	1
Other sources, description	NA	Other obvious relevant flaws	NA	NA	Other sources of bias†††	Other sources of bias†††
N quality components	10**	10**	3	6	8	8

IV: Internal Validity. * Mathie 2014 & 2017: Blinding of participants and study personnel. **Component no. 3 not counted, because it is redundant with no. (4 +5). †Cucherat 2000: Dropout rate < 10% and < 5%, respectively. ††An eligibility criterion for Cucherat 2000 was 'trials with a clearly defined primary outcome', which corresponds to this quality component. †††apart from components 1-2, 4-5, 8, 12

Suppl. Table 13 High-quality trials, cumulative meta-analyses

Items	Response categories	Linde 1997	Linde 1998	Cucherat 2000	Shang 2005	Mathie 2014	Mathie 2017
High-quality trials							
N quality components		7	Not applicable	Not applicable	3 or 4	7	7
Described as predefined?	0: No, 1: Yes, 2: In part	1	Not applicable	Not applicable	0	2	1
Association between quality descriptors and outcome with hypothesis testing	0: No, 1: Yes	1	0	0	1	1	1
Cumulative meta-analysis							
with rank-ordered categories	0: No, 1: Yes	0	0	1	0	0	0
with interval-scaled categories	0: No, 1: Yes	1	0	0	0	1	1

Suppl. Table 14 Heterogeneity, meta-bias

Items (0: not used, 1: used)	Linde 1997	Linde 1998	Cucherat 2000	Shang 2005	Mathie 2014	Mathie 2017
Statistical heterogeneity test?	1	0	0	1	1	1
Funnel plot inspection?	1	0	0	1	1	1
Egger's test (asymmetry coefficient)?	0	0	0	1	1	1
Trim-and-fill?	1	0	1	1	0	1
Other test?	1*	0	0	0	0	0
Adjustment for funnel plot asymmetry?	1	0	1	0	0	1
Small studies: sample restriction?	0	0	0	1	0	0
Selective outcome reporting bias assessed?	1	0	1	0	1	1
How was selective outcome reporting bias assessed?	Sensitivity analysis; Predefined main outcome	Not applicable	Inclusion criterion: one defined main outcome	Not applicable	Comparison results vs. protocol or methods section	Comparison results vs. protocol or methods section

* Random-effects and non-parametric selection models

Trial characteristics

Suppl. Table 15 Overview of publications, trials or trial comparisons, and meta-analyses (MA)

Publ. No.	Year	First author	Condition	Intervention	MA*	N MA
1	2000	Aabel	Pollinosis	Betula C30	4	1
2	2001	Aabel	Pollinosis	Betula C30	4	1
3	1984	Albertini	Dental neuralgia	Arnica C7, Hypericum C15	1	1
4	1990	Alibeu	Postoperative agitation	Aconitum C4	1,3,4	3
5	1991	Andrade	Rheumatoid arthritis	Individual treatment	1,2,4	3
6	1995	Attena	Influenza like disease	Oscillococcinum 200C	4	1
7	1985	Aulagnier	Postoperative ileus	Opium C9, Raphanus C9, Arnica C9	1,4	2
8	1996	Awdry	Postviral fatigue syndrome / Heroin detoxification	Individual treatment	2,4	2
9	2003	Baker	Anxiety	Single remedy	6	1
10	2000	Balzarini	Radiodermatitis	Belladonna C7, X-ray C15	4,6	2
11	1999	Beer	Childbirth	Caulophyllum D4	4,6	2
12	1993	Bekkering	Menopause	Famosan	1	1
13	2004	Bell	Fibromyalgia	Individual treatment	5	1
14	2006	Belon	Arsenic toxicity	Single remedy	6	1
15	2000	Bergmann (a)	Oligomenorrhea	Phyto-Hypophyson L	4,6	2
15	2000	Bergmann (b)	Amenorrhea	Phyto-Hypophyson L	4,6	2
16	2001	Berrebi	Breastfeeding	Apis mellifica C9, Bryonia C9	4	1
17	1987	Bignamini	Hypertension	Single remedy	6	1
18	1991	Bignamini	Anal fissures	Acidum nitricum C9	1,4	2
19	1992	Böhmer	Sports injury	Traumeel	1,4	2
20	2003	Bonne	Anxiety	Individual treatment	5	1
21	1986	Bordes	Cough	Drosetux	1,4	2
22	1984	Bourgois	Haematomas	Arnica C5	1,4	2
23	2011	Brien	Rheumatoid arthritis	Individual treatment	5	1
24	1991	Brigo	Migraine	Individual treatment in C30	1,2,4	3
25	1999	Brydak	Influenza like disease	Gripp-heel	4	1
26	1976	Campbell	Bruises	Arnica C30	1	1
27	1986	Carey	Vaginal discharge	Candida C30	1,4	2
28	1981	Casanova	Myalgia	Urathone	1,4	2
29	1992	Casanova	Influenza like disease	Oscillococcinum	1,4	2
30	2003	Cavalcanti	Uraemic pruritus	Individual treatment	5	1
31	1999	Chapman	Brain injury	Individual treatment	4,5	2
32	1994	Chapman	Premenstrual syndrome	Individual treatment	1,2,4	3
33	1984	Chevrel	Postoperative ileus	Opium C15	1,4	2
34	2001	Cialdella	Substitution of benzodiazepines	Homeogene 46	4,6	2
35	2000	Clark	Plantar fasciitis	Single remedy	6	1
36	2012	Colau	Menopause syndrome	OTC complex	6	1
37	2010	Cornu	Post-operative bleeding	Combination of remedies	6	1
38	1981	Coudert	Dystocia	Caulophyllum C5	1,3,4	3
39	1971	Davies	Prevention, URI	'Common cold' tablets	1,4	2
40	1994	de Lange	Recurrent upper respiratory tract infection	Individual treatment	1,2,4, 5	4
41	1987	Dexpert	Seasickness	Cocculine	1,4	2
42	1997	Diefenbach	Bronchitis	Bronchiselect	3,4,6	3
43	1988	Dorfman	Haematomas	Arnica C5	1,4	2
44	1992	Dorfman	Postoperative ileus	Raphanus C5, Arnica C9, China C5	1,4	2
45	1987	Dorfman	Childbirth	Arnica C5, Pulsatilla C5, Geranium C5	1,4	2
46	1990	Ernst	Varicosis	Poikiven	1,4,6	3
47	1983	Estrangin	Postoperative ileus	Arnica C7, China C7, Pyrogenium C5	1,4	2
48	1989	Ferley	Influenza-like syndrome	Oscillococcinum	1,3,4, 6	4

49	1987	Ferley	Influenza like disease	L52 (complex)	1,4	2
50	2006	Fisher	Eczema	Individual treatment	5	1
51	1989	Fisher	Fibrositis	Rhus toxicodendron C6	1,2	2
52	2005-1	Frass	Sepsis	Individual treatment	5	1
53	2005-2	Frass	Trachea secretions	Single remedy	6	1
54	1995	Freitas	Childhood asthma	Blatta orientalis C6	1,4,6	3
55	1997	Friese	Adenoid vegetations	Nux vomica, Okoubaka, Tuberculinum, Barium jodatum	4,6	2
56	1993	Gaus	Rheumatoid arthritis	Rheumaselect	3	1
57	1983	Gauthier	Menopausal complaints	Lachesis C30	1,4	2
58	1998	Gerhard (a)	Female infertility: amenorrhoea	OTC complex	6	1
58	1998	Gerhard (b)	Female infertility: lutea insufficiency	OTC complex	6	1
58	1998	Gerhard (c)	Female infertility: idiopathic	OTC complex	6	1
59	1980	Gibson	Rheumatoid Arthritis	Individual treatment	1,2,4	3
60	1989	Grecho	Postoperative ileus	Opium C15	3	1
60	1989	Grecho	Postoperative ileus	Opium C15, Raphanus C5	1,3,6	3
61	1987	Hariveau	Cramps	Cuprum C15	1	1
62	1997	Hart	Hysterectomy	Arnica C30	4	1
63	1992	Heilmann	Influenza like disease	Engystol	1,4	2
64	1990	Hofmeyr	Postpartum pain	Arnica D6	1,4,6	3
65	1981	Hourst	Respiratory tract infection	Thuja C9, two other remedies	1,4	2
66	1996	Ives	Childbirth	Arnica 30	4	1
67	1993	Jacobs	Childhood diarrhoea	Individual treatment in C30	1,2,4	3
68	1994	Jacobs	Childhood diarrhoea	Individual treatment in C30	1,2,3, 4	4
69	2000	Jacobs	Childhood diarrhoea	Individual treatment	4,5	2
70	2001	Jacobs	Otitis media, acute	Individual treatment	4,5	2
71	2005	Jacobs (a)	ADHD	Individual treatment	5	1
71	2005	Jacobs (b)	Menopause post breast cancer	Individual treatment	5	1
72	2007	Jacobs	Dengue fever symptoms	Combination of remedies	6	1
73	1997	Jawara	Physical activity	Arnica C30, Rhus toxicodendron C30	4	1
74	2002	Jeffrey	Hand surgery	Arnica D6	4	1
75	1996	Kainz	Warts	Individual treatment	2,4,5	3
76	1984	Kaziro	Tooth extraction, postoperative pain/swelling	Arnica C200	1,4,6	3
77	1971	Kennedy	Postoperative chest problems	Arnica C200	1,4	2
78	2005	Khuda-Bukhsh	Arsenic toxicity	Single remedy	6	1
79	2011	Khuda-Bukhsh	Arsenic toxicity	Single remedy	6	1
80	2005	Kim	Seasonal allergic rhinitis	Isopathic remedy	6	1
81	1991	Köhler	Rheumatoid arthritis	Rheumaselect	1	1
82	2010	Kotlus	Post-operative bruising	Combination of remedies	6	1
83	1986	Kubista	Mastodynia	Mastodynon	1	1
84	1992	Labrecque	Warts	Thuja C30, Antimonium crudum C5, Acidum nitricum C7	1,4	2
85	1989	Leaman	Minor burns	Cantharis C200	1,4,6	3
86	1985	Lecocq	Influenza like disease	L52 (complex)	1,4	2
87	1994	Lepaisant	Premenstrual syndrome	Folliculinum C9	1,4	2
88	2002	Lewith	Allergic asthma	Isopathic remedy	6	1
89	1992	Lièvre	2nd and 3rd degree burns	Calendula	3	1
90	1999	Lipman	Snoring	OTC complex	6	1
91	1995	Lökken	Tooth extraction, pain after oral surgery	Individual treatment in D30	1,2	2
92	2014	Malapane	Tonsillitis	OTC complex	6	1
93	1987	Master	Broca's aphasia in stroke patients	Individual treatment	1,2,4	3
94	1999	Matusiewicz	Asthma	Asthma H	4	1

95	1997	Matusiewicz (a)	Asthma	Traumeel S + Engystol N	4	1
95	1997	Matusiewicz (b)	Asthma	Traumeel S + Engystol N	4	1
96	1996	McCutcheon	Anxiety	Anti-anxiety	4	1
97	1994	McDavid	Agne vulgaris	Individual treatment	2	1
98	1981	Michaud	Tooth extraction	Apis C7, Arnica C15	1	1
99	1992	Mokkapatti	Conjunctivitis	Euphrasia C30	1,4	2
100	1976-1	Mössinger (a)	Gastropathy	Nux vomica D4	1,4	2
100	1976-1	Mössinger (b)	Gastropathy	Nux vomica D30	1,4	2
100	1976-1	Mössinger (c)	Cramps	Cuprum D4	1,4	2
100	1976-1	Mössinger (d)	Cramps	Cuprum D30	1	1
100	1976-1	Mössinger (e)	Cramps	Cuprum D200	1	1
100	1976-1	Mossinger (f)	Irritable bowel syndrome	Asa foetida, Nux vomica	4	1
101	1976-2	Mössinger	Pharyngitis	Phytolacca D2	1,4	2
102	1980	Mössinger	Boils and pyoderma	Hepar sulfuris calcerum D4	1,3	2
103	1982	Mössinger	Running nose	Euphorbium D3	1,4	2
104	1984	Mössinger	Cholecystopathia	Absinthium D2	1,4	2
105	1985	Mössinger	Otitis media	Pulsatilla D2	1,4	2
106	2013	Naidoo	Allergic skin reaction	Combination of remedies	6	1
107	1994	Nollevaux	Prevention URI	Mucococcinum 200K	1,4	2
108	2001	Oberbaum	Stomatitis	Traumeel	4,6	2
109	2005	Oberbaum	Postpartum bleeding	Combination of remedies	6	1
110	2011	Padilha	Lead poisoning	Single remedy	6	1
111	1998	Papp	Influenza like disease	Oscillococcinum	3,4,6	3
112	2008	Paris	Post-operative analgesic intake	Combination of remedies	6	1
113	1943	Paterson (a)	Skin lesions	Mustard gas C30	1	1
113	1943	Paterson (b)	Skin lesions	Individual treatment	1	1
113	1943	Paterson (c)	Skin lesions	Rhus tox C30	1	1
113	1943	Paterson (d)	Skin lesions	Mustard gas C30	1	1
114	1986	Ponti	Seasickness	Nux vomica C2, Cocculus C2, Tabacum C2	1,4	2
115	1979	Rahlfs	Irritable bowel syndrome	Asa foetida D3	1,4	2
116	1978	Rahlfs	Irritable bowel syndrome	Single remedy	6	1
117	1976	Rahlfs	Irritable bowel syndrome	Asa foetida, Nux vomica	1,4,6	3
118	2000	Ramelet	Surgery	Arnica C5	4	1
119	1999	Rastogi (a)	HIV	Individual treatment	5	1
119	1999	Rastogi (b)	HIV	Individual treatment	5	1
120	1985	Reilly	Pollinosis	Pollen C30	1,4	2
121	1986	Reilly	Pollinosis	Pollen C30	1,3,4, 6	4
122	1994	Reilly	Allergic asthma	Individual treatment	1,3,4, 6	4
123	1966	Ritter	Gastropathy	Nux vomica D4	1,4	2
124	2007	Robertson	Postoperative pain	Single remedy	6	1
125	1995	Rottey	Influenza like disease	Mucococcinum 200K	4	1
126	2008	Sajedi	Cerebral palsy	Individual treatment	5	1
127	1977	Savage	Stroke	Arnica C30	1,4	2
128	1978	Savage	Stroke	Arnica M	1,4	2
129	1996	Schmidt	Subcutaneous mechanical injuries	Arnica 6C	4	1
130	2002	Schmidt	Fasting	Thyroidinum C30	4	1
131	1990	Schwab (a+b)	Dermatoses + remedy picture of sulfur	Sulphur	2	1
131	1990	Schwab (a)	Dermatoses + remedy picture of sulfur	Sulphur	1	1
131	1990	Schwab (b)	Dermatoses + remedy picture of sulfur	Sulphur	1	1
132	1983	Shipley	Osteoarthritis	Rhus toxicodendron D6	1	1
133	2009	Siebenwirth	Eczema	Individual treatment	5	1

134	2010	Singer	Post-operative pain	OTC complex	6	1
135	1995	Solanki	Intestinal amoebiasis and giardiasis	Individual treatment	2,4	2
136	2003	Stevinson	Hand surgery	Arnica C6	4	1
137	2000	Straumsheim	Migraine	Individual treatment	4,5	2
138	1997	Straumsheim	Migraine	Individual treatment	2	1
139	2000	Taylor	Perennial allergic rhinitis	Isopathic remedy	4,6	2
140	1991	Thiel	Haemarthrosis	Traumeel	1,3,4	3
141	2005	Thompson	Menopause post breast cancer	Individual treatment	5	1
142	1998	Torbieka	Respiratory tract infection	Engystol	4	1
143	1991	Tveiten	Muscle soreness	Single remedy	6	1
144	1998	Tveiten	Physical activity	Arnica D30	4,6	2
145	1974	Ustianowski	Cystitis	Staphisagria C30	1,4	2
146	1981	Valero (a)	Previous postoperative infection	Pyrogenium C7	1,4	2
147	1981	Valero (b)	Postoperative ileus	Raphanus C7	1	1
148	1998	Vickers	Muscle soreness	Arnica 30X	4,6	2
149	1997	Vickers	Physical activity	Arnica C30, Rhus toxicodendron C30, Sarc a C30	4	1
150	1997	Walach	Headache	Individual treatment	2,4	2
151	2004	Weatherley-Jones	Chronic fatigue syndrome	Individual treatment	5	1
152	1994	Weiser	Chronic sinusitis	Euphorbium comp	1,4	2
153	1994+ 1995	Weiser	Chronic sinusitis	Euphorbium comp.	3	1
154	1994	Werk	Overweight	Helianthus tuberosus D1	1,4	2
155	1993	Whitmarsh	Headache	Individual treatment	3	1
156	1997	Whitmarsh	Migraine	Individual treatment	2,4,5	3
157	1983	Wiesenauer	Pollinosis	Galphimia glauca D4	1,4	2
158	1985	Wiesenauer	Pollinosis	Galphimia glauca D6	1,4,6	3
159	1990	Wiesenauer	Pollinosis	Galphimia glauca C2	1,4,6	3
160	1995	Wiesenauer	Pollinosis	Galphimia glauca D4	1,4,6	3
161	1991	Wiesenauer	Rheumatoid arthritis	Rheumaselect	1,6	2
162	2003	Wolf	Post-operative pain	Single remedy	6	1
163	2001	Yakir	Premenstrual syndrome	Individual treatment	4,5	2
164	2007	Zabolotnyi	Sinusitis	OTC complex	6	1
165	1988	Zell	Acute ankle sprains	Traumeel	1,3,4	3
Total						310

*MA: Included in meta-analyses: 1 = Linde 1997, 2 = Linde 1998, 3 = Cucherat 2000, 4 = Shang 2005, 5 = Mathie 2014, 6 = Mathie 2017. N MA: Number of meta-analyses in which the trial was included.

Suppl. Table 16 Summary descriptive data on included trials in text or table (excluding design, trial quality and results)

No.	Summary descriptive data on trials (0: No, 1: Yes)	Linde 1997	Linde 1998	Cucherat 2000	Shang 2005	Mathie 2014	Mathie 2017
1.	Indications/diagnoses (n different)	0	0	0	0	1	1
2.	Indication type: acute/(subacute)/chronic	0	1	0	0	0	1
3.	Indication groups (n different)	1	0	0	1	1	1
4.	Countries	1	1	0	0	0	0
5.	Languages (n different)	1	0	1	0	0	0
6.	Publication years, range	1	0	0	1	0	0
7.	Sample size, total	1	1	1	0	0	1
8.	Sample size per trial (median or mean, range or interquartile range)	1	1	0	1	1	1
9.	Homoeopathy type (4 categories or similar)	1	1	1	1	1	0
10.	Homoeopathic potency, categorised	1	0	0	0	1	1
11.	N different homoeopathic remedies/products	1	0	0	0	0	0
12.	Outcome type (symptoms, signs, global rating etc.)	0	0	0	1	0	0
	TOTAL	9	5	3	5	5	6

Suppl. Table 17 Data on individual trials

No.	Data on individual trials (0: not included, 1: included)	Linde 1997	Linde 1998	Cucherat 2000	Shang 2005	Mathie 2014	Mathie 2017
1	First author	1	1	1	1	1	1
2	Year published	0	1	1	0	1	1
3	N patients [analysed]	1	1	1	1	1	1
4	N patients > median of all trials	0	0	0	0	1	1
5	N lost to follow-up / n randomised + analysed	0	1	1	0	1	1
6	Indication group	1	1	0	1	1	1
7	Indication, brief	1	1	1	1	1	1
8	Indication, acute / chronic	0	0	0	0	0	1
9	Indication, detailed description	0	0	0	0	1	1
10	Intervention in homoeopathy group	1	1	1	1	1	1
11	Homoeopathic potency, categorised	0	0	0	0	1	1
12	Intervention in control group	0	1	1	0	0	0
13	Age & gender	0	1	0	0	1	1
14	Setting, country	0	1	0	0	1	1
15	Outcome	1	1	1	1	1	1
16	Outcome metric: dichotomous or continuous	1	0	0	0	1	1
17	Results, overall assessment	0	1	0	0	0	0
18	Response rates / mean improvement in both groups	0	1	0	0	1	1
19	Effect estimate + 95% CI, graphic	1	1	0	1	1	1
20	Effect estimate + 95% CI, numeric	0	1	0	0	1	1
21	p-value for effect estimate	0	0	1	0	0	0
22	Pilot / preliminary / feasibility study?	0	0	0	0	1	1
23	Quality / risk of bias rating, summarised	1	1	1	1	1	1
24	Quality / risk of bias rating, differentiated	0	0	0	0	1	1
25	Generation of allocation sequence	0	1	1	0	1	1
26	Concealment of allocation	0	1	0	0	1	1
27	Double-blinding	0	1	1	0	0	0
28	Blinding of patients	0	0	0	0	1	1
29	Blinding of evaluators	0	0	0	0	1	1
30	Intention-to-treat analysis	0	0	0	0	1	1
31	Incomplete outcome data	0	0	0	0	1	1
32	Selective outcome reporting	0	0	0	0	1	1
33	Selection bias after allocation	0	1	0	0	0	0
34	Statistical power calculation	0	0	0	0	0	1
35	Other sources of bias	0	0	0	0	1	1
36	Follow-up duration	0	1	0	0	1	1
37	Funding source, free text	0	0	0	0	1	1
38	Funding source, categorised "free of vested interest"	0	0	0	0	1	1
	TOTAL	9	21	12	8	31	33

Suppl. Table 18 Country of trials of the meta-analyses, in descending order of frequency

Country	Linde 1998		Mathie 2014		Mathie 2017		TOTAL*	
	N	Percent	N	Percent	N	Percent	N	Percent
United Kingdom	4	22.2%	5	22.7%	9	16.7%	18	19.1%
Germany	2	11.1%	1	4.5%	14	25.9%	17	18.1%
United States	1	5.6%	5	22.7%	3	5.6%	9	9.6%
India	1	5.6%	2	9.1%	3	5.6%	6	6.4%
France	0	0.0%	0	0.0%	6	11.1%	6	6.4%
Austria	1	5.6%	2	9.1%	2	3.7%	5	5.3%
Norway	2	11.1%	1	4.5%	2	3.7%	5	5.3%
Brazil	1	5.6%	1	4.5%	2	3.7%	4	4.3%
Israel	0	0.0%	2	9.1%	2	3.7%	4	4.3%
Italy	1	5.6%	0	0.0%	2	3.7%	3	3.2%
South Africa	1	5.6%	0	0.0%	2	3.7%	3	3.2%
Mexico	2	11.1%	0	0.0%	0	0.0%	2	2.1%
Netherlands	1	5.6%	1	4.5%	0	0.0%	2	2.1%
Australia	0	0.0%	0	0.0%	1	1.9%	1	1.1%
Honduras	0	0.0%	0	0.0%	1	1.9%	1	1.1%
Iran	0	0.0%	1	4.5%	0	0.0%	1	1.1%
Nicaragua	0	0.0%	1	4.5%	0	0.0%	1	1.1%
Ukraine	0	0.0%	0	0.0%	1	1.9%	1	1.1%
Unknown	1	5.6%	0	0.0%	4	7.4%	5	5.3%
TOTAL	18	100.0%	22	100.0%	54	100.0%	94	100.0%

*Multiple responses possible, as trials could be included in more than one meta-analysis. Data extracted from table data in Linde 1998, Mathie 2014, Mathie 2017. No data available for Linde 1997, Cucherat 2000, Shang 2005

Suppl. Table 19 Age groups, gender in trials of the meta-analysis

	Linde 1998 n = 18		Mathie 2014 n = 22		Mathie 2017 n = 54		TOTAL n = 94	
Age group	N	Percent	N	Percent	N	Percent	N	Percent
Children	4	22.2%	6	27.3%	4	7.4%	14	14.9%
Adults	10	55.6%	12	54.5%	30	55.6%	52	55.3%
Both/unknown	4	22.2%	4	18.2%	20	37.0%	28	29.8%
Gender								
Female	2	11.1%	3	13.6%	9	16.7%	14	14.9%
Male	0	0.0%	0	0.0%	2	3.7%	2	2.1%
Both/unknown	16	88.9%	19	86.4%	43	79.6%	78	83.0%

Suppl. Table 20 Indications in trials of the meta-analyses: ICD-10 chapters in descending order of frequency

ICD-10 Chapter	Linde 1997		Linde 1998		Cucherat 2000		Shang 2005		Mathie 2014		Mathie 2017		ALL*	
	N	Percent	N	Percent	N	Percent	N	Percent	N	Percent	N	Percent	N	Percent
J00-J99 Diseases of the respiratory system	20	22.5%	1	5.6%	6	35.3%	33	30.0%	1	4.5%	15	27.8%	76	24.5%
S00-T98 Injury, poisoning and certain other consequences of external causes	14	15.7%	1	5.6%	3	17.6%	10	9.1%	1	4.5%	8	14.8%	37	11.9%
K00-K93 Diseases of the digestive system	15	16.9%	0	0.0%	2	11.8%	13	11.8%	0	0.0%	4	7.4%	34	11.0%
M00-M99 Diseases of the musculoskeletal system and connective tissue	8	9.0%	3	16.7%	1	5.9%	8	7.3%	2	9.1%	5	9.3%	27	8.7%
N00-N99 Diseases of the genitourinary system	7	7.9%	1	5.6%	0	0.0%	8	7.3%	3	13.6%	6	11.1%	25	8.1%
A00-B99 Certain infectious and parasitic diseases	4	4.5%	4	22.2%	1	5.9%	7	6.4%	5	22.7%	1	1.9%	22	7.1%
G00-G99 Diseases of the nervous system	1	1.1%	4	22.2%	1	5.9%	4	3.6%	3	13.6%	4	7.4%	17	5.5%
R00-R99 Symptoms, signs and abnormal clinical and laboratory findings, not elsewhere classified	8	9.0%	1	5.6%	1	5.9%	4	3.6%	1	4.5%	2	3.7%	17	5.5%
O00-O99 Pregnancy, childbirth and the puerperium	3	3.4%	0	0.0%	1	5.9%	6	5.5%	0	0.0%	3	5.6%	13	4.2%
L00-L99 Diseases of the skin and subcutaneous tissue	3	3.4%	2	11.1%	1	5.9%	1	0.9%	3	13.6%	2	3.7%	12	3.9%
I00-I99 Diseases of the circulatory system	3	3.4%	1	5.6%	0	0.0%	3	2.7%	0	0.0%	2	3.7%	9	2.9%
F00-F99 Mental and behavioural disorders	0	0.0%	0	0.0%	0	0.0%	2	1.8%	2	9.1%	2	3.7%	6	1.9%
Z00-Z99 Factors influencing health status and contact with health services	0	0.0%	0	0.0%	0	0.0%	5	4.5%	0	0.0%	0	0.0%	5	1.6%
H60-H95 Diseases of the ear and mastoid process	1	1.1%	0	0.0%	0	0.0%	2	1.8%	1	4.5%	0	0.0%	4	1.3%
E00-E90 Endocrine, nutritional and metabolic diseases	1	1.1%	0	0.0%	0	0.0%	2	1.8%	0	0.0%	0	0.0%	3	1.0%
H00-H59 Diseases of the eye and adnexa	1	1.1%	0	0.0%	0	0.0%	1	0.9%	0	0.0%	0	0.0%	2	0.6%
V01-Y98 External causes of morbidity and mortality	0	0.0%	0	0.0%	0	0.0%	1	0.9%	0	0.0%	0	0.0%	1	0.3%
TOTAL	89	100.0%	18	100.0%	17	100.0%	110	100.0%	22	100.0%	54	100.0%	310	100.0%

*Multiple responses possible, as trials could be included in more than one meta-analysis

Suppl. Table 21 Indications in trials of the meta-analyses: ICD-10 three-digit diagnoses in descending order of frequency

ICD-3-digit diagnosis	Linde 1997		Linde 1998		Cucherat 2000		Shang 2005		Mathie 2014		Mathie 2017		ALL*	
	N	%	N	%	N	%	N	%	N	%	N	%	N	%
J30 Vasomotor and allergic rhinitis	6	6.7%	0	0.0%	1	5.9%	9	8.2%	0	0.0%	6	11.1%	22	7.1%
J11 Influenza, virus not identified	0	0.0%	0	0.0%	2	11.8%	11	10.0%	0	0.0%	2	3.7%	15	4.8%
J06 Acute upper respiratory infections of multiple and unspecified sites	9	10.1%	1	5.6%	0	0.0%	2	1.8%	1	4.5%	0	0.0%	13	4.2%
K91 Postprocedural disorders of digestive system, not elsewhere classified [Postoperative ileus]	6	6.7%	0	0.0%	1	5.9%	4	3.6%	0	0.0%	1	1.9%	13	4.2%
M79 Other soft tissue disorders, not elsewhere classified	2	2.2%	1	5.6%	0	0.0%	5	4.5%	1	4.5%	3	5.6%	12	3.9%
A09 Other gastroenteritis and colitis of infectious and unspecified origin	3	3.4%	2	11.1%	1	5.9%	4	3.6%	1	4.5%	0	0.0%	11	3.5%
J45 Asthma	2	2.2%	0	0.0%	1	5.9%	5	4.5%	0	0.0%	3	5.6%	11	3.5%
M06 Other rheumatoid arthritis	4	4.5%	2	11.1%	1	5.9%	2	1.8%	1	4.5%	1	1.9%	11	3.5%
G43 Migraine	1	1.1%	3	16.7%	0	0.0%	3	2.7%	2	9.1%	0	0.0%	9	2.9%
O80 Single spontaneous delivery	3	3.4%	0	0.0%	0	0.0%	5	4.5%	0	0.0%	0	0.0%	8	2.6%
K58 Irritable bowel syndrome	2	2.2%	0	0.0%	0	0.0%	3	2.7%	0	0.0%	2	3.7%	7	2.3%
N94 Pain and other conditions associated with female genital organs and menstrual cycle	2	2.2%	1	5.6%	0	0.0%	3	2.7%	1	4.5%	0	0.0%	7	2.3%
N95 Menopausal and other perimenopausal disorders	2	2.2%	0	0.0%	0	0.0%	1	0.9%	2	9.1%	1	1.9%	6	1.9%
T88 Other complications of surgical and medical care, not elsew. classif.	2	2.2%	1	5.6%	0	0.0%	0	0.0%	0	0.0%	3	5.6%	6	1.9%
B07 Viral warts	1	1.1%	1	5.6%	0	0.0%	2	1.8%	1	4.5%	0	0.0%	5	1.6%
G98 Other disorders of nervous system, not elsewhere classified	0	0.0%	0	0.0%	1	5.9%	0	0.0%	0	0.0%	4	7.4%	5	1.6%
N97 Female infertility	0	0.0%	0	0.0%	0	0.0%	0	0.0%	0	0.0%	5	9.3%	5	1.6%
R25 Abnormal involuntary movements	4	4.5%	0	0.0%	0	0.0%	1	0.9%	0	0.0%	0	0.0%	5	1.6%
H66 Suppurative and unspecified otitis media	1	1.1%	0	0.0%	0	0.0%	2	1.8%	1	4.5%	0	0.0%	4	1.3%
I64 Stroke, not specified as haemorrhage or infarction	2	2.2%	0	0.0%	0	0.0%	2	1.8%	0	0.0%	0	0.0%	4	1.3%
K08 Cysts of oral region, not elsewhere classified	3	3.4%	0	0.0%	0	0.0%	1	0.9%	0	0.0%	0	0.0%	4	1.3%
S93 Dislocation, sprain and strain of joints and ligaments at ankle and foot level	2	2.2%	0	0.0%	1	5.9%	1	0.9%	0	0.0%	0	0.0%	4	1.3%
T14 Injury of unspecified body region	2	2.2%	0	0.0%	0	0.0%	2	1.8%	0	0.0%	0	0.0%	4	1.3%
T30 Burn and corrosion, body region unspecified	1	1.1%	0	0.0%	1	5.9%	1	0.9%	0	0.0%	1	1.9%	4	1.3%
T59 Toxic effect of other gases, fumes and vapours	4	4.5%	0	0.0%	0	0.0%	0	0.0%	0	0.0%	0	0.0%	4	1.3%
T75 Effects of other external causes	2	2.2%	0	0.0%	0	0.0%	2	1.8%	0	0.0%	0	0.0%	4	1.3%
E66 Obesity	1	1.1%	0	0.0%	0	0.0%	2	1.8%	0	0.0%	0	0.0%	3	1.0%
F41 Other anxiety disorders	0	0.0%	0	0.0%	0	0.0%	1	0.9%	1	4.5%	1	1.9%	3	1.0%
I83 Varicose veins of lower extremities	1	1.1%	0	0.0%	0	0.0%	1	0.9%	0	0.0%	1	1.9%	3	1.0%

K29 Gastritis and duodenitis	3	3.4%	0	0.0%	0	0.0%	0	0.0%	0	0.0%	0	0.0%	3	1.0%
K31 Other diseases of stomach and duodenum	0	0.0%	0	0.0%	0	0.0%	3	2.7%	0	0.0%	0	0.0%	3	1.0%
L30 Other dermatitis	2	2.2%	1	5.6%	0	0.0%	0	0.0%	0	0.0%	0	0.0%	3	1.0%
R05 Cough	1	1.1%	0	0.0%	0	0.0%	1	0.9%	0	0.0%	1	1.9%	3	1.0%
R51 Headache	0	0.0%	1	5.6%	1	5.9%	1	0.9%	0	0.0%	0	0.0%	3	1.0%
T57 Toxic effect of other inorganic substances	0	0.0%	0	0.0%	0	0.0%	0	0.0%	0	0.0%	3	5.6%	3	1.0%
A06 Amoebiasis	0	0.0%	1	5.6%	0	0.0%	1	0.9%	0	0.0%	0	0.0%	2	0.6%
B20 Human immunodeficiency virus [HIV] disease resulting in infectious and parasitic diseases	0	0.0%	0	0.0%	0	0.0%	0	0.0%	2	9.1%	0	0.0%	2	0.6%
G93 Other disorders of brain	0	0.0%	1	5.6%	0	0.0%	1	0.9%	0	0.0%	0	0.0%	2	0.6%
H10 Conjunctivitis	1	1.1%	0	0.0%	0	0.0%	1	0.9%	0	0.0%	0	0.0%	2	0.6%
J00 Acute nasopharyngitis [common cold]	1	1.1%	0	0.0%	0	0.0%	1	0.9%	0	0.0%	0	0.0%	2	0.6%
J01 Acute sinusitis	0	0.0%	0	0.0%	0	0.0%	1	0.9%	0	0.0%	1	1.9%	2	0.6%
J02 Acute pharyngitis	1	1.1%	0	0.0%	0	0.0%	1	0.9%	0	0.0%	0	0.0%	2	0.6%
J20 Acute bronchitis	0	0.0%	0	0.0%	1	5.9%	0	0.0%	0	0.0%	1	1.9%	2	0.6%
J32 Chronic sinusitis	1	1.1%	0	0.0%	1	5.9%	0	0.0%	0	0.0%	0	0.0%	2	0.6%
J35 Chronic diseases of tonsils and adenoids	0	0.0%	0	0.0%	0	0.0%	1	0.9%	0	0.0%	1	1.9%	2	0.6%
J98 Other respiratory disorders	0	0.0%	0	0.0%	0	0.0%	2	1.8%	0	0.0%	0	0.0%	2	0.6%
K12 Stomatitis and related lesions	0	0.0%	0	0.0%	0	0.0%	1	0.9%	0	0.0%	1	1.9%	2	0.6%
K60 Fissure and fistula of anal and rectal regions	1	1.1%	0	0.0%	0	0.0%	1	0.9%	0	0.0%	0	0.0%	2	0.6%
L20 Atopic dermatitis	0	0.0%	0	0.0%	0	0.0%	0	0.0%	2	9.1%	0	0.0%	2	0.6%
L58 Radiodermatitis	0	0.0%	0	0.0%	0	0.0%	1	0.9%	0	0.0%	1	1.9%	2	0.6%
M25 Other joint disorders, not elsewhere classified	1	1.1%	0	0.0%	0	0.0%	1	0.9%	0	0.0%	0	0.0%	2	0.6%
N30 Cystitis	1	1.1%	0	0.0%	0	0.0%	1	0.9%	0	0.0%	0	0.0%	2	0.6%
N89 Other noninflammatory disorders of vagina	1	1.1%	0	0.0%	0	0.0%	1	0.9%	0	0.0%	0	0.0%	2	0.6%
N91 Absent, scanty and rare menstruation	0	0.0%	0	0.0%	0	0.0%	2	1.8%	0	0.0%	0	0.0%	2	0.6%
O99 Other maternal diseases classifiable elsewhere but complicating pregnancy, childbirth and the puerperium	0	0.0%	0	0.0%	0	0.0%	0	0.0%	0	0.0%	2	3.7%	2	0.6%
R47 Speech disturbances, not elsewhere classified	1	1.1%	0	0.0%	0	0.0%	1	0.9%	0	0.0%	0	0.0%	2	0.6%
T80 Complications following infusion, transfusion and therapeutic injection	0	0.0%	0	0.0%	0	0.0%	2	1.8%	0	0.0%	0	0.0%	2	0.6%
Z09 Follow-up examination after treatment for conditions other than malignant neoplasms	0	0.0%	0	0.0%	0	0.0%	2	1.8%	0	0.0%	0	0.0%	2	0.6%
Z98 Other postsurgical states	0	0.0%	0	0.0%	0	0.0%	2	1.8%	0	0.0%	0	0.0%	2	0.6%
A41 Other sepsis	0	0.0%	0	0.0%	0	0.0%	0	0.0%	1	4.5%	0	0.0%	1	0.3%
A97 Dengue	0	0.0%	0	0.0%	0	0.0%	0	0.0%	0	0.0%	1	1.9%	1	0.3%

F13 Mental and behavioural disorders due to use of sedatives or hypnotics	0	0.0%	0	0.0%	0	0.0%	1	0.9%	0	0.0%	0	0.0%	1	0.3%
F15 Mental and behavioural disorders due to use of other stimulants, including caffeine	0	0.0%	0	0.0%	0	0.0%	0	0.0%	0	0.0%	1	1.9%	1	0.3%
F90 Hyperkinetic disorders	0	0.0%	0	0.0%	0	0.0%	0	0.0%	1	4.5%	0	0.0%	1	0.3%
G80 Cerebral palsy	0	0.0%	0	0.0%	0	0.0%	0	0.0%	1	4.5%	0	0.0%	1	0.3%
I10 Essential (primary) hypertension	0	0.0%	0	0.0%	0	0.0%	0	0.0%	0	0.0%	1	1.9%	1	0.3%
I69 Sequelae of cerebrovascular disease	0	0.0%	1	5.6%	0	0.0%	0	0.0%	0	0.0%	0	0.0%	1	0.3%
J03 Acute tonsillitis	0	0.0%	0	0.0%	0	0.0%	0	0.0%	0	0.0%	1	1.9%	1	0.3%
L02 Cutaneous abscess, furuncle and carbuncle	0	0.0%	0	0.0%	1	5.9%	0	0.0%	0	0.0%	0	0.0%	1	0.3%
L08 Other local infections of skin and subcutaneous tissue	1	1.1%	0	0.0%	0	0.0%	0	0.0%	0	0.0%	0	0.0%	1	0.3%
L23 Allergic contact dermatitis	0	0.0%	0	0.0%	0	0.0%	0	0.0%	0	0.0%	1	1.9%	1	0.3%
L29 Pruritus	0	0.0%	0	0.0%	0	0.0%	0	0.0%	1	4.5%	0	0.0%	1	0.3%
L70 Acne	0	0.0%	1	5.6%	0	0.0%	0	0.0%	0	0.0%	0	0.0%	1	0.3%
M19 Other arthrosis	1	1.1%	0	0.0%	0	0.0%	0	0.0%	0	0.0%	0	0.0%	1	0.3%
M72 Fibroblastic disorders	0	0.0%	0	0.0%	0	0.0%	0	0.0%	0	0.0%	1	1.9%	1	0.3%
N64 Other disorders of breast	1	1.1%	0	0.0%	0	0.0%	0	0.0%	0	0.0%	0	0.0%	1	0.3%
O62 Abnormalities of forces of labour	0	0.0%	0	0.0%	0	0.0%	0	0.0%	0	0.0%	1	1.9%	1	0.3%
O66 Other obstructed labour	0	0.0%	0	0.0%	1	5.9%	0	0.0%	0	0.0%	0	0.0%	1	0.3%
O92 Other disorders of breast and lactation associated with childbirth	0	0.0%	0	0.0%	0	0.0%	1	0.9%	0	0.0%	0	0.0%	1	0.3%
R06 Abnormalities of breathing	0	0.0%	0	0.0%	0	0.0%	0	0.0%	0	0.0%	1	1.9%	1	0.3%
R45 Symptoms and signs involving emotional state	1	1.1%	0	0.0%	0	0.0%	0	0.0%	0	0.0%	0	0.0%	1	0.3%
R52 Pain, not elsewhere classified	1	1.1%	0	0.0%	0	0.0%	0	0.0%	0	0.0%	0	0.0%	1	0.3%
R53 Malaise and fatigue	0	0.0%	0	0.0%	0	0.0%	0	0.0%	1	4.5%	0	0.0%	1	0.3%
S00 Superficial injury of head	1	1.1%	0	0.0%	0	0.0%	0	0.0%	0	0.0%	0	0.0%	1	0.3%
S06 Intracranial injury	0	0.0%	0	0.0%	0	0.0%	1	0.9%	0	0.0%	0	0.0%	1	0.3%
S07 Crushing injury of head	0	0.0%	0	0.0%	0	0.0%	0	0.0%	1	4.5%	0	0.0%	1	0.3%
S83 Dislocation, sprain and strain of joints and ligaments of knee	0	0.0%	0	0.0%	1	5.9%	0	0.0%	0	0.0%	0	0.0%	1	0.3%
T56 Toxic effect of metals	0	0.0%	0	0.0%	0	0.0%	0	0.0%	0	0.0%	1	1.9%	1	0.3%
T81 Complications of procedures, not elsewhere classified	0	0.0%	0	0.0%	0	0.0%	1	0.9%	0	0.0%	0	0.0%	1	0.3%
Y83 Surgical operation and other surgical procedures as the cause of abnormal reaction of the patient…	0	0.0%	0	0.0%	0	0.0%	1	0.9%	0	0.0%	0	0.0%	1	0.3%
Z90 Acquired absence of organs, not elsewhere classified	0	0.0%	0	0.0%	0	0.0%	1	0.9%	0	0.0%	0	0.0%	1	0.3%
TOTAL	89	100.0%	18	100.0%	17	100.0%	110	100.0%	22	100.0%	54	100.0%	310	100.0%

*Multiple responses possible, as trials could be included in more than one meta-analysis

Suppl. Table 22 Data on possible funding-related vested interests in trials with data extractable for meta-analysis

Trial free of vested interests?	Mathie 2014		Mathie 2017		TOTAL	
	N	Percent	N	Percent	N	Percent
Yes	4	18.2%	11	20.4%	15	19.7%
No	8	36.4%	16	29.6%	24	31.6%
Unclear	10	45.5%	27	50.0%	37	48.7%
Total	22	100.0%	54	100.0%	76	100.0%

Extracted from table data in Mathie 2014 & 2017. No data in the other four meta-analyses

Suppl. Table 23 Tests for statistical heterogeneity

Author, year	Condition	N trials	Chi-squared	I-squared	Tau-squared (95%-CI)
Linde 1997/1999	Main analysis	89			0.43 (0.25-0.90); p = 2.4 x 10^{-13}
	Explicitly randomized	64			0.41 (0.23-0.87)
	Adequate conceal-ment of allocation	34			0.39 (0.22-0.87)
	Double-blinding	81			0.31 (0.18-0.71)
	Complete follow-up	28			0.44 (0.26-0.91)
	Explicitly randomized, multivariate*	[89]			0.28 (0.15-0.69)
	Jadad score >2	40			0.34 (0.19-0.78)
	IV score >4.5	34			0.40 (0.23-0.86)
	Jadad score >2 + IV score >4.5	26			0.36 (0.20-0.82)
Shang 2005	Main analysis	110	309, df 109, p < 0.0001	65%	
Mathie 2014	Main analysis			0% (95%-CI 0%-40%)	
Mathie 2017	Main analysis		p < 0.0001	65%	0.11, p < 0.0001
	After trim-and-fill			79%	

95%-CI: 95% confidence interval. *All other heterogeneity analyses in Linde 1997/1999 were univariate.

Assessments of bias and heterogeneity

Suppl. Table 24 Trials excluded from Shang 2005, reason for exclusion: Unavailable

**List of excluded homoeopathy studies**			**Language**	**Comment**
No.	Author, year	Publication type		
3	Beckmann-Reinhold 2007	Conference proceedings	English	
6	Campistrous-Lavaut 1999	Journal article	Spanish	Mathie 2013: A168.Placebo-controlled, individualised homoeopathy, not peer-reviewed. Excluded from Mathie 2014
19	Jansen 1997	Conference proceedings	English	
24	Lara-Marquez 1997	Conference proceedings	English	Linde 1998: Included in systematic review, insufficient data for meta-analysis (“*only preliminary abstract publication at a congress*”)
29	Manchanda 1997	Journal article	English	Mathie 2013: A210. Placebo-controlled, non-individualised homoeopathy, not peer-reviewed. Excluded from Mathie 2017
34	Ochoa-Bernal 1995	Journal article	Spanish	
41	Riveron-Garrote 1998	Journal article	Spanish	Mathie 2013: A174. Placebo-controlled, individualised homoeopathy, not peer-reviewed. Excluded from Mathie 2014
50	Straumsheim 1997	Journal article	English	Multiple publication: Included in Shang 2005 as Homoeopathy trial No. 87
53	Timofeeva 1997	Conference proceedings	English	Abstract published in European Neuropsychopharmacology 7 (Suppl 2) S291

Suppl. Table 25 Funnel plot inspection and associated tests

**Author, year**	**Trials**		**Funnel plot asymmetry?**	**Egger’s test**		**Other tests**
	Criteria	N		Asymmetry coefficient (95%-CI)	p-value	
Linde 1997 / 1999	All	89	Yes			p = 0.033*
Linde 1997 / Sterne 2001	Adequate concealment of allocation + Double-blinding	34	Yes			p = 0.014**<br>p < 0.001***
Shang 2005	All	110	Yes	0.17 (0.10-0.32)	p < 0.0001	None
Shang 2005 / Lüdtke 2008	High-quality	21	Yes	0.40	p = 0.17	None
Shang 2005 / Lüdtke 2008	High-quality + 8 largest trials	8	No data	1.15	p = 0.94	None
Mathie 2014	All	22	No	No data	p = 0.59	None
Mathie 2017	All	54	Yes	No data	p = 0.002	None

*General non-parametric selection model. **Rank correlation. ***Regression.

Suppl. Table 26 Trim-and-fill tests

Author, year	In order to increase p-value to	Statistics	Fictive additional trials with sample size = mean in meta-analysis	
			Trial result	N trials required
Linde 1997	p ≥ 0.05	Random-effects	Odds ratio = 1 (HOM=PLAC)	923
Linde 1997	p ≥ 0.05	Fixed-effects	Odds ratio = 1 (HOM=PLAC)	4511
Cucherat 2000	p > 0.01	p-value combination	p = 0.5 for HOM=PLAC	63
Cucherat 2000	p > 0.05	p-value combination	p = 0.5 for HOM=PLAC	155
Mathie 2017	Not stated**			11

*Trial sample size **Mathie 2017: p for at least one 'missing' study was < 0.001

Suppl. Table 27 Associations between methodological quality components and effect estimates

Author, year	Quality component	Uni- or multivariate	Rate of odds ratios* (95%-CI)	p-value	Significant?
Linde 1997 / 1999	Explicitly randomized	Univariate	0.66 (0.43-1.01)	No data	No
	Explicitly randomized	Multivariate	0.64 (0.43-0.94)	p = 0.03	Yes
	Adequate concealment of allocation	Univariate	0.71 (0.49-1.02)	No data	No
	Adequate concealment of allocation	Multivariate	0.84 (0.60-1.18)	No data	No
	Double-blinding	Univariate	0.24 (0.12-0.46)	p < 0.0001	Yes
	Double-blinding	Multivariate	0.26 (0.14-0.51)	p = 0.0002	Yes
	Complete follow-up	Univariate	1.31 (0.88-2.00)	No data	No
	Complete follow-up	Multivariate	1.23 (0.85-1.77)	No data	No
	Jadad scale >2	Univariate	0.56 (0.40-0.79)	No data	Yes
	Internal Validity score >4.5	Univariate	0.67 (0.47-0.97)	No data	Yes
	Jadad score >2 + Internal Validity score >4.5	Univariate	0.58 (0.40-0.79)	No data	Yes
Linde 1997 / Sterne 2001	English language	Univariate	0.73 (0.51-1.06)	p = 0.097	No
	English language	Multivariate	0.73 (0.55-0.98)	p = 0.038	Yes
	Medline-indexed	Univariate	0.61 (0.42-0.90)	p = 0.013	Yes
	Medline-indexed	Multivariate	0.91 (0.67-1.25)	p = 0.57	No
Shang 2005	English language	Univariate	0.73 (0.53-1.00)	p = 0.05	No
	Medline-indexed	Univariate	0.69 (0.50-0.94)	p = 0.019	Yes
	Double-blinding	Univariate	0.44 (0.22-0.87)	p = 0.017	Yes
	Adequate generation of allocation sequence	Univariate	0.76 (0.48-0.95)	p = 0.024	Yes
	Adequate concealment of allocation	Univariate	0.78 (0.57-1.07)	p = 0.117	No
	Intention-to-treat	Univariate	1.25 (0.87-1.80)	p = 0.225	No
	High quality trials	Univariate	(0.43-0.90)	p = 0.011	Yes
Mathie 2014	Publication free of vested interest	Univariate	No data	p = 0.87	No
Mathie 2017	Publication free of vested interest	Univariate	No data	p = 0.391	No

*Odds ratio for trials with criterion fulfilled (e.g., explicitly randomised) / Odds ratio for trials without criterion fulfilled (e.g., not explicitly randomised). Rate of odds ratios < 1 indicate smaller effects in trials with criterion fulfilled, compared to trials without criterion fulfilled.

Outcomes of this systematic review

Secondary outcomes

Suppl. Table 28 Meta-analysis results after sample restriction regarding one methodological quality component

Author, year	Sample restriction criterion	N trials	Statistic	Effect size metric	Effect estimate (95% confidence interval)	Favours homoeopathy	Significant?
Linde 1997	Adequate concealment of allocation	34	Random effects	Odds ratio	1.93 (1.51-2.47)	>1	Yes
Linde 1997/1999	Adequate concealment of allocation	34	Meta-regression	Odds ratio	2.00 (1.50-2.65)	>1	Yes
Linde 1997	Double-blinding stated	81	Random effects	Odds ratio	2.17 (1.83 - 2.57)	>1	Yes
Linde 1997/1999	Double-blinding stated	81	Meta-regression	Odds ratio	2.18 (1.83 - 2.60)	>1	Yes
Linde 1997	Adequate follow-up	28	Random effects	Odds ratio	3.18 (2.14-4.73)	>1	Yes
Linde 1997/1999	Complete follow-up	28	Meta-regression	Odds ratio	3.03 (2.12-4.33)	>1	Yes
Linde 1997	Medline-listed	23	Random effects	Odds ratio	1.70 (1.31-2.20)	>1	Yes
Linde 1997	Main outcome predefined	21	Random effects	Odds ratio	2.27 (1.62-3.18)	>1	Yes
Linde 1997/1999	Explicitly randomized	64	Meta-regression	Odds ratio	2.23 (1.81-2.75)	>1	Yes
Linde 1998	Medline-listed	11	Random effects?*	Rate ratio	1.22 (0.94-1.56)	>1	No
Mathie 2014	Free of funding-related vested interests	4	Random effects	Odds ratio	1.57 (1.05-2.36)	>1	Yes
Mathie 2017	Free of funding-related vested interests	11	Random effects	Standardised mean difference	0.19 (0.02-0.37)	>0	Yes

*Not unequivocal in publication

Suppl. Table 29 Meta-analysis results after sample restriction regarding more than one methodological quality component, ordered by number of components

Author, year	Sample restriction criterion	N trials	N com-ponents	Statistic	Effect size			p-value	Signifi-cant?
					Metric	Estimate (95% confidence interval)	Favours homoeopathy		
Linde 1997/1999	Jadad Score >2	40	3	Meta-regression	Odds ratio	1.81 (1.41-2.32)	>1	No data	Yes
Linde 1998	Double-blind + Medline-indexed + “no other obvious relevant flaws”	6	3	Random-effects	Rate ratios	1.12 (0.87-1.44)	<1	No data	No
Shang 2005 / Lüdtke 2008	High-quality + Medline-listed	14	4	Random-effects	Odds ratio	0.83 (0.60-1.14)	<1	0.25	No
	High-quality + English language	15	4	Random-effects	Odds ratio	0.93 (0.72-1.19)	<1	0.56	No
	High-quality + Intention-to-treat principle*	8	4	Random-effects	Odds ratio	0.69 (0.36-1.32)	<1	0.26	No
	High-quality + Medline-listed	14	4	Meta-regression	Odds ratio	1.29 (0.91-1.84)	<1	0.16	No
	High-quality + English language	15	4	Meta-regression	Odds ratio	0.94 (0.68-1.30)	<1	0.69	No
	High-quality + Intention-to-treat principle*	15	4	Meta-regression	Odds ratio	1.13 (0.60-2.15)	<1	0.70	No
Linde 1997/1999	Internal Validity Score >4.5	34	7	Meta-regression	Odds ratio	1.95 (1.50-2.59)	>1	No data	Yes
Mathie 2014	High-quality + Free of vested interests	1	8	Random-effects	Odds ratio	1.77 (0.66-4.72)	>1	No data	No
Mathie 2017	A- and B-rated trials + Free of vested interests	6	8	Random-effects	Standardised mean difference	0.02 (-0.32 to +0.28)	>0	No data	No

Lüdtke 2008 here interprets the quality component ‘Intention-to-treat principle’ as not included among the criteria for high-quality trials in Shang 2005, cf. Suppl. Table 11.
A- and B-rated trials: trials rated as having low or uncertain risk of bias in all seven domains (and high risk of bias in no domain) of Cochrane RoB, 2011 version.

Suppl. Table 30 Meta-analysis results after sample restriction regarding trial sample size with or without additional methodological quality components

Author, year	Sample restriction criterion	N trials		Heterogeneity (all trials)?	Effect size			Significant?
		All	After restriction		Metric	Estimate (95% confidence interval)	Favours homoeopathy	
	Sample size alone							
Mathie 2014	Sample size >median of all trials	22	11	No	Odds ratio	1.69 (1.30-2.21)	>1	Yes
Mathie 2017	Sample size >median of all trials	54	25	Yes	SMD	0.27 (0.12-0.42)	>0	Yes
	Sample size + Methodological quality							
Shang 2005	8 largest trials (n ≥ 98 per trial) + High-quality	110	8	Yes	Odds ratio	0.88 (0.65-1.19)	<1	No
Mathie 2014	Sample size > median + High-quality	22	3	No	Odds ratio	1.97 (1.16-3.38)	>1	Yes
Mathie 2017	Sample size > median + A- and B-rated trials	54	15	Yes	SMD	0.14 (-0.05 to +0.32)	>0	No

Statistic was random-effects for all analyses. SMD: Standardised mean difference

Suppl. Table 31 Cumulative MA with incremental removal of trials by risk-of-bias ratings: odds ratios

No.	Sample restriction criterion	N trials	Odds ratio (95% confidence interval)	Significant?
	Linde 1997 / 1999			
1	Jadad score ≥0 [All trials]	89	2.47 (2.06-2.97)	Yes
2	Jadad score ≥1	87	2.44 (2.02-2.93)	Yes
3	Jadad score ≥2	72	2.26 (1.87-2.73)	Yes
4	Jadad score ≥3	40	1.73 (1.44-2.08)	Yes
5	Jadad score ≥4	21	1.68 (1.29-2.18)	Yes
6	Jadad score =5	10	2.00 (1.37-2.91)	Yes
7	Internal Validity Score ≥1.0	89	2.47 (2.06-2.97)	Yes
8	Internal Validity Score ≥2.0	87	2.44 (2.02-2.93)	Yes
9	Internal Validity Score ≥2.5	78	2.42 (2.00-2.91)	Yes
10	Internal Validity Score ≥3.0	73	2.29 (192-2.74)	Yes
11	Internal Validity Score ≥3.5	65	2.35 (1.94-2.83)	Yes
12	Internal Validity Score ≥4.0	56	2.29 (1.88-2.80)	Yes
13	Internal Validity Score ≥4.5	43	2.09 (1.69-2.59)	Yes
14	Internal Validity Score ≥5.0	34	1.89 (1.53-2.35)	Yes
15	Internal Validity Score ≥5.5	24	1.71 (1.36-2.15)	Yes
16	Internal Validity Score ≥6.0	11	1.67 (1.10-2.54)	Yes
17	Internal Validity Score ≥6.5	7	2.02 (1.06-3.85)	Yes
18	Internal Validity Score =7.0	5	1.55 (0.77-3.10)	No
	Mathie 2014			
1	All trials	22	1.53 (1.22-1.91)	Ves
2	Remove C2.5 trials	20	1.60 (1.27-2.03)	Yes
3	Remove C2.1 trials	19	1.63 (1.29-2.06)	Yes
4	Remove C1.4 trials	17	1.66 (1.30-2.12)	Yes
5	Remove C1.3 trials	16	1.64 (1.28-2.09)	Yes
6	Remove C1.2 trials	15	1.65 (1.28-2.12)	Yes
7	Remove C1.1 trials	14	1.66 (1.28-2.17)	Yes
8	Remove C1.0 trials	12	1.63 (1.24-2.14)	Yes
9	Remove B6 trials	11	1.65 (1.24-2.18)	Yes
10	Remove B4 trials	10	1.64 (1.17-2.29)	Yes
11	Remove B3 trials	8	1.66 (1.17-2.34)	Yes
12	Remove B2 trials	6	1.77 (1.18-2.66)	Yes
13	B1 trials (= high quality trials)	3	1.98 (1.16-3.38)	Yes

Statistic was meta-regression for Linde 1997/1999 and random-effects models for Mathie 2014. Odds ratios >1 favour homoeopathy.

Suppl. Table 32 Cumulative MA with incremental removal of trials by risk-of-bias ratings in Mathie 2017

No.	Sample restriction criterion	N trials	Standardised mean difference (95% confidence interval)	Significant?
1	All trials	54	0.33 (0.21-0.44)	Yes
2	Retain C3.4 trials and better	52	0.31 (0.20-0.43)	Yes
3	Retain C2.5 trials and better	51	0.33 (0.21-0.44)	Yes
4	Retain C2.4 trials and better	50	0.33 (0.21-0.44)	Yes
5	Retain C2.3 trials and better	47	0.31 (0.19-0.43)	Yes
6	Retain C2.2 trials and better	45	0.32 (0.19-0.44)	Yes
7	Retain C2.1 trials and better	40	0.32 (0.18-0.45)	Yes
8	Retain C1.6 trials and better	38	0.30 (0.16-0.44)	Yes
9	Retain C1.5 trials and better	37	0.30 (0.16-0.44)	Yes
10	Retain C1.4 trials and better	36	0.31 (0.17-0.45)	Yes
11	Retain C1.3 trials and better	34	0.31 (0.16-0.46)	Yes
12	Retain C1.2 trials and better	32	0.30 (0.15-0.46)	Yes
13	Retain C1.1 trials and better	28	0.28 (0.11-0.45)	Yes
14	Retain C1.0 trials and better	27	0.27 (0.10-0.45)	Yes
15	Retain B6 trials and better	26	0.29 (0.11-0.47)	Yes
16	Retain B5 trials and better	22	0.34 (0.13-0.55)	Yes
17	Retain B4 trials and better	21	0.32 (0.10-0.53)	Yes
18	Retain B3 trials and better	14	0.33 (0.04-0.62)	Yes
19	Retain B2 trials and better	13	0.29 (-0.01 to +0.58)	No
20	Retain B1 trials and better	4	0.15 (-0.08 to +0.38)	No
21	Reliable evidence (high-quality trials)	3	0.18 (-0.09 to +0.46)	No

Random-effects models. >0 favours homoeopathy

Suppl. Table 33 Cumulative meta-analyses according to risk-of-bias ratings, rank-ordered categories in Cucherat 2000

No	Sample restriction criterion	N comparisons	P-value combination	Significant?
1	All trials	17	p = 0.000036	Yes
2	Double-blind	16	p = 0.000068	Yes
3	Double-blind + Dropout rate <10%	9	p = 0.0084	Yes
4	Double-blind + Dropout rate <5%	5	p = 0.082	No

Suppl. Table 34 Statistical adjustment for possible publication bias or small trial bias

Author, year	Effect size				
	Statistic	Metric	Estimate (95% confidence interval)	Favours homoeopathy	Signifi-cant?
Linde 1997	Random-effects model and non-parametric selection model	Odds Ratio	1.78 (1.03-3.10)	>1	Yes
Mathie 2017	Trim-and-fill	Standardised mean difference	0.16 (0.02-0.31)	>0	Yes

Suppl. Table 35 Combined sensitivity analyses

Author, year	Criteria	N trials	N quality components	Effect size			
				Metric	Estimate (95%-CI)	Favours homoeopathy	Signi-ficant?
Shang 2005	High-quality + 8 largest trials	8	4	Odds ratio	0.88 (0.65-1.19)	<1	No
Mathie 2017	A- and B-rated trials + sample size > median for all trials	15	8	Standardised mean difference	0.02 (-0.32 to +0.28)	>0	No

Statistics was random-effects analysis in both cases.

Subgroup analyses

Suppl. Table 36 Univariate associations between subgroups and effect estimates

Author, year	Subgroup	p-value	Significant?
Shang 2005	Duration of follow-up	p = 0.862	No
	Indication type (acute, chronic, prophylaxis)	p = 0.487	No
	Type of homoeopathy (4 groups)	p = 0.636	No
Mathie 2014	Sample size > median for all trials	p = 0.200	No
	Homoeopathic potencies ≥ 12C	p = 0.071	No
Mathie 2017	Sample size > median for all trials	p = 0.298	No
	Homoeopathic potencies ≥ 12C	p = 0.221	No

Rates of odds ratios were not reported for these associations.

Suppl. Table 37 Effect estimates in subgroups

Author, year	Criterion	N trials	Effect size		Favours homoeopathy	Signi-ficant?
			Metric	Estimate (95% confidence interval)		
Acute or chronic indication						
Mathie 2017	Acute	38	SMD	0.34 (0.19-0.49)	>0	Yes
Mathie 2017	Chronic	16	SMD	0.26 (0.11-0.41)	>0	Yes
Type of homoeopathy						
Linde 1997	Individualised*	13	Odds Ratio	2.91 (1.57-5.37)	>1	Yes
Linde 1997	Clinical	49	Odds Ratio	2.00 (1.60-2.51)	>1	Yes
Linde 1997	Isopathy	7	Odds Ratio	5.04 (2.24-11.32)	>1	Yes
Linde 1997	Complex	20	Odds Ratio	2.94 (2.12-4.08)	>1	Yes
Cucherat 2000	Individualised*	3	P value combination	p = 0.021	NA	Yes
Cucherat 2000	Non-individualised**	14	P value combination	p = 0.00011	NA	Yes
Mathie 2017	Combination product	9	SMD	0.20 (-0.05 to 0.45)	>0	No
Mathie 2017	Isopathy	7	SMD	0.21 (0.07-0.36)	>0	Yes
Mathie 2017	Complex	15	SMD	0.53 (0.27-0.79)	>0	Yes
Mathie 2017	Clinical	23	SMD	0.28 (0.09-0.47)	>0	Yes
Homoeopathic potency						
Linde 1997	High	31	Odds Ratio	2.66 (1.83-3.87)	>1	Yes
Linde 1997	High / medium	51	Odds Ratio	2.77 (2.09-3.67)	>1	Yes
Mathie 2014	≥12C	8	Odds Ratio	2.00 (1.38-2.88)	>1	Yes
Mathie 2014	<12C	14	Odds Ratio	1.30 (0.98-1.73)	>1	No
Mathie 2017	≥12C	21	SMD	0.21 (0.05-0.36)	>0	Yes
Mathie 2017	<12C	33	SMD	0.42 (0.25-0.60)	>0	Yes
Outcome metric in trial						
Mathie 2014	Continuous	16	Odds Ratio	1.45 (1.12-1.89)	>1	Yes
Mathie 2014	Continuous	16	SMD	0.21 (0.06-0.35)	>0	Yes
Mathie 2014	Dichotomous	6	Odds Ratio	1.80 (1.12-2.87)	>1	Yes
Mathie 2017	Continuous	31	SMD	0.36 (0.19-0.52)	>0	Yes
Mathie 2017	Dichotomous	23	Odds Ratio	1.67 (1.25-2.23)	>1	Yes

All analyses except for Cucherat 2000 were random-effects. *In addition, the Mathie 2014 meta-analysis was restricted to individualised homoeopathy. SMD: Standardised Mean Difference. **In Cucherat 2000 called ‘fixed prescription’

Additional data: Gartlehner 2022

Suppl. Table 38 Trials of the Mathie 2017 MA published 2002-2014, ordered by high-quality, risk-of-bias and trial number

No	Trial #	First author	Year	Registered?	Risk of bias	Risk-of-bias rating	High quality?
1	A103	Padilha	2011	1	Low	A	Yes
2	A272	Colau	2012	1	Low	A	Yes
3	A120	Singer	2010	1	Uncertain	B1	Yes
4	A047	Baker	2003	0	Uncertain	B2	No
5	A061	Cornu	2010	1	Uncertain	B2	No
6	A067	Frass	2005	0	Uncertain	B2	No
7	A093	Lewith	2002	0*	Uncertain	B2	No
8	A105	Paris	2008	1	Uncertain	B2	No
9	A137	Zabolotnyi	2007	0	Uncertain	B2	No
10	A275	Naidoo	2013	0	Uncertain	B2	No
11	A293	Malapane	2014	0	Uncertain	B4	No
12	A081	Jacobs	2007	0	High	C1.0	No
13	A113	Robertson	2007	0	High	C1.2	No
14	A086	Kim	2005	0	High	C1.3	No
15	A084	Khuda-Bukhsh	2005	0	High	C1.6	No
16	A089	Kotlus	2010	0	High	C2.2	No
17	A050	Belon	2006	0	High	C2.3	No
18	A136	Wolf	2003	0	High	C2.3	No
19	A101	Oberbaum	2005	1	High	C2.5	No
20	A085	Khuda-Bukhsh	2011	0	High	C5.0	No

*Not included in the analyses of Gartlehner 2022 (2)

Other information

Amendments, additional analyses and data

Suppl. Table 39 Amendments to the analysis protocol from 25.11.2020

Item in protocol	Amendment	Reason
8.7 Report time frame up to up to 31 October 2020	Updated literature searches from 1 November 2020 to 30 April 2023	Time period not covered by literature searches had increased to 30 months, cf. AMSTAR-2, item 4: “Conducted search within 24 months of completion of the review”.
8.10.2 Eligibility criteria for meta-analyses / Publication type ‘Additional analyses’	The term was changed from “additional analyses” to “additional publications”.	Resolving a logical inconsistency
8.10.2b Eligibility criteria for meta-analyses / “presenting results not included [in] the primary meta-analysis publication	The criterion was changed to “presenting information on methods, conduct or results not included in the primary meta-analysis publication”.	During assessment of publications for inclusion, comparing Cucherat 2000 and Boissel 1996 which both reported on the same meta-analysis, we realized the value of Boissel 1996 as source of additional information on methods and conduct of this meta-analysis.
9.1. Information sources / Databases	E. Embase: This database was omitted.	The use of this database would have incurred high costs, straining the project budget. Furthermore, having already searched the databases A-D and F-H and assessed the literature records for eligibility, we did not expect an Embase search to yield additional, potentially eligible literature records.
11.3 Data collection process: Involvement of individual reviewers	“Two reviewers (HJH, AG)” changed to “Two reviewers (HJH + [GSK, HK or AG])”	Practical
12.1 Data items / Eligibility criteria for trials in the meta-analyses	Design: Added “Randomisation”	Conformity with item 8.1 Eligibility criteria for meta-analyses / Design
12.9 Data items / Heterogeneity, meta-bias	Added items*:* Data on unavailable and unidentified trials	Studying the literature on publication bias, the relevance of actual data on unavailable and unidentified trials became clear.
13.0.C Heterogeneity, Meta-bias	Item 4 “Assessment of outcome reporting bias” was omitted	The item was already included in section ‘12.8 Assessment of risk of bias / methodological quality of trials’. In the included meta-analyses it was also assessed as risk of bias of individual trials not meta-bias.
13.3.A Subgroup analysis / Subgroup types	Subgroup analyses pertaining to “Indication: acute or chronic” were added to the list of subgroup analyses included in the systematic review.	Consistency of protocol. Subgroup analyses pertaining to acute or chronic indications are mentioned in the protocol under “Research questions” (item 7.1.B.) and “Data items” (12.11.C.4) but this analysis had not been specified under “Subgroup analyses” (13.3.A).
17. Confidence in cumulative evidence	Item B “risk of publication bias and outcome reporting bias”: “Outcome reporting bias” was shifted from Item B to Item A “study limitations (risk of bias)”	The forms of outcome reporting bias assessed in the meta-analyses were always assessable on the level of individual trials.

Suppl. Table 40 Additional analyses and data, not described in the protocol from 25.11.2020

Section/File	Additional analysis or data	Reason
Additional file 2	Additional comparative data from Shang 2005	See Article, Section 'Chronological overview'
Article, Section 'Additional data: Gartlehner 2022'	Additional analyses on Mathie 2017 by Gartlehner 2022 (2)	See Article, Section 'Additional data: Gartlehner 2022'
Article, Section 'Assessments of bias and heterogeneity'	Additional comparison of risk of bias in Mathie 2014 & 2017 to risk of bias of other trials, also evaluated with the Cochrane RoB tool (2011 version) (3)	Shang 2005 had already provided such a comparison of risk of bias to trials on conventional medicine, using a self-developed tool with 3 or 4 mandatory criteria. Mathie 2014 & 2017 having used a Cochrane tool with 7 criteria and thus more conservative than that of Shang 2005, we felt a comparison of risk of bias in Mathie 2014 & 2017 and in other trials using the same instrument was warranted.
Additional file 3, Section 1.7	Additional sensitivity analysis: Profile of evidence for significant positive of effects of homoeopathy beyond placebo in meta-analyses with low risk of bias (Additional file 3)	Our risk-of-bias assessment of the six meta-analyses with the ROBIS instrument resulted in a binary situation with risk of bias rated as low for 3 meta-analyses and high for 3. Consequently, a profile of evidence with conclusions on quality of evidence after restricting data to the 3 low-risk meta-analyses seemed warranted.
Additional file 3, Section 1.8	Addition to Confidence in cumulative evidence to Research question 1: Alternative hypothesis: no outcome difference between homoeopathy and placebo	Reading the literature on the meta-analyses in question and reflecting on the conclusions in Additional file 3, Section 1.7, an assessment of the alternative hypothesis seemed warranted.

References

1. Kleijnen J, Knipschild P, ter Riet G. Clinical trials of homoeopathy. *BMJ* 1991; **302**(6772): 316-23. https://doi.org/10.1136/bmj.302.6772.316

2. Gartlehner G, Emprechtinger R, Hackl M, et al. Assessing the magnitude of reporting bias in trials of homeopathy: a cross-sectional study and meta-analysis. *BMJ Evid Based Med* 2022; **27**: 345-51. http://dx.doi.org/10.1136/bmjebm-2021-111846

3. Sterne JAC, Savović J, Page MJ, et al. RoB 2: a revised tool for assessing risk of bias in randomised trials. *BMJ* 2019; **366**: l4898. https://doi.org/10.1136/bmj.l4898
