# Supplementary figures and images for: Efficacy of homoeopathic treatment: Systematic review of meta-analyses of randomised placebo-controlled homoeopathy trials for any indication
Source: Syst Rev. 2023 Oct 7;12:191. doi: 10.1186/s13643-023-02313-2 (PMC10559431; doi:10.1186/s13643-023-02313-2)

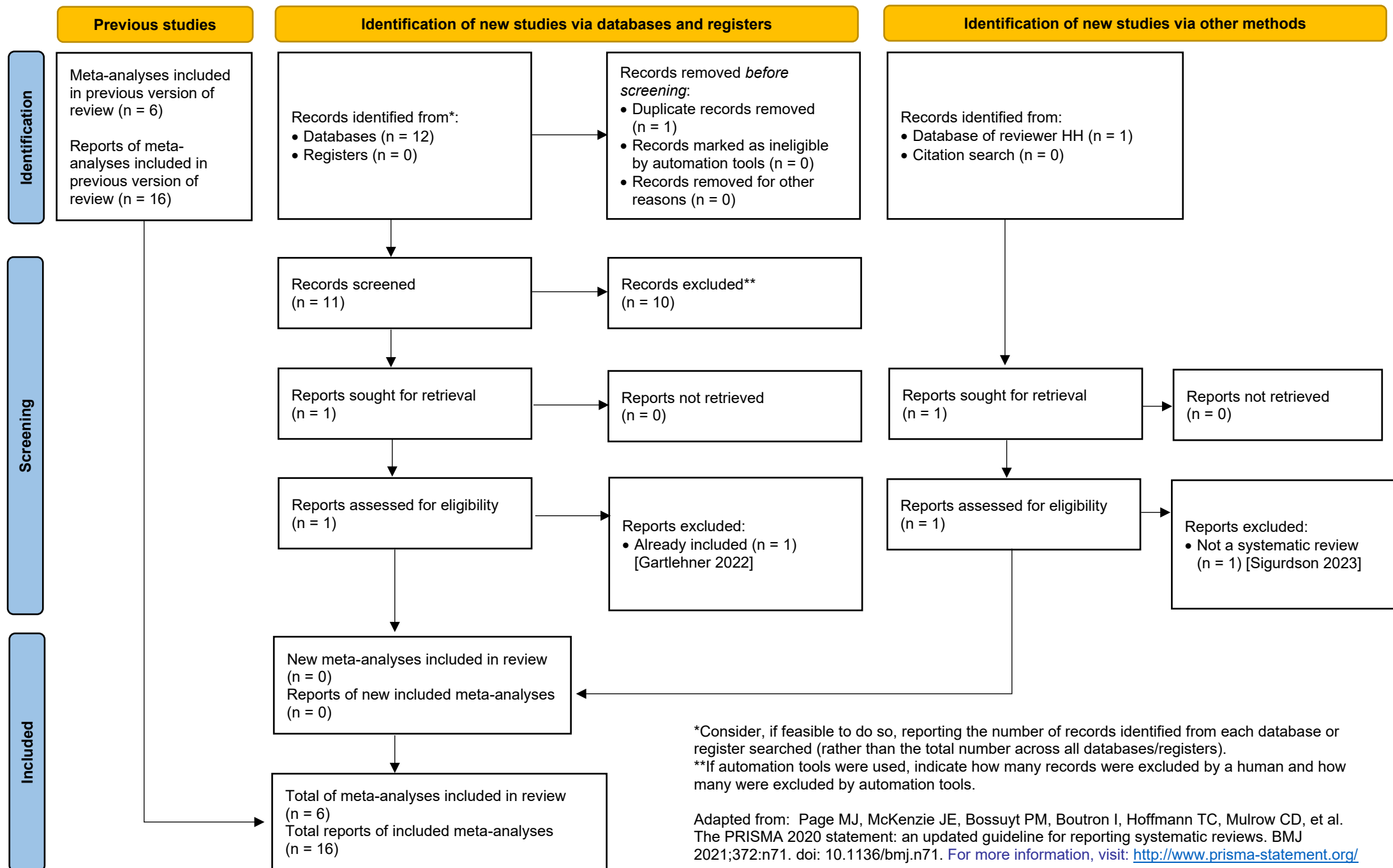

Supplement: Supplementary file 5 — Additional file 5. PRISMA 2020 flow diagram for updated systematic reviews which included searches of databases, registers and other sources. [file 13643_2023_2313_MOESM5_ESM.pdf]
